# Development and Bayesian Uncertainty Quantification of Coarse-Grained Models of Metals Based on Embedded Atom Method Potentials

**DOI:** 10.1021/acs.jctc.5c01322

**Published:** 2025-12-05

**Authors:** Abhishek T. Sose, Troy Gustke, Karteek K. Bejagam, Fangxi Wang, Aditya Savara, Sanket A. Deshmukh

**Affiliations:** † Department of Chemical Engineering, 1757 Virginia Tech, Blacksburg, Virginia 24060, United States; ‡ 6146 Oak Ridge National Laboratory, Oak Ridge, Tennessee 37830, United States

## Abstract

Coarse-grained (CG) molecular dynamics (MD) simulations have emerged as a powerful and cost-effective approach for modeling materials by simplifying atomic structures into CG beads. However, accurately parametrizing interatomic potential models (force fields, FFs) that can reliably reproduce material properties and quantifying the uncertainties associated with both the model parameters and their predictions remains a major challenge. In this study, we developed coarse-grained embedded atom method (CG EAM) potentials to model interatomic interactions in face-centered cubic (FCC) metals, including palladium (Pd), gold (Au), silver (Ag), copper (Cu), and platinum (Pt). The CG EAM potentials combine the physical interpretability of a traditional EAM with the computational efficiency of coarse-graining. We first employed a Particle Swarm Optimization (PSO) framework integrated with CG MD simulations to explore a 14-dimensional parameter space and identify CG EAM parameters that reproduce key physical, mechanical, and thermodynamic properties, such as cohesive energy, lattice constants, and elastic moduli. These parameters were subsequently refined using a Bayesian uncertainty quantification (BUQ) approach, which allowed the systematic assessment of uncertainties in both the FF parameters and the predicted properties. For all five metals, this framework yielded robust parameter ranges within which the predicted properties generally remained within their 95% confidence intervals. Overall, this integrated parameter optimization and BUQ approach provides an effective strategy for developing accurate and reliable interatomic potentials while offering a generalizable framework for designing both hard and soft materials with targeted properties.

## Graphical abstract

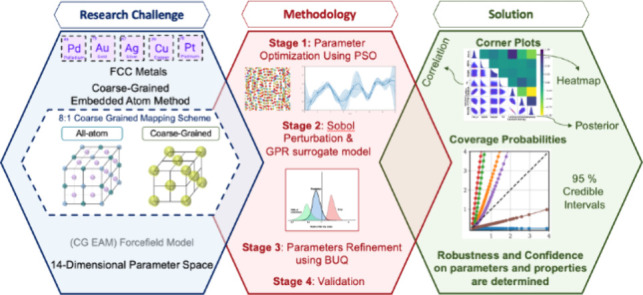

## 1 Introduction

Understanding and predicting the behavior of metals and metal nanoparticles (NPs) across multiple length and time scales is central to advancing technologies in catalysis, energy, and biomedicine.^1,2^ However, atomistic simulations of metals are computationally expensive, often limited to nanometer scales and nanosecond times, restricting their ability to capture mesoscale organization and deformation behavior.^3,4^ Coarse-grained (CG) models have emerged as powerful tools for simulating bulk metals and metal NPs.^5,6^ By grouping multiple atoms into beads, CG models substantially reduce system resolution, lowering computational costs and enabling simulations of systems containing millions of atoms over microsecond time scales.^7–9^ Although several studies have employed CG molecular dynamics (MD) simulations to investigate the self-assembly of grafted NPs, most have represented metal NPs as single spherical beads.^10^ This oversimplification sacrifices structural realism, diminishing the accuracy of the predicted properties and limiting the design of materials with tunable performance. Moreover, such representations are inadequate for modeling anisotropic nanoparticles, such as nanorods or faceted particles, where shape plays a critical role in governing assembly and mechanical response.^11^ These limitations highlight the need for CG models that preserve nanoparticle geometry and surface characteristics while accurately capturing their structural and mechanical behavior.^12^ In particular, shape- and facet-aware CG models of metal nanoparticles would enable systematic exploration of how nanoparticle shape, size, and surface chemistry collectively influence self-assembly, interfacial organization, and emergent mechanical properties, phenomena that remain computationally prohibitive in atomistic simulations. For instance, simulations of nanorod alignment under confinement, nanoparticle–polymer interactions, and size-dependent plastic deformation in metallic systems could all benefit from such realistic CG representations.^13^

In addition to nanoparticle assembly, CG models of metals can be employed to investigate mesoscale deformation and failure in nanostructures, grain-boundary processes, sintering and coalescence of nanoparticles, and fracture behavior in bulk metals and alloys.^14,15^ They can also be integrated with CG models of polymers to explore interfacial adhesion and the structural-mechanical properties of polymer–metal composites, which are critical for applications in flexible electronics, transportation, and construction.^16–18^

Due to the lack of dedicated CG models of metals, several studies have adopted a Quasi-Coarse-Grained-Dynamics (QCGD) approach, in which groups of metal atoms are represented as beads, and the interactions between them are derived by scaling all-atom (AA) embedded-atom method (EAM) potentials.^5,14,19–21^ In QCGD, the equations of motion are solved for representative atoms extracted from an atomistic microstructure, while scaling relationships from atomic-scale EAM potentials are used to define the interatomic forces. EAM potentials, a semiempirical framework based on density-functional theory (DFT), are widely used for AA MD simulations of metals.^22–24^ They accurately capture the coordination environment in metals, unlike pairwise potentials that depend solely on bond strength.^25^ However, a key limitation of QCGD is its reliance on AA EAM potentials to describe interactions between CG beads, introducing uncertainties that are rarely quantified.^14,20^

Quantifying uncertainty in EAM parameters that govern various interactions in CG models is essential, given the inherent loss of atomistic detail and the dependence on AA MD simulation outputs, which themselves carry simulation uncertainties.^26,27^ Bayesian Uncertainty quantification (BUQ) provides a systematic framework to capture these propagated uncertainties and estimate refined model uncertainties for both parameters and predicted properties.^28^ Recently, we demonstrated that BUQ performed using Ensemble Slice Sampling (ESS) and Affine-Invariant Ensemble Sampling (AIES) algorithms outperforms conventional approaches such as Metropolis-Hastings (MH), Gradient Search (GS), and Uniform Random Sampler (URS).^29^ Furthermore, we introduced a classification scheme to describe postoptimization model behavior as True-Tightened, True-Unchanged, or True-Loosened, based on the postoptimization model’s ability to reproduce a target property value and the change in the associated prediction uncertainty. A True-Tightened model accurately captures the property while yielding a narrower uncertainty distribution, consistent with the current ground-truth beliefs. A True-Unchanged model remains consistent with the ground truth with minimal change in uncertainty. A True-Loosened model also aligns with the ground truth but exhibits a broader uncertainty distribution after optimization. Overall, BUQ enhances the accuracy, reliability, and robustness of CG models by explicitly accounting for uncertainties originating from experimental data, AA MD simulations, and model parametrization.

Despite the progress achieved with QCGD, no consistent framework currently exists for developing CG metal models with quantified uncertainty. In this work, we address this challenge by combining BUQ with EAM-based coarse-graining to construct True-Tightened CG EAM models for five face-centered cubic (FCC) metals: copper (Cu), gold (Au), palladium (Pd), platinum (Pt), and silver (Ag). To the best of our knowledge, this represents the first uncertainty-aware CG EAM framework capable of systematically refining model parameters across multiple metals.

These metals, and their functionalized nanoparticles, play vital roles in biomedicine, energy, catalysis, sensing, and other technological applications.^30–35^ For each metal, 14 CG EAM parameters were optimized and subsequently refined to develop True-Tightened CG EAM models, while establishing a unified computational framework, shown in Figure 1, for the systematic and accurate development of CG models. The following sections are organized as follows: Section 2 describes the CG mapping scheme and the general framework for developing and quantifying uncertainty in EAM parameters; Section 3 presents the newly developed models for all five metals, including sensitivity analyses and BUQ of both parameters and properties; and Section 4 concludes the study with key findings and future directions.

**Figure 1:**
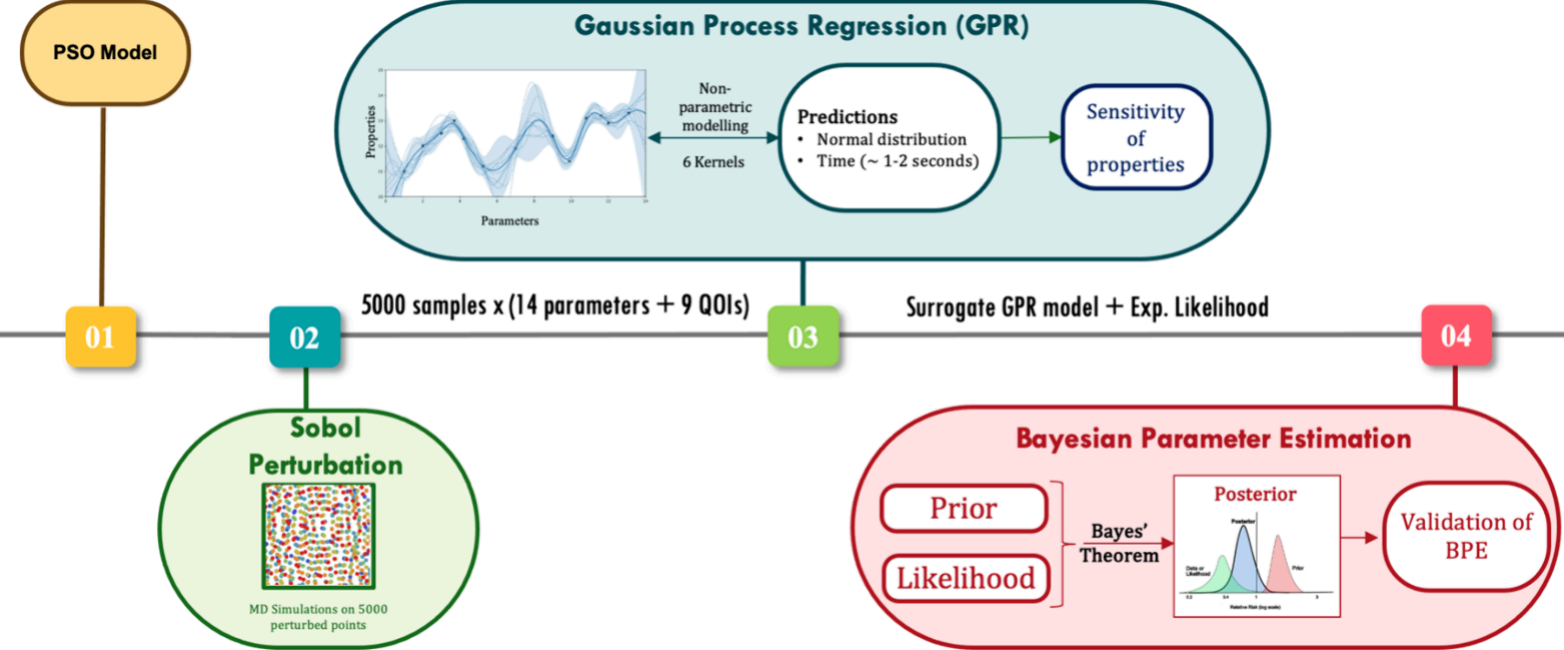
Schematic of the computational workflow for Bayesian Uncertainty Quantification (BUQ) of the Coarse-Grained Embedded Atom Method (CG EAM) model. Particle Swarm Optimization (PSO) was coupled with CG MD simulations to identify optimal EAM force field (FF) parameters. These parameters were then perturbed using Sobol sampling to generate 5,000 parameter sets for further CG MD simulations. The resulting data set trains a Gaussian Process Regression (GPR) surrogate model, which was used in BUQ analysis to compute robust posteriors and validate CG EAM potential accuracy.

## 2 Methodology

### 2.1 CG Metal Mapping Scheme

For all five FCC metals, an 8:1 mapping scheme, where eight metal atoms are represented by 1 CG metal bead, was employed to preserve the symmetry of the AA system, as illustrated in Figure 2.^19,21^ This level of coarse-graining was selected as a useful compromise: it is fine enough to capture essential lattice geometry and mechanical response, yet coarse enough to achieve an approximately 8-fold reduction in particle count, thereby significantly improving computational efficiency. In this mapping, all beads (both face-centered and corner) contribute equally to the total bulk mass. Consequently, the mass of a CG bead was defined as eight times the atomic mass of the corresponding metal atom (See Table S1 in Section S1.1 of the ESI) and was used to parametrize the CG EAM potentials. Thus, one unit cell in the CG model corresponds to a cluster of 2 × 2 × 2 unit cells in the AA system. The mass of each CG bead was calculated using eq 1:

1 $$\left(8\times \frac{1}{8}+6\times \frac{1}{2}\right)M_{\mathrm{CG}}=\left(8\times \frac{1}{8}+30\times \frac{1}{2}+13+12\times \frac{1}{4}\right)M_{\mathrm{AA}}=32M_{\mathrm{AA}},M_{\mathrm{CG}}=8M_{\mathrm{AA}}$$

where *M*_AA_ and *M*_CG_ are the masses of an atom and a CG bead, respectively. Figure 2 schematically illustrates how a single-unit cell of the CG system is mapped from 2 × 2 × 2 unit cells of the AA system.

**Figure 2:**
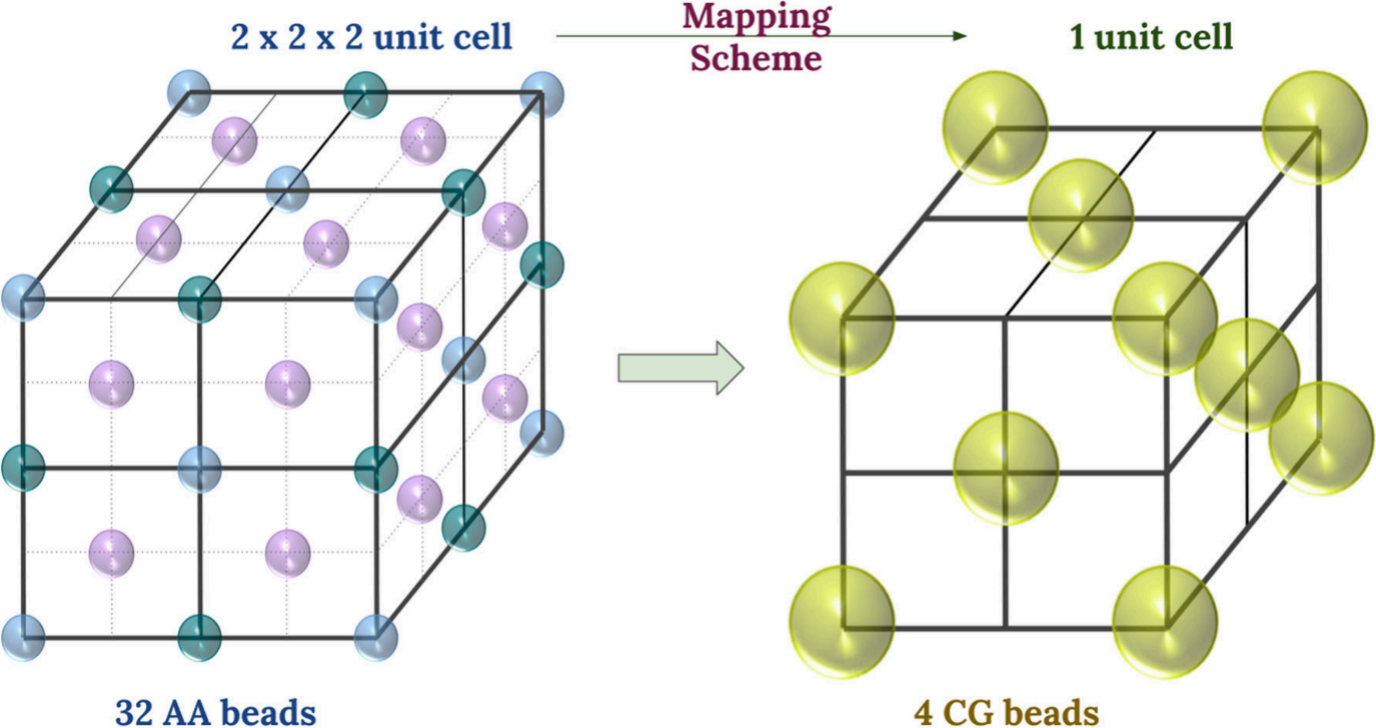
Schematic representation of the 8:1 mapping scheme employed in this study. Each CG bead represents eight atoms from the AA FCC structure, preserving the lattice symmetry. For Pd, each CG bead has a total mass of 851.36 au. In this mapping, blue beads on the faces and corners each contribute the full atomic mass of Pd (106.42 au), green edge beads contribute one-quarter of the atomic mass (26.605 au), and purple corner beads contribute one-half of the atomic mass (53.21 au) to the corresponding CG beads at the vertices. The same symmetry considerations apply to the face-centered atoms. Masses for all five metals are provided in Table S1 (Section S1.1 in the ESI).

### 2.2 CG EAM Model Parameters

The EAM formalism described by Marchal et al. for FCC metals^21,23^ was adopted in this study for the CG systems. The total energy of the system was expressed as the sum of the embedding energies (*F*_*i*_) and the pairwise interaction energies, as shown in eq 2.^22,36–38^

2 $$E_{\mathrm{total}}=\overset{N}{\underset{i=1}{\sum }}F_{i}\left(\rho_{i}\right)+\overset{N}{\underset{i=1}{\sum }}\underset{j>i}{\sum }\phi_{ij}R_{ij}$$

Here, *F*_*i*_, represents the energy required to embed a CG bead *i* into the host electronic density cluster, ρ_*i*_, generated by the surrounding beads, while ϕ_*ij*_ denotes the pairwise interaction energy between beads *i* and *j* separated by an interbead distance *R*_*ij*_. The detailed formulation of the EAM potential is provided in Section S1.2 of the ESI.^23^ A total of 14 parameters were developed for each of the five metals, governing both the embedding and pairwise interaction terms of the CG EAM potential.^22,37^ Parameters α, β, *A*, *B*, λ, and κ, contribute to the pairwise interbead potential term (eq S4 of the ESI), where *A* and *B* are the coefficients of repulsive and attractive components, respectively, and λ and κ are the corresponding cutoff distances between CG beads. Conversely, parameters ρ_e_, *F*_n3_, *F*_2_, *F*_e_, η, ρ_m_, and *f*_e_ play a major role in describing the embedding energy term (as in eqs S2, S3, and S5 of the ESI). Specifically, *F*_n3_, *F*_2_, and *F*_e_ represent the embedding energy coefficients from the lowest to highest electron density clusters. The lattice constant (*L*_c_) describes the spacing between repeating unit cells of the crystal and contributes to both embedding and pairwise terms.^22,29^

These input parameters collectively define the potential energy surface (PES) of each metal, governing its structural, physical, thermodynamic, and mechanical properties.^29^ The parameter ranges used for optimization for all five metals are listed in Tables S2–S6 of the ESI. To ensure the CG lattice remains approximately twice the AA lattice constant while allowing sufficient flexibility for parameter refinement toward target properties, a narrow optimization range centered around twice the lattice constant was employed.

### 2.3 Properties of Interest

We evaluated ten key properties of each of the five CG EAM metals to assess their accuracy and transferability: (i) Cohesive energy, defined as the energy required to completely separate the atoms in a solid metal, reflects the strength of interatomic bonding. Because each CG bead represents eight atoms, cohesive energies were scaled by a factor of 8. (ii) Density, measured in grams per cubic centimeter (g cm^–3^), quantifies the mass per unit volume of the metal. The elastic constants, (iii) C11, (iv) C12, and (v) C44, characterize the stiffness or rigidity of the metal in three directions. (vi) The bulk modulus measures resistance to uniform compression, indicating its ability to withstand volume changes under pressure. (vii) Poisson’s ratio represents the ratio of lateral contraction to longitudinal extension under tensile stress. Surface tensions at the (viii) 100, (ix) 110, and (x) 111 planes quantify the energy required to create new surfaces along these crystallographic directions and therefore reflect the surface stability. Together, these properties describe the bulk and surface behavior of metals and are essential for distinguishing their mechanical and structural characteristics.^39–45^ The target values used for model optimization and refinement for all five metals are provided in Tables S2–S6 in the ESI, with detailed calculation procedures described in Section S1.3 of the ESI.

### 2.4 Integration of Particle Swarm Optimization with MD Simulations

We integrated Particle Swarm Optimization (PSO) with CG MD simulations, performed using the LAMMPS simulation package, to develop CG EAM parameters for Pd, Au, Ag, Cu, and Pt. PSO is an iterative, population-based algorithm that explores the search space using a swarm of particles, where each particle represents a candidate set of the 14 CG EAM parameters.^7,46–48^ A detailed formulation of the PSO approach used in this study is provided in Section S1.4 of the ESI.^7,46,47,49^ We employed 128 particles to efficiently explore the high-dimensional (14-dimensional) parameter space, as using fewer particles would make the optimization overly sensitive to the initial guesses. This swarm size also lies well within the range typically reported for PSO algorithms, where the best performance in large-dimensional spaces (≈10 to 100 dimensions) is achieved with 70 to 500 particles.^50^ Each optimization involved at least 100 PSO epochs, ensuring convergence toward the desired target properties.

During parameter optimization, the 14 CG EAM parameters generated by PSO were used to construct the corresponding potential file, which was then employed in CG MD simulations to evaluate the relevant physical and mechanical properties. The computed properties were compared to target values to assess the fitness of each parameter set. CG MD simulations for cohesive energy, surface energies, and elastic constants were performed using energy minimization, whereas density calculations were conducted over 50 ps with a 1 fs time step to ensure stable and uninterrupted sampling. Additional simulation details are provided in Section S1.5 of the ESI.

After property evaluation, the absolute deviation of each computed property from its experimental target was calculated and used as the objective function, guiding PSO toward improved parameter sets. However, this initial optimization did not account for uncertainties in the target data or model predictions. Therefore, the BUQ framework was subsequently applied to refine the optimized models, enhance their physical realism, and establish statistically robust parameter ranges.

### 2.5 Bayesian Uncertainty Quantification (BUQ) Framework

The BUQ framework employed in this study consists of several sequential steps, outlined in detail below. The process begins with the development of a surrogate model trained on an initial data set, which is subsequently used to guide model refinement. Accordingly, the first step in the framework involves constructing a representative training set for the surrogate model.

#### 2.5.1 Sobol Sequence-Based Data Set Generation for Training the Gaussian Process Regression (GPR) Model

A training data set for the CG MD surrogate model was generated by perturbing the PSO-optimized CG EAM parameter sets for each metal using Sobol sequence-based perturbations.^51,52^ A total of 5000 parameter sets were produced through Sobol sampling, incorporating five levels of perturbation relative to the optimized parameters: 0.25%, 0.5%, 1%, 2%, and 4%, with 1000 points per level. This quasi-random sampling ensured uniform coverage of the 14-dimensional parameter space while maintaining statistical independence between samples. Each parameter was perturbed within bounds defined by the respective extent of perturbation, as shown in eq 3:

3 $$\theta_{\min}=\theta \left(1-\delta \right);,\theta_{\max}=\theta \left(1+\delta \right)$$

where δ represents the decimal associated with the percent perturbation variation (δ ∈ {0.0025, 0.0050, 0.0100, 0.0250, 0.0400 }), and θ denotes a given CG EAM parameter from the total set of 14 parameters. For each parameter, perturbation values were generated using a Sobol sequence according to eq 4.^51,52^

4 $$\theta_{i}=\theta_{\min}+\left(\theta_{\max}-\theta_{\min}\right){\mathrm{SP}}_{i}$$

where SP_*i*_ denotes the *i*th point in the Sobol sequence (*i* ∈ {1, 2, ..., *N*}). In this study, the Sobol sequence quasi-random numbers were generated within bounds of 0 to 1 for each dimension in the 14-dimensional parameter space, and the resulting perturbations were subsequently scaled for each parameter according to its defined extent. For each perturbation level, a set of 1000 parameter vectors, each containing 14 CG EAM parameters, were generated. These parameter sets were then used to perform CG MD simulations, producing a diverse and representative data set of parameter–property pairs for training the GPR models. The trained GPR models served as surrogate models during the BUQ analysis phase, enabling efficient exploration and refinement of the CG EAM parameter space.

#### 2.5.2 Gaussian Process Regression (GPR) Model Training

Gaussian Process Regression (GPR) is a highly effective and efficient method for making accurate predictions in high-dimensional spaces and has found broad applications in fields such as image recognition and material design.^53^ In this study, GPR models were trained using data from 5000 CG MD simulations generated via Sobol sequence sampling, where the CG EAM parameters served as input features, and the corresponding simulated properties served as outputs. An important advantage of GPR is its ability to provide prediction uncertainties along with mean estimates, which were leveraged in the BUQ framework to propagate surrogate-model uncertainty throughout the refinement process.

To identify the optimal kernel for each property, six kernel functions were tested during GPR training: (i) Radial basis function (RBF), (ii) Exponential, (iii) Matern 32, (iv) Matern 52, (v) RBF + RBF, and (vi) Cosine. These kernels were used because they encompass a wide spectrum of varying degrees of smoothness characteristics.^54,55^ Prior to model training, outliers exceeding three standard deviations from the mean of the property distribution were removed to mitigate numerical instability and improve model generalization. For each metal, the filtered data set was divided into 90% training and 10% validation subsets, and a 5-fold cross-validation was performed to determine the best-performing kernel.^56,57^ After kernel selection, the GPR model was trained using all of the remaining data points and subsequently employed as a surrogate model during Bayesian posterior exploration, as described in the following section.

#### 2.5.3 Bayesian Inference

As in our recent study,^29^ the likelihood function’s distribution was based upon the uncertainties associated with both the experimental measurements and the uncertainties associated with the theoretical model outputs. These are associated with an error term that consists of a summation of these possible errors given by

5 $$\epsilon =\epsilon_{M}+\epsilon_{\mathrm{SM}}$$

where ϵ_M_ is the measurement error from experiment and ϵ_SM_ is the modeling error of surrogate models used in the computational framework. As experimentally measured property values also have uncertainty, the mean experimentally measured value of any property differs from *Y*_true_ and is given by *Y*_obs_,

6 $$Y_{\mathrm{obs}}=Y_{\mathrm{true}}+\epsilon_{M}$$

It is convenient and often reasonable to assume that the total error can be approximated as a Gaussian distribution (i.e., ϵ ∼ *N*(0, σ_error_^2^)). When a Gaussian distribution is used for the error term, the likelihood takes the form of a multivariate normal distribution. However, assuming a multivariate Gaussian is not necessary for Bayesian UQ. In fact, one of the strengths of Bayesian UQ is the ability to examine arbitrary high-dimensional correlation structures between uncertainties. Here, uncorrelated normal distributions were used for the uncertainties of the property values.

One goal of the UQ performed here was to characterize the robustness of the refined CG EAM parameters vector, θ, by assessing the feasible ranges for θ based on the experimental/DFT observations, *Y*_obs_. The feasible ranges can be assessed from the posterior distributions for individual parameters, calculated through Bayes theorem:

7 $$p\left(\theta |Y_{\mathrm{obs}},M\right)=\frac{p\left(Y_{\mathrm{obs}}|\theta ,M\right)\cdot p\left(\theta |M\right)}{p\left(Y_{\mathrm{obs}}|M\right)}$$

where *p*(θ|*Y*_obs_, *M*) is the posterior probability distribution of the FF parameters given the observed data and a model, *M*, *p*(*Y*_obs_|θ, *M*) is the likelihood of observing the experimental data from the given model, *p*(θ|*M*) is the prior distribution of the FF parameters for the given model, and p­(*Y*_obs_|*M*) is the evidence of the observed data. In this study, the prior distribution for the parameters is given by a bound uniform distribution based on a 4% perturbation of the final set provided by PSO for each parameter (the 4% was chosen to stay within the training bounds of the GPR surrogate predictions).

In order to find the most probable parameter set, the maximum of the posterior distribution was sought by repeated evaluations of the model using Markov Chain Monte Carlo (MCMC) techniques. The high-dimensional parameter space was explored with enhanced time and resource efficiency relative to running CG MD simulations by using the surrogate GPR models. For posterior exploration, the ensemble slice sampling (ESS) algorithm with 56 walkers was utilized^58^ with a sampling of 2,000,000 points. ESS is effective and efficient for such high dimensional parameter space exploration.^29^ The Python package PEUQSE was utilized to perform the ESS sampling and to approximate and visualize the posterior distributions of the CG EAM parameters.^9^ Convergence of ESS samplings was based on an integrated autocorrelation time (ACT) metric.^58^

## 3 Results and Discussion

### 3.1 CG EAM Model Parameters Derived Using the PSO-Integrated MD Approach

The 14 PSO-developed CG EAM parameters for all five metals and their corresponding properties are summarized in this section. The target values used for model optimization are provided in Tables S2–S6 of the ESI and were obtained from references 42 and 59–77 . The models based on these PSO-optimized parameters successfully reproduced the experimental bulk, surface, and elastic properties, as shown in Figure 3, thereby serving as reliable starting points for subsequent BUQ refinement and parameter robustness analysis. Furthermore, Tables S2–S6 of the ESI present the properties predicted by the PSO-optimized (pre-refinement) CG EAM parameters for each metal, along with their comparison to target experimental values and associated errors. Tables S7–S11 in the ESI further demonstrate that these CG EAM models outperform most existing AA EAM models across nearly all evaluated properties for all five metals.

**Figure 3:**
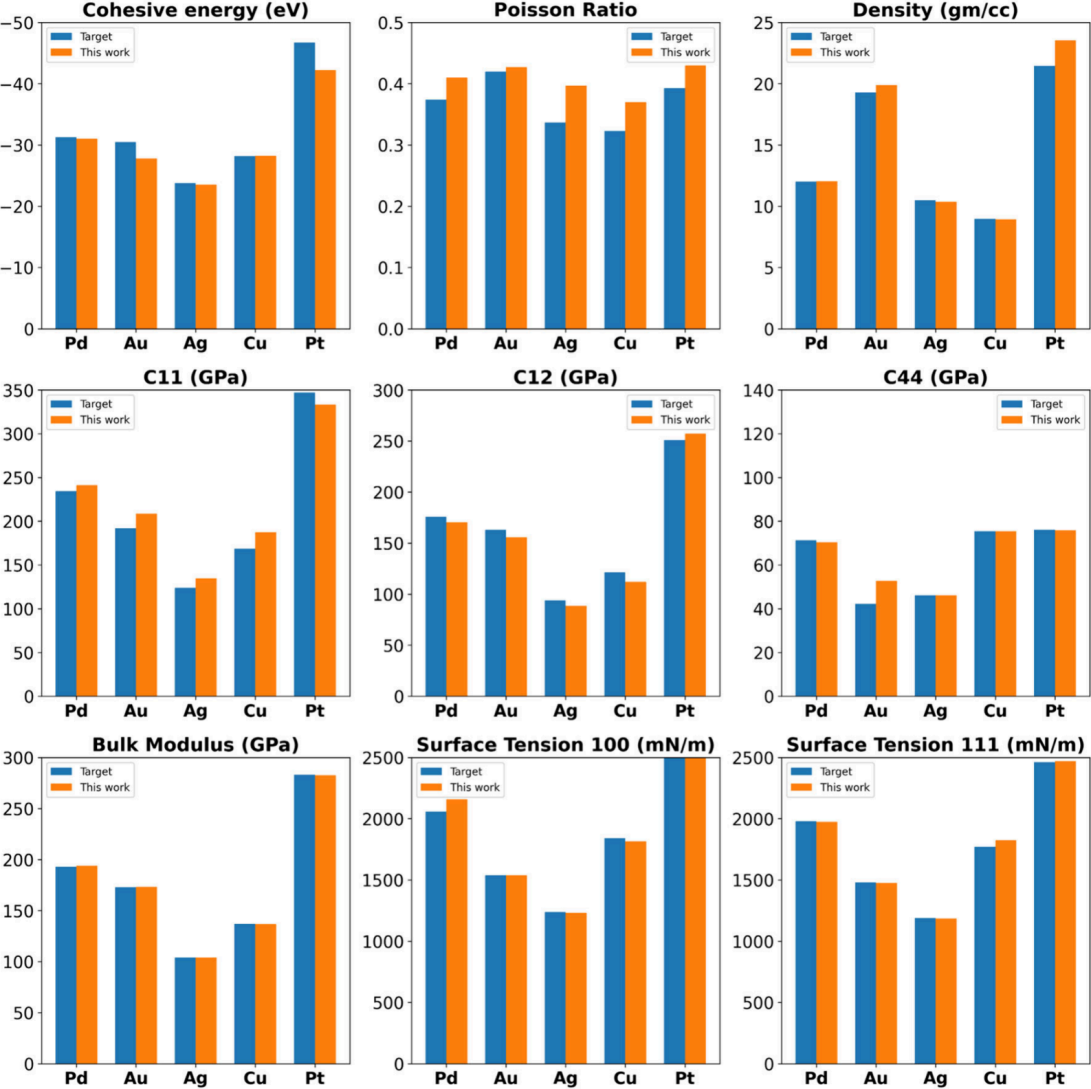
Comparison of properties predicted by the developed CG EAM models in this work (orange bar) with the experimental target properties (blue bar) prior to any Bayesian refinement.

### 3.2 Analysis of Property Trends in Training Data from CG MD Simulations

To generate a rich and informative training data set for developing accurate GPR surrogate models, the final PSO-optimized CG EAM parameter sets were systematically perturbed using Sobol sequences within ±0.25%, ±0.5%, ±1%, ±2%, and ±4% ranges.^51^ This procedure produced 5,000 diverse parameter sets, providing denser sampling near the optimized values to enhance local model accuracy while maintaining sufficient variation for global coverage and mitigating overfitting. To assess how the extent of these perturbations influenced the target properties derived from CG MD simulations, raincloud plots were constructed for each property (Figure 4 and Figures S1–S10 in the ESI). These plots, which combine violin and box plots, depict property distributions, interquartile ranges, and outliers, offering clear insight into the sensitivity of each property to parameter variations. Based on prior findings that perturbations beyond ±4% contribute minimally to uncertainty quantification,^29^ the selected perturbation bounds, and sampling density ensured a robust and representative data set for GPR model training.

**Figure 4:**
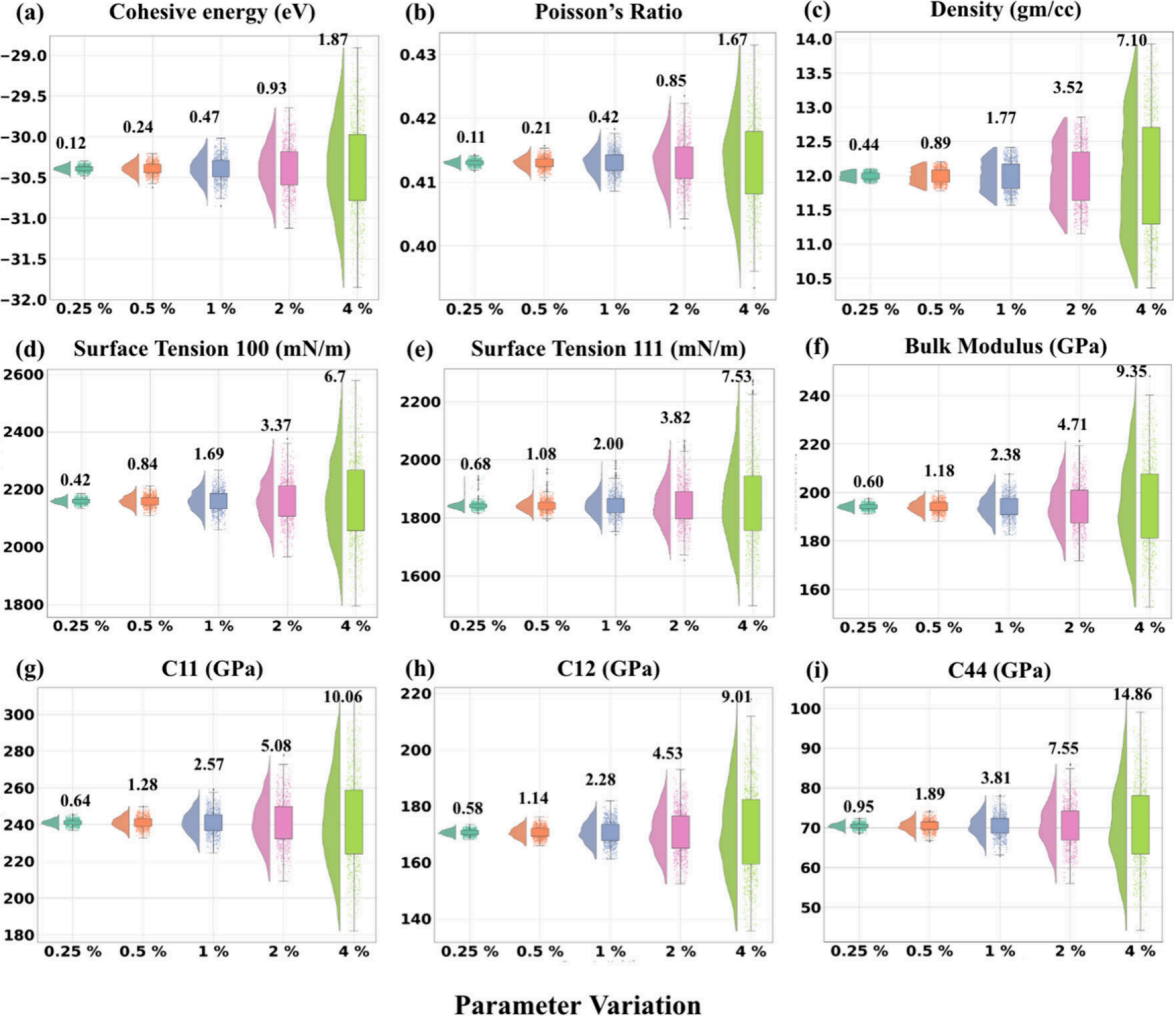
Raincloud plot representing the variation of properties, specifically (a) cohesive energy (in eV), (b) Poisson’s ratio, (c) density (in g/cm^3^), (d) surface tension at 100 surface (in mN/m), (e) surface tension at 111 surface (in mN/m), (f) bulk modulus (in GPa), (g) C11 (in GPa), (h) C12 (in GPa), and (i) C44 (in GPa) calculated via CG MD simulations on Sobol perturbed input parameters, shown here for the Pd model as an example. Additional raincloud plots are provided in Figures S1–S10 in the ESI for Au, Cu, Ag, and Pt. The numbers annotated in the plots represent % Coefficient of Variation.

### 3.3 Gaussian Process Regression (GPR) Model

Table S12 of the ESI summarizes the *R* ^2^ values for each property along with their respective best performing kernels, while Figures S11–S15 of the ESI present the parity plots for all five metals. Overall, the GPR models achieved high accuracy across most properties, with *R* ^2^ values ranging from ∼0.9 to 0.999. However, lower *R* ^2^ < 0.80 were observed for surface tension at (111) surface (ST111) in Au, Pd, Cu, and Pt; surface tension at (100) surface (ST100) for Cu and Pt; and Poisson’s ratio for Pt. This trend aligns with earlier reports indicating that EAM potentials often underestimate surface energies due to reduced electron density at metal surfaces and their inherent inability to capture electron density gradients.^29,36–38,78–80^ Consequently, these limitations are expected to propagate into the CG EAM potentials derived from them. During the BUQ refinement and analysis, these less accurately reproduced properties (*R* ^2^ < 0.80) were excluded to avoid introducing additional uncertainty or instability into the posterior parameter estimation. Nonetheless, these properties were recalculated during the final validation phase using 100 randomly selected CG EAM parameter sets, as detailed in Sections S5 and S6 in the ESI, to ensure that the final models’ overall performance could still be assessed for these challenging properties.

### 3.4 Sobol Sensitivity Indices

The GPR models that demonstrated high predictive accuracy were subsequently used to perform variance-based sensitivity analysis.^29^ Specifically, the influence of each CG EAM parameter on the variation of predicted properties was quantified using Sobol indices, which measure the contribution of individual parameters and their interactions to the overall output variance. The resulting sensitivity distributions are illustrated in Figure 5.

**Figure 5:**
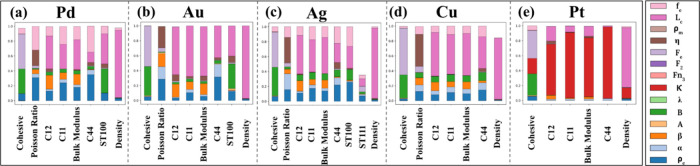
First-order Sobol sensitivity indices representing the sensitivity of all 14 input parameters to their respective outputs for all metals, *viz*. (a) Pd, (b) Au, (c) Ag, (d) Cu, and (e) Pt. There are inherent differences between the materials.

As shown in Figure 5, most properties for each metal were highly sensitive to the lattice constant (*L*_c_), which represents the scaled equilibrium bond distance between two CG beads.^29,78^ For all metals except Pt, the second most sensitive parameter was ρ_e_, which here can be interpreted as a *coarse-grained representation of the local electron density* experienced by a CG bead when interactions with neighboring beads are balanced and the embedding energy is minimized. This indicates that, across all CG EAM models, the spatial arrangement of CG beads and their interbead interactions play a critical role in determining material properties, a relationship that the model captures effectively.

The cohesive energy in all metals exhibited strong sensitivity to *F*_e_, a coefficient that governs contributions from high electron-density regions in the embedding energy term, and to *B*, the attraction coefficient in the pair potential term. This finding suggests that the CG models effectively capture the dual influence of collective electron distribution and interbead attractive interactions in determining cohesive energy. This observation aligns with the known origin of metallic cohesion, which arises primarily from the attraction between positively charged metal ions and the surrounding delocalized electron sea.^25,81–83^

For Ag, the first-order Sobol indices for ST111 were substantially below 1, indicating a strong dependence on multiple correlated parameters. This observation further supports the persistence of a well-documented limitation of EAM potentials, namely their reduced accuracy modeling surface tensions due to their inability to fully capture electron-density gradients near surfaces.^84,85^

In Ag, Pd, and Cu, most properties showed pronounced sensitivity to *f*_e_, the coefficient representing the collective electron distribution among individual beads. This trend suggests that the CG models successfully encode the effects of collective electron behavior, enabling accurate reproduction of the physical properties of these metals within a CG representation.

Interestingly, for Pt, most properties were dominated by the sensitivity to κ, the cutoff parameter governing the repulsive term in the pair potential. This result highlights the importance of short-range repulsive interactions in determining Pt’s behavior. Additionally, Poisson’s ratio exhibited notable sensitivity to η (after *L*_c_ and ρ_e_), suggesting that regions of higher collective electron density exert a stronger influence on the elastic response captured by the model.

### 3.5 Bayesian Uncertainty Quantification Analysis

#### 3.5.1 Posterior Distributions for Parameters and Properties

In this work, the CG EAM models were refined using the BUQ framework with sampling performed via the Ensemble Slice Sampling (ESS) algorithm (Section S4.1 of the ESI). One primary goal of BUQ is to determine the most probable parameter set or *maximum a posteriori* (MAP) for each parameter, which represents a refined model.^29^ Beyond MAP estimation, the BUQ provides the posterior distribution for each system, which characterizes the probability distribution landscape across all feasible parameter combinations, thereby representing uncertainty around the refined models. These multidimensional posterior distributions capture parameter–parameter, parameter–property, and property–property correlations and may exhibit multiple distinct modes, indicating the presence of alternative high-probability solutions consistent with the data.

The integrated autocorrelation times confirmed convergence of the ESS sampling (Figure S16 of the ESI). In several metals, the resulting posterior distributions displayed multimodal behavior, suggesting multiple distinct high-probability solutions or parameter configurations. Figure 6 presents representative corner plots summarizing these results for selected parameters and properties across all 5 metals. The diagonal panels show marginal distributions of individual parameters, where bimodal or multimodal patterns are evident; the scatter plots below the diagonal depict correlations between parameter pairs, parameters and properties, and property pairs; while the heat maps above the diagonal display the corresponding Pearson correlation coefficients.

**Figure 6:**
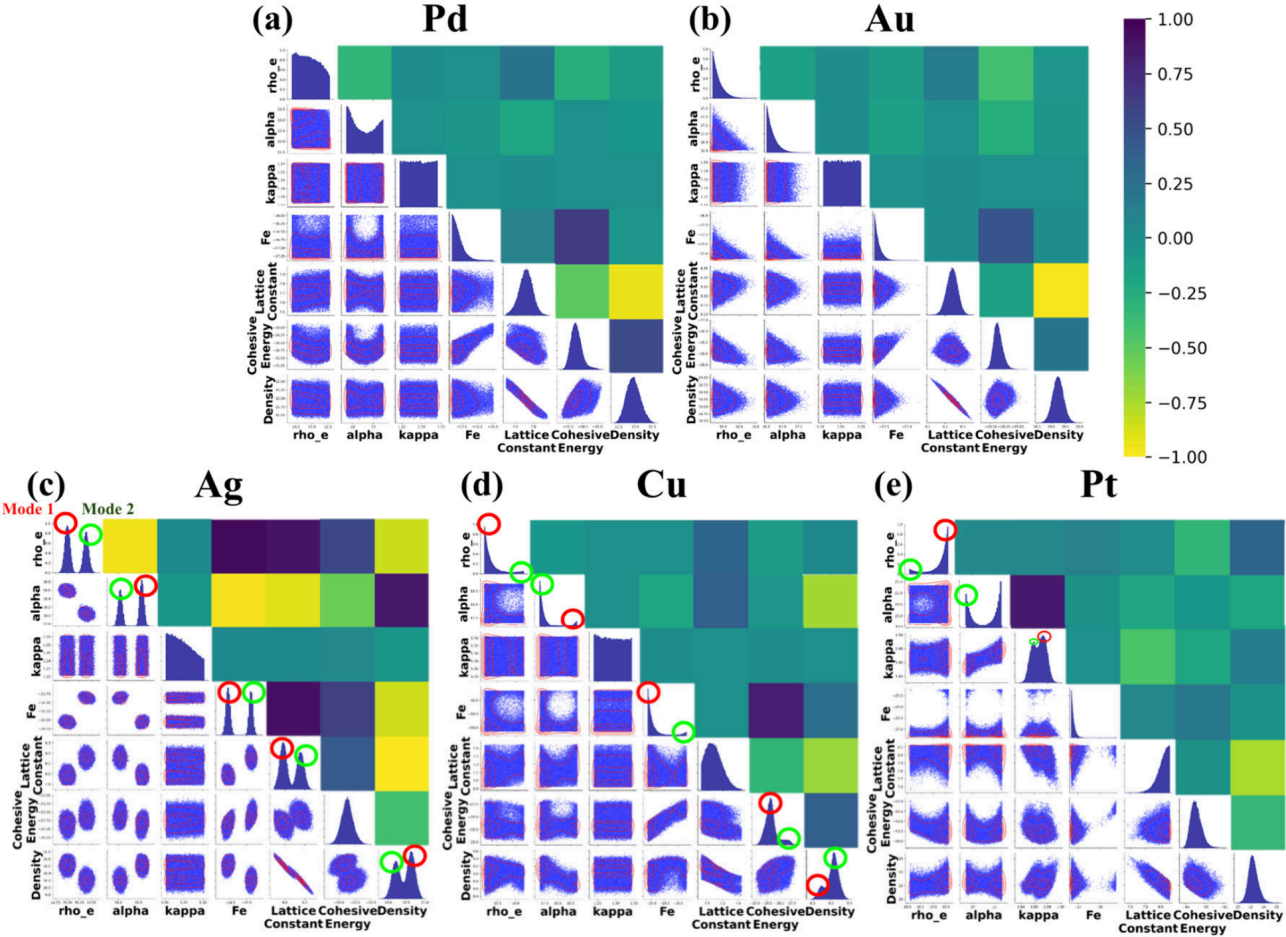
Corner plots and heatmaps of Pearson correlation coefficients for the selected parameters and properties for all five metals, *viz*. (a) Pd, (b) Au, (c) Ag, (d) Cu, and (e) Pt, with densities of posterior distributions as a function of the *x*-axis variables shown along the panels running diagonally from top left to bottom right. In the diagonal panels, Mode 1 and Mode 2 are marked with red and green circles, respectively. It can be seen that for some metals, there are the two distinct modes observed for certain parameters and certain properties. Table S15 of the ESI provides a catalogue of shapes observed in scattered plots including their corresponding symbols and names.

Comprehensive results are provided in the ESI: Figures S17–S21 and Figures S22–S26 show corner plots and heatmaps for Pearson correlation coefficients, respectively, for 14 CG EAM parameters for all five metals. Figures S27–S31 and Figures S32–S36 show corner plots and heatmaps for Pearson correlation coefficients, respectively, for all properties predicted by the GPR model for all five metals. Figures S37–S41 and Figures S42–S46 show corner plots and heatmaps for Pearson correlation coefficients, respectively, for all 14 CG EAM parameters and properties predicted by the GPR model for all five metals.

The patterns observed in the scatter plots below the diagonals in Figure 6 are cataloged in Table S15 of the ESI, along with their corresponding names and symbols. Circular patterns generally indicate low linear correlation, whereas elongated or diagonal elliptical shapes suggest strong positive or negative linear correlations, between the two variables, depending on their orientation. Triangular patterns, observed in certain parameter pairs, imply bounded relationships in which one parameter is constrained within a specific range relative to another. Band-like patterns, appearing as horizontal or vertical stripes, denote conditional relationships where one parameter remains relatively deterministic while the other adapts across different regions of the parameter space. U-shaped patterns, characterized by high parameter values at both low and high extremes of another variable but reduced values in the middle range, reflect nonlinear dependencies, indicating that the strength or direction of interaction between two parameters varies across their range. Similarly, dumbbell-like patterns, showing two distinct clusters separated by a sparse intermediate region, reveals the presence of two preferred parameter subspaces, potentially corresponding to different solutions with different property predictions. Collectively, these diverse correlation patterns highlight varying degrees of interdependence and constraint among parameter pairs, parameter–property pairs, and property pairs, offering valuable insights into the underlying physical and statistical relationships captured by the CG EAM framework.

The scatter patterns were plotted for the highest posterior density regions, representing the most probable solutions found for a given parameter during Bayesian sampling. The percentage coefficient of variation (%CoV) was calculated as the ratio of the posterior distribution’s standard deviation to its mean. Assuming Gaussian-like behavior, the 95% credible intervals were then determined by using the standard deviation and mean values of the posterior distributions. Tables S16–S23 in the ESI show this data for parameters for all five metals.

Before BUQ refinement, parameters were sampled from a uniform prior distribution in which all parameter values within the defined bounds were treated as equally probable. After BUQ, the posterior distributions for parameters also exhibited Gaussian-like unimodal distributions as well as bimodal distributions. A single Gaussian-like distribution indicates a single most probable range, while a bimodal distribution with distinct, well-separated peaks suggests the presence of two distinct local MAP probability values, each representing a local maximum solution. The existence of bimodal solutions is evidence of the complex relationship between the parameters and properties. Interestingly, a few parameters display a broader uniform-like distribution, suggesting that they are more flexible or less influential in the region of interest, indicating weaker sensitivity to the modeled observables.

As with parameters, the properties also exhibited diverse distribution patterns within the high-probability density regions of the posterior; such as Gaussian-like unimodal and bimodal distributions. Tables S24–S28 in the ESI present results for all properties across the five metals.

For Pd, Au, Cu, and Pt, the correlations between parameters and properties showed moderate Pearson coefficients. In contrast, Ag displayed either strong positive or negative correlations, consistent with the distinct clustering and bimodal posterior distributions observed for this metal. This suggests that Ag possesses a comparatively rougher potential energy surface, characterized by multiple competing local minima in parameter space relative to the other metals.

#### 3.5.2 Coverage Probabilities for the Posterior Distributions

The coverage probability is a powerful metric for assessing model accuracy, as it represents the posterior-estimated probability that the true value lies within a predetermined confidence interval.^29^ Coverage probability plots illustrate the alignment between the posterior and likelihood distributions, quantifying how much of the posterior falls within the corresponding confidence bounds. If the likelihood is assumed to contain the true value, then in cases where the posterior distribution responses include the likelihood without any significant change, we termed the solution as a “True-Unchanged Model” (TU).^29^ For cases where the posterior distribution’s plotted points rapidly rise above the coverage plot’s unity line, this means that the posterior distribution not only falls within the likelihood but also tightens the estimates of where the true value lies, and this situation is thus called a “True-Tightened Model” (TT). In this work, a model is designated as True-Tightened if the posterior distribution tightens the property by >20% relative to the initial uncertainty from the likelihood, while remaining within the likelihood bounds.

In the context of MD simulations, the ultimate goal is to achieve a True-Tightened FF. Such a model is highly desirable, as it has the ability not only to produce accurate predictions in line with our physical expectations but also to elevate our understanding beyond what was previously known before parameter estimation. And last, a “True-Loosened model” (TL) corresponds to a broadened posterior distribution, where uncertainties are loosened due to the larger credible intervals, which suggests the model property’s true value has a greater chance to lie outside of the confidence intervals originally associated with the likelihood. By applying these concepts to BUQ for MD model refinement and using likelihoods from experimental and theoretical data, we are able to gain a greater understanding of the true value. In many cases, the Bayesian parameter estimation produced narrower credible intervals, thereby improving the confidence in the inferred true values.

Figure 7 presents the coverage probability plots for the properties of all five metals expressed in units of standard deviations (σ). For cases exhibiting bimodal posterior distributions, representing distinct solution modes, the data were split into two separate spaces, representing the separate solutions. For instance, for copper, two modes were observed as a function of the parameter α, with α < 18.0999 defining Mode 1 and α > 18.10001 defining Mode 2. Similarly, Mode 1 and Mode 2 corresponded to α < 18.3499 and α > 18.35001, respectively, for silver and to α < 20.49999 and α > 20.50001, respectively, for platinum.

**Figure 7:**
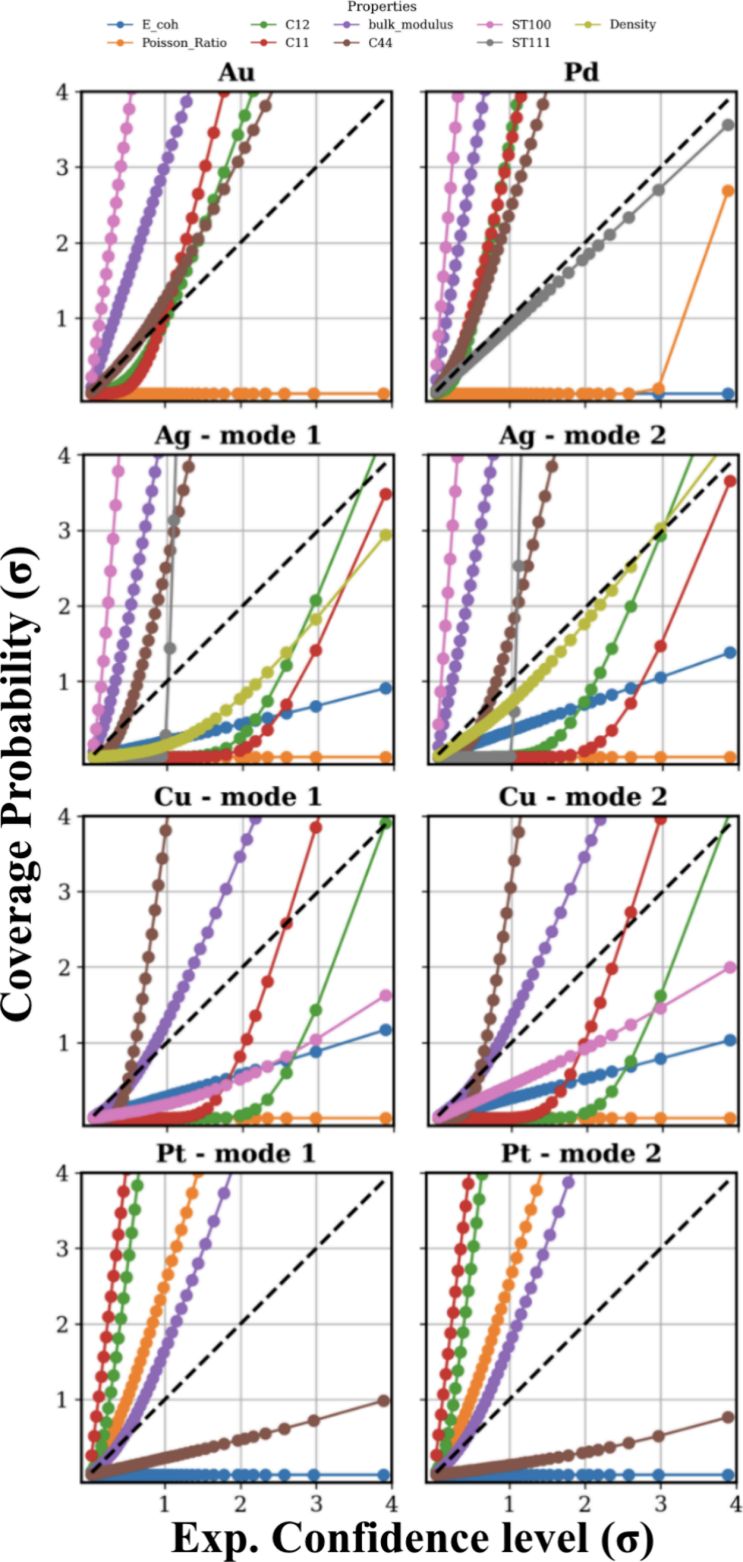
Coverage probabilities of all five metals comparing the true (posterior) distributions with the experimental likelihood. The horizontal axis represents the distance in confidence level probability space from the target value in terms of standard deviations (σ). The vertical axis represents the amount of the posterior contained within the region defined by the horizontal axis value and is presented in units of credible interval σ. Interpretation is provided in the text.

In Figure 7, property curves that rise sharply above the parity line correspond to tightened posteriors (narrower distribution relative to the likelihood), while those remaining below the line indicate loosened posteriors (wider distributions than the likelihood). Across all five metals, the bulk modulus exhibited TT behavior, while all models showed TT characteristics for C44. Both C11 and C12 were classified as TT for metals, except Ag and Cu. The Au model was TT for all the mechanical properties except Poisson’s ratio, density, and *E*_coh_, while the Pd model similarly exhibited TT behavior for all mechanical properties except Poisson’s ratio and *E*_coh_. Notably, Poisson’s ratio generally showed Loosened behavior across most metals.

For Ag and Pd, the density demonstrated TU behavior, while Au, Ag, and Pd displayed high coverage probabilities for surface properties, particularly for ST100. The Cu model exhibited TT behavior across both mechanical/elastic and structural properties, except C12 and Poisson’s ratio. Interestingly, late but sharp increases in coverage probabilities were observed for C11 and C12 in Cu and for C12 and the density in Ag, suggesting that these refined CG models remained narrowly within the 95% confidence bounds of their respective likelihoods. For density, all metals except Pd exhibited loosening after the refinement, indicating that the Bayesian framework allowed density to deviate slightly from the nominal target values in the pursuit of more physically consistent solutions.

Importantly, this process does not constitute naive parameter optimization: the properties were already optimized via PSO, whereas the Bayesian refinement explicitly incorporates uncertainties from both the model and the experimental data. This enables exploration of physically meaningful parameter spaces and the identification of solutions that better reconcile model predictions with experimental uncertainty, rather than simply minimizing residuals.^28^ Consequently, the TT CG EAM models represent not just numerically optimized solutions but also statistically and physical uncertainty informed representations of metallic behavior across scales.

### 3.6 Robust Credible Intervals of Parameters

During model development and fine-tuning, it is critical to determine the ranges over which model parameters can be varied while maintaining predictive accuracy. Having obtained the Bayesian-refined parameter sets, we conducted an additional analysis to identify robust ranges for each CG EAM model. Here, a robust range is defined as the interval within which each parameter can be varied while still reproducing selected material properties within their 95% confidence intervals. The 95% confidence limits were approximated by using two standard deviations from the experimental target mean values for each property of interest, a simplification that does not explicitly account for the correlation structure among properties. Quantitatively assessing parameter robustness helps identify which parameters can be safely tuned and to what extent to achieve accurate, stable, reliable, and interpretable models.

For this analysis, we focused only on those properties where the model achieved ∼100% coverage probability (Figure 7). Specifically, we selected the following: for Au and Pd, C11, C12, C44, Bulk Modulus, and Density; for Ag, C44, Bulk Modulus, ST100, and ST111; for Cu, C11, C44, and Bulk Modulus; and for Pt, C11, C12, C44, and Bulk Modulus. For each metal, robust parameter ranges were obtained by randomly sampling the parameter space near the MAP parameter set. The sampling radius for each parameter was gradually reduced until all of the properties were within the 95% confidence intervals. Each sampling involved 10,000 × *p* samples, where *p* is the number of parameters (here, *p* = 14 parameters, so total 140,000 samples per metal).

It is important to note that these robust ranges were derived from finite sampling in a high dimensional parameter space and are therefore necessarily incomplete. The reported ranges represent conservative (narrower) estimates of the true robust region. Nonetheless, these parameter ranges define practically useful intervals within which model parameters can vary while maintaining high prediction accuracy, reflecting the enhanced physical realism achieved through Bayesian refinement. The robust parameter ranges and their corresponding property ranges (including the experimental targets) for all five metals are presented in Tables 1–Table 5.

**Table 1:** Permissible Ranges of Perturbed Parameters for Pd to Target Having All Predicted Properties Remaining within the 95% Confidence Intervals of the Experimental Targets of Pd

Pd Parameters			
	MAP	Min	Max
ρ_e_	15.078	15.002	15.153
α	22.457	22.344	22.569
β	3.898	3.879	3.918
*A*	0.266	0.253	0.28
*B*	2.732	2.719	2.746
λ	1.409	1.339	1.48
κ	1.196	1.136	1.255
*F*_n3_	–2.077	–1.973	–2.181
*F*_2_	0.657	0.624	0.69
*F*_e_	–17.221	–17.135	–17.307
η	1.365	1.359	1.372
ρ_m_	0.757	0.719	0.795
Lattice constant	7.722	7.706	7.737
*f*_e_	1.554	1.546	1.562

Pd Properties			
	MAP Predicted	Min	Max
cohesive	–31.154	–31.122	–30.772
Poisson’s ratio	0.418	0.414	0.421
C12	171.826	166.047	176.435
C11	239.436	229.259	248.475
Bulk Modulus	194.363	187.27	200.442
C44	67.611	63.357	72.554
ST100	2055.194	2012.112	2109.352
Density	12.036	11.81	12.108

**2:** Permissible Ranges of Perturbed Parameters for Au to Target Having All Predicted Properties Remaining within the 95% Confidence Intervals of Experimental Targets of Au

Au Parameters			
	MAP	Min	Max
ρ_e_	16.29	16.257	16.323
α	16.809	16.775	16.843
β	4.951	4.941	4.961
*A*	0.301	0.301	0.302
*B*	2.183	2.179	2.188
λ	1.416	1.345	1.487
κ	1.174	1.115	1.232
*F*_n3_	–1.867	–1.773	–1.96
*F*_2_	1.798	1.708	1.888
*F*_e_	–17.711	–17.676	–17.747
η	1.229	1.226	1.231
ρ_m_	0.667	0.634	0.7
Lattice constant	8.235	8.234	8.235
*f*_e_	1.541	1.464	1.618

Au Properties			
	MAP Predicted	Min	Max
cohesive	–28.805	–28.473	–28.362
Poisson’s ratio	0.44	0.441	0.443
C12	153.415	157.331	160.481
C11	195.435	195.55	199.41
Bulk Modulus	167.422	171.392	174.58
C44	42.02	41.301	42.567
ST100	1519.253	1523.592	1559.066
Density	19.334	19.222	19.295

**3:** Permissible Ranges of Perturbed Parameters for Both Modes of Ag to Target Having All Predicted Properties Remaining within the 95% Confidence Intervals of Experimental Targets of Ag

Ag Parameters						
	Mode 1			Mode 2		
	MAP	Min	Max	MAP	Min	Max
ρ_e_	15.016	14.986	15.046	15.382	15.351	15.413
α	18.589	18.552	18.627	18.062	18.026	18.098
β	4.645	4.636	4.654	4.753	4.743	4.762
*A*	0.347	0.34	0.354	0.342	0.335	0.349
*B*	2.089	2.047	2.13	2.169	2.126	2.213
λ	1.28	1.255	1.306	1.31	1.283	1.336
κ	1.225	1.2	1.249	1.216	1.192	1.241
*F*_n3_	–1.679	–1.645	–1.712	–1.621	–1.589	–1.654
*F*_2_	2.668	2.663	2.673	2.512	2.507	2.517
*F*_e_	–14.516	–14.487	–14.545	–13.869	–13.841	–13.897
η	0.907	0.905	0.909	0.907	0.905	0.909
ρ_m_	0.822	0.82	0.823	0.818	0.816	0.82
Lattice constant	7.97	7.955	7.986	8.163	8.147	8.18
*f*_e_	1.862	1.858	1.866	1.88	1.876	1.884

Ag Properties						
	Mode 1			Mode 2		
	MAP Predicted	Min	Max	MAP Predicted	Min	Max
cohesive	–23.789	–24.136	–23.542	–23.845	–24.106	–23.458
Poisson’s ratio	0.401	0.4	0.404	0.403	0.399	0.403
C12	88.266	86.758	91.769	89.846	85.377	90.691
C11	131.847	127.794	136.705	132.88	127.08	136.489
Bulk Modulus	102.793	100.502	106.783	104.191	99.286	105.927
C44	43.581	40.911	45.234	43.034	41.278	45.714
ST100	1247.773	1204.891	1283.679	1250.247	1209.658	1292.816
ST111	987.39	1033.836	1048.55	1030.17	1037.425	1052.073
Density	10.197	10.545	10.79	10.676	10.058	10.314

**4:** Permissible Ranges of Perturbed Parameters for Both Modes of Cu to Target Having All Predicted Properties Remaining within the 95% Confidence Intervals of Experimental Targets of Properties of Cu

Cu Parameters						
	Mode 1			Mode 2		
	MAP	Min	Max	MAP	Min	Max
ρ_e_	13.15	13.146	13.154	13.153	13.149	13.157
α	18.748	18.743	18.754	17.32	17.315	17.326
β	4.446	4.445	4.448	4.449	4.448	4.451
*A*	0.371	0.357	0.386	0.373	0.358	0.388
*B*	2.009	2.008	2.009	2.007	2.006	2.008
λ	1.067	1.025	1.11	1.066	1.023	1.108
κ	1.245	1.195	1.295	1.307	1.255	1.36
*F*_n3_	–1.287	–1.236	–1.339	–1.329	–1.276	–1.382
*F*_2_	0.779	0.748	0.81	0.741	0.712	0.771
*F*_e_	–19.111	–19.105	–19.116	–19.086	–19.08	–19.092
η	0.738	0.738	0.738	0.734	0.734	0.734
ρ_m_	0.715	0.686	0.743	0.713	0.684	0.741
Lattice constant	7.14	7.139	7.141	6.93	6.929	6.932
*f*_e_	1.598	1.598	1.599	1.607	1.606	1.607

Cu Properties						
	Mode 1			Mode 2		
	MAP Predicted	Min	Max	MAP Predicted	Min	Max
cohesive	–28.117	–28.267	–28.177	–28.541	–28.214	–28.12
Poisson’s ratio	0.386	0.384	0.385	–1.345	0.387	0.388
C12	112.111	111.918	113.5	114177.202	113.206	114.868
C11	178.245	179.059	180.969	–204013.67	178.898	180.917
Bulk Modulus	134.156	134.33	136.027	10222.066	135.143	136.933
C44	66.134	67.322	67.695	68.439	65.947	66.378
Density	8.848	8.755	8.783	9.379	9.096	9.122

**Table 5:** Permissible Ranges of Perturbed Parameters for Both Modes of Pt to Target Having All Predicted Properties Remaining within the 95% Confidence Intervals of Experimental Targets of Properties of Pt

Pt Parameters						
	Mode 1			Mode 2		
	MAP	Min	Max	MAP	Min	Max
ρ_e_	19.584	19.525	19.643	19.549	19.491	19.608
α	19.709	19.649	19.768	21.302	21.238	21.366
β	6.257	6.239	6.276	6.144	6.126	6.163
*A*	0.417	0.416	0.418	0.412	0.411	0.414
*B*	3.092	3.083	3.102	3.107	3.098	3.116
λ	1.642	1.56	1.725	1.646	1.564	1.729
κ	1.851	1.846	1.857	1.875	1.87	1.881
*F*_n3_	–0.76	–0.722	–0.798	–0.759	–0.721	–0.796
*F*_2_	1.756	1.669	1.844	1.649	1.567	1.732
*F*_e_	–27.284	–27.202	–27.366	–27.296	–27.214	–27.378
η	0.941	0.938	0.944	0.961	0.958	0.964
ρ_m_	0.842	0.799	0.884	0.791	0.752	0.831
Lattice constant	8.113	8.112	8.114	8.064	8.024	8.104
*f*_e_	1.706	1.701	1.712	1.706	1.701	1.711

Pt Properties						
	Mode 1			Mode 2		
	MAP Predicted	Min	Max	MAP Predicted	Min	Max
cohesive	–44.839	–43.934	–43.654	–44.877	–44.018	–43.706
C12	256.619	246.321	262.797	257.786	243.559	266.139
C11	342.541	323.695	353.362	342.658	318.078	354.895
Bulk Modulus	285.26	271.474	291.468	286.076	268.579	294.424
C44	85.922	77.383	94.817	84.872	74.734	93.718
Density	21.975	22.501	23.175	22.214	21.805	23.017

As expected, the MAP parameter values consistently fall within the identified robust ranges, although the MAP-predicted property values occasionally lie outside these intervals due to the limitations of finite sampling. This highlights the inherent complexity of the high-dimensional parameter landscape, which is nonmonotonic and non-Gaussian. Consequently, selecting an arbitrary single parameter set from within the robust range may not yield the optimal model for a specific application.

For applications that do not involve additional fine-tuning, the MAP parameter set should be used directly, whereas the robust ranges provide a statistically informed parameter space for targeted refinement. Practitioners seeking fine-tuned solutions should conduct extensive sampling within these robust ranges (such as 10,000 × *p* samples, where *p* is the number of parameters) or employ objective function optimization to determine parameter sets that best meet the performance criteria of interest. The key value added from the robust range is that it provides a region for sampling that has an enhanced probability of finding physically realistic and target property producing solutions. This achieves the goal that was sought in this study.

## 4 Conclusions

Here, we present the first coarse-grained Embedded Atom Method (CG EAM) interatomic potentials developed for five face-centered cubic (FCC) metals: palladium (Pd), gold (Au), silver (Ag), copper (Cu), and platinum (Pt). Each metal was represented by using an 8:1 mapping scheme that preserves the FCC lattice symmetry. Initially, the CG EAM potential (or force field, FF) parameters were optimized using the Particle Swarm Optimization (PSO) method to reproduce key physical, mechanical, and thermodynamic properties, achieving agreement within 5% of the literature-reported experimental or density functional theory (DFT) values.

The model parameters were subsequently refined using a Bayesian Uncertainty Quantification (BUQ) framework to perform uncertainty quantification (UQ) for both parameters and predicted properties. For all five metals, the BUQ framework developed here yields the most probable parameter sets while incorporating the experimental uncertainties for the target properties. The BUQ framework also provides credible intervals for each parameter, quantifies uncertainties in output properties, and identifies the sensitivity and influence of embedding-energy and pair-potential terms within the EAM formalism, particularly in relation to the metals’ free-electron clusters.

Importantly, the BUQ framework enables the extraction of robust parameter ranges by leveraging coverage probability plots that account for the confidence intervals surrounding the target property values. This approach establishes a quantitative pathway for parameter tuning and model refinement, demonstrating how property-level confidence intervals can inform the physical consistency of parameter ranges.

The refined CG EAM models are envisioned to facilitate the study of bulk metallic behavior as well as molecular dynamics (MD) simulations of self-assembly in ligand-grafted metal nanoparticles. More broadly, the methodology developed here provides a generalizable strategy for parameter optimization, uncertainty quantification, and robustness assessment, advancing molecular-level mechanistic understanding and accelerating the data-driven design of metallic and hybrid materials across diverse application domains.

## Supplementary Material



## Data Availability

The EAM potential files generated for all CG metals developed using PSO and refined using BUQ are available on our GitHub: https://github.com/Deshmukh-Group/CG-EAM-Metals-BUQ/tree/main/CG-EAM-BUQ-Github.

## Notes

Detailed methodology of Particle Swarm Optimization (PSO) and Embedded Atom Method (EAM) potentials for modeling metals. Results: Convergence of ESS sampler and posterior distribution corner plots for all parameters and properties, credible intervals from posterior distributions, and MD validation of BUQ most probable sets. (PDF)
