# Supplementary material for: Development and Bayesian Uncertainty Quantification of Coarse-Grained Models of Metals Based on Embedded Atom Method Potentials
Source: J Chem Theory Comput. 2025 Dec 5;21(24):12481–96. doi: 10.1021/acs.jctc.5c01322 (PMC12746438; doi:10.1021/acs.jctc.5c01322)
Supplement: Supplementary file 1 [file ct5c01322_si_001.pdf]

Electronic Supporting Information

Development and Bayesian Uncertainty Quantification of Coarse-Grained Models of Metals Based on Embedded Atom Method Potentials

Abhishek T. Sose[1], Troy Gustke[1], Karteek K. Bejagam[1], Fangxi Wang[1], Aditya Savara[2]* and Sanket A. Deshmukh[1]*

[1]Department of Chemical Engineering, Virginia Tech, Blacksburg, VA, 24060, USA

[2]Oak Ridge National Laboratory, Oak Ridge, Tennessee 37830, United States

*Corresponding Authors: Dr. Sanket A. Deshmukh (sanketad@vt.edu)

Dr. Aditya Savara (savaraa@ornl.gov)

Section S1: Methodology:

S1.1 Mapping Scheme of CG Metals

Table S1 shows the mass of CG beads for the five metals, namely, palladium (Pd), gold (Au), silver (Ag), copper (Cu), and platinum (Pt).

Table S1. Mass of each CG bead for all five metals.

Metal Name	Mass of atom (in Da)	Mass of CG bead (this work) (in Da)
Palladium (Pd)	106.42	851.36
Gold (Au)	196.97	1575.76
Silver (Ag)	107.8682	862.9456
Copper (Cu)	63.546	508.368
Platinum (Pt)	195.084	1560.672

S1.2 Coarse-Grained Embedded Atom Method (EAM) Potentials

In molecular dynamics (MD) simulations, the Embedded Atom Method (EAM) potential is widely used due to its high accuracy, which stems from its quantum mechanics-based simple mathematical formalism.[1] EAM is employed in large-scale computer simulations because it efficiently calculates the total energy of a system as stated in the following expression:

$$E_{tot}=\sum_{i}^{N}F_i(\rho_i)+\sum_{i,j>i}^{N}\phi_{ij}(R_{ij})$$ ……. **Eq. (S1)**

In this equation, $F_i(\rho_i)$ represents the embedding energy, which is the energy required to embed a CG bead *i* into the local electronic density cloud ρ_i created by surrounding CG beads. The term $\phi_{ij}(R_{ij})$ describes the pairwise interaction energy between beads *i* and *j* at a distance R_{ij}. The local density ρ_i is defined as:

$$\rho_i = \sum_{j \neq i} f_j(R_{ij})$$ **…….. Eq. (S2)**

The functional form of the individual atomic contributions f_j to the host electron density is of the same form as the attractive part of the pairwise interaction, with the same values of β and λ:

$$f_j = \frac{f_e \exp\left\{-\beta\left[\left(R_{ij}/r_e\right)-1\right]\right\}}{1+\left[\left(R_{ij}/r_e\right)-\lambda\right]^{20}}$$ **…….. Eq. (S3)**

Here, f_j is the electron density contributed by bead j, which is located at a distance R_{ij} from bead i. The pairwise interaction potential $\phi_{ij}(R_{ij})$ is given by:

Φ_{ij} = [illegible] - [illegible] **…….. Eq. (S4)**

In this expression, r_e represents the equilibrium distance between nearest-neighbor beads and it was calculated as lattice constant $L_c/\sqrt{2}$ while A, B, α, and β are adjustable parameters. The terms κ and λ are cut-off parameters that control the distance at which the interaction between two beads becomes negligible.

To model the embedding energy over a broad range of densities, Johnson et al. proposed an approach that divides the density into regions with distinct functional forms.[2] For electron densities within the range [0, 0.85ρ_e] and [0.85ρ_e, 1.15ρ_e], cubic spline functions with different coefficients are used. Here, ρ_e represents the equilibrium electron density, where the embedding energy reaches its minimum. In regions where the density exceeds 1.15ρ_e, a repulsive embedding energy is modeled using an asymptotic power-logarithmic functional form to handle high-density scenarios. While this EAM functional form accurately describes bulk systems and surfaces, it may not be precise for low-density regions, such as beads located at the edges or corners of

nanoparticles. To address this, Shan et al. introduced another cubic spline to model low-density regions, specifically in the range [0, ρ_l] where ρ_l is an adjustable parameter.[3]

$$F_i(\rho_i)=\begin{cases}\sum_{k=0}^{3} F_{mk}\left(\frac{\rho_i}{\rho_l}-1\right)^k & \text{for } 0\le \rho_i<\rho_l;\ \rho_l=\gamma\rho_e\\ \sum_{k=0}^{3} F_{nk}\left(\frac{\rho_i}{\rho_n}-1\right)^k & \text{for } \rho_l\le \rho_i<\rho_n;\ \rho_n=0.85\rho_e\\ \sum_{k=0}^{3} F_{k}\left(\frac{\rho_i}{\rho_e}-1\right)^k & \text{for } \rho_n\le \rho_i<\rho_0;\ \rho_0=1.15\rho_e\\ F_e\left[1-\eta\ln\left(\frac{\rho_i}{\rho_e}\right)\right]\left(\frac{\rho_i}{\rho_e}\right)^\eta & \text{for } \rho_i\ge\rho_0\end{cases}$$

…….. Eq. (S5)

S1.3 Property Calculations

The methodology of calculations and target property values are mentioned in the following section.

***(i)* Cohesive energy**: The cohesive energy is the energy required to separate constituent atoms apart from each other from the state of assembly. In this case, the separated CG atom is found here to be composed of 8 AA atoms still assembled together. Therefore, the cohesive energy per bead of the CG model should be eight times the cohesive energy per atom in the all-atom (AA) model. In this case, potential energy per atom is calculated as per following commands in lammps:

"compute eng all pe/atom
compute eatoms all reduce sum c_eng"

The total energy of assembly (eatoms) is divided by the total number of atoms (n_atoms) to compute the cohesive energy. Note, for these minimization steps for cohesive energy calculations during the PSO optimization we performed only 4 CG beads calculations.

***(ii)* Density:** In this study, the cell parameter i.e. lattice constant was allowed to vary to achieve equilibrium density through NPT molecular dynamics simulation under standard conditions (1 bar and 298.15 K). A Nose-Hoover thermostat with 1.0 ps and barostat with 20.0 ps coupling constants, with a timestep of 1 fs was run to equilibrate the system within a total of 50 ps. Note,

while the PSO optimization was performed on a system with 1000 CG beads, the final posterior validation run in the BUQ analysis utilized a system with 8000 CG beads. Additionally, to showcase the proficiency of CG modeling, we ran the density calculations with a timestep of 5 fs, 10 fs, and 20 fs.

***(iii)* Surface Tension:** The models of {100} and {111} surfaces were constructed with the dimensions l_x, l_y: 8 x L_c, 8 x L_c (L_c: Lattice constant). While in the z-direction, the box length spanned to 16 x L_c, whereas atoms consisted only in length, 12 x L_c. The free space (vacuum slab) generated by removing atoms in the length of 4 x L_c, was sufficient to estimate the surface energy of the two separated surfaces. The total energy of the separated system after minimization was computed as E_s. And the energy of the unified surfaces, E_u, was determined by the cohesive energy times the total number of atoms. The surface tension was calculated using:

$$\gamma \approx \frac{E_s - E_u}{2A} \text{............} \textbf{(Eq. S6)}$$

Where, the denominator comprises total surface area (from both surfaces), *i.e.*, 2 x A = 2 x l_x x l_y. The surface tensions for 100 and 111 surfaces were only considered because of the availability of reliable experimental data to optimize for. Note, for surface tension calculations we used a system size of 3200 CG beads throughout the PSO optimization and validation run.

***(iv)* Elastic constants:** Isotropic elastic constants, including Poisson's ratio, C11, C12, shear modulus (C44), and bulk modulus, were calculated based on Hooke's law. The system was displaced in one direction (x, y, or z) to measure the resulting stress and strain in all three directions. From the stress-strain tensor, the stiffness constants C11, C12, and C44 were computed. Using these constants, the bulk modulus and Poisson's ratio were determined with the following equations:

$$\text{Bulk modulus} = \frac{\text{[illegible]}}{9} \text{........} \textbf{Eq. (S7)}$$

$$\text{Poisson Ratio} = \frac{\text{[illegible]}}{2\text{[illegible]}} \text{........} \textbf{Eq. (S8)}$$

Note, while the PSO optimization was performed on a system with 96 CG beads, the final posterior validation run in the BUQ analysis utilized a system with 6144 CG beads.

S1.4 Particle Swarm Optimization (PSO):

The Particle Swarm Optimization (PSO) algorithm, initially introduced by Kennedy and Eberhart in the 1970s, is inspired by the collective behavior of bird flocks during their search for food. This method operates through an iterative process aimed at improving the positions of a group of candidate solutions, referred to as particles, within a defined search space. Each particle represents a unique set of parameters or variables, and the algorithm typically employs a large number of particles to explore a broader parameter space, thereby accelerating the identification of optimal solutions. However, to prevent excessive computational demands, it is crucial to balance the number of particles used, neither too many nor too few.

At the start of the optimization process, each particle is initialized with random positions (representing the parameters to be optimized) and velocities. The fitness of a particle is evaluated based on how closely its simulated properties align with the desired target properties, as determined through molecular dynamics simulations. During the optimization, each particle tracks its personal best position (p_{best}), which corresponds to the location where it achieved its highest fitness up to that point. Additionally, the global best position (g_{best}) is identified as the best fitness value achieved by any particle across all completed iterations of the PSO algorithm. The movement of particles is governed by the following equations, which update their velocities and positions iteratively:

$$V_{n+1} = w*V_n + c1*rand()*(g_{best} - x_n) + c2*rand()*(p_{best} - x_n) \text{ …….. } \mathbf{Eq.\ (S9)}$$

$$x_{n+1} = x_n + V_{n+1} * \Delta t \text{ …….. } \mathbf{Eq.\ (S10)}$$

Here, V_{n+1} and x_{n+1} represent the updated velocity and position of the particle, respectively. The constants c1 and c2 determine the influence of the global best (g_{best}) and personal best (p_{best}) positions on the particle's new velocity. The inertia factor w is introduced to incorporate momentum from the particle's previous velocity, helping to avoid premature convergence to suboptimal solutions.

The PSO algorithm iteratively updates the positions and velocities of all particles, as well as the p_{best} and g_{best} values, until at least one particle achieves a fitness value below a predefined tolerance threshold. This signifies the identification of an optimal set of input parameters. In this

study, the PSO algorithm was implemented using 64 particles to explore a 14-dimensional parameter space, with the bounds for each parameter specified in **Table S2-S6**. A total of 100 PSO epochs were executed to achieve the desired target properties.

S1.5 MD Simulations Details:

CG MD simulations were used to generate data to train Gaussian Process Regression (GPR) models, and to validate the final parameters obtained from Bayesian optimization. The first step for CG EAM model validation was to calculate the cohesive energy of the system. This cohesive energy was used in subsequent calculations wherever required. The MD simulations for density were run for 50 ps with a timestep of 1 fs to ensure that most simulations during parameter optimization were uninterrupted. All the MD simulations were run using Large-scale Atomic/Molecular Massively Parallel Simulator (LAMMPS) software package to calculate the property values.[4] Note, all the system sizes used to calculate individual properties are provided in **Section S1.3** and except for surface tension calculations all other systems were periodic in all three dimensions. Cohesive energy and elastic constants were calculated by minimizing/relaxing the system with 20,000 maximum iterations and 200,000 maximum evaluations with stopping criteria for both energy and force at $1*10^{25}$. While in the case of surface tension/energy, these numbers were {max_iterations: 10,000; max_evaluations: 100,000, with stopping criteria for both energy and force at $1*10^{15}$}. Furthermore, we ran simulations for density calculations only at 298.15 K, which was controlled using Nose-Hoover thermostat with 1.0 ps and barostat with 20.0 ps coupling constants, with a timestep of 1 fs was run to equilibrate the system within a total of 50 ps.

To train GPR models, 5000 sets of CG EAM parameters were generated using Sobol sequence. For each of these parameter sets MD simulations were performed and resulting output was used to train these GPR models.

Section S2 Results and Discussion:

S2.1 PSO-optimized Parameters and Predicted Properties:

Tables S2 to **S5** report the ranges used during optimization of all 14 parameters along with the PSO-optimized parameters that reproduced the target properties with acceptable errors. These properties along with the target properties are listed in Tables S7 and S8 for all five metals.

Table S2. The table of range of values used by PSO for all 14 parameters with the PSO-optimized set of CG Pd. A comparison of properties predicted by CG models using PSO-optimized parameter sets against experimental target values, along with their corresponding errors is also presented.

Pd			
	**Min**	**Max**	**PSO-optimized Set**
ρ_e	14.903	15.146	14.96
α	22.552	22.75	22.581
β	3.95	4.15	3.963
A	0.25	0.28	0.278
B	2.602	2.75	2.71
λ	1.33	1.39	1.363
κ	1.19	1.22	1.193
F_{n3}	-2.08	-2.05	-2.08
F_2	0.65	0.8	0.66
F_e	-17	-16.002	-16.652
η	1.31	1.35	1.324
ρ_m	0.9	0.95	0.769
Lattice constant	7.71	7.73	7.711
f_e	1.45	1.65	1.555
Properties			
	**Target Properties**	**PSO-optimized Set Predicted Properties**	**Error (%)**
Cohesive energy (eV)	-31.28[5]	-31.03	0.80
Poisson Ratio	0.374	0.41	9.63
C12 (GPa)	176[6]	170.62	3.06
C11 (GPa)	234.1[6]	241.06	2.97
C44 (GPa)	71.2[6]	70.44	1.07
Bulk Modulus (GPa)	193[7]	194.1	0.57
Surface Tension 100 (mN/m)	2059.2[8,9]	2158.54	4.82
Surface	-	-	-

Tension 110 (mN/m)			
Surface Tension 111 (mN/m)	1980[8,9]	1974.71	0.26
Density (gm/cc)	12.023[10]	12.027	0.03

Table S3. The table of range of values used by PSO for all 14 parameters with the PSO-optimized set of CG Au. A comparison of properties predicted by CG models using PSO-optimized parameter sets against experimental target values, along with their corresponding errors is also presented.

**Au**			
**Parameters**			
	**Min**	**Max**	**PSO-optimized Set**
ρ_e	13.598	21.266	16.9456
α	14.378	29.498	17.5048
β	4.601	7.443	4.957
A	0.15	0.35	0.3134
B	0.916	2.5	2.1078
λ	0.504	1.85	1.4302
κ	0.688	1.998	1.1534
F_{n3}	-2.189	-0.522	-1.7975
F_2	0.523	2.86	1.8437
F_e	-23.354	-14.024	-17.0336
η	0.812	1.8	1.209
ρ_m	0.266	0.95	0.679
Lattice constant	8.122	8.16	8.16
f_e	1.55	1.56	1.5547
**Properties**			
	**Target Properties**	**PSO-optimized Set Predicted Properties**	**Error (%)**
Cohesive energy (eV)	-30.48[11]	-27.80	8.79
Poisson Ratio	0.42[12,13]	0.427	1.67
C12 (GPa)	163[14]	155.67	4.49

C11 (GPa)	192[14]	208.40	8.54
C44 (GPa)	42.3[14]	52.73	24.66
Bulk Modulus (GPa)	173[15]	173.25	0.14
Surface Tension 100 (mN/m)	1540[9]	1538.78	0.08
Surface Tension 110 (mN/m)	1600[9]	1613.85	-
Surface Tension 111 (mN/m)	1480[9]	1474.69	0.36
Density (gm/cc)	19.3[16,17]	19.89	3.06

Table S4: The table of range of values used by PSO for all 14 parameters with the PSO-optimized set of CG Ag. A comparison of properties predicted by CG models using PSO-optimized parameter sets against experimental target values, along with their corresponding errors is also presented.

**Ag**			
	**Min**	**Max**	**PSO-optimized Set**
ρ_e	14.515	15.7	15.17
α	17.513	19	18.331
β	4.5	5.2	4.783
A	0.32	0.36	0.353
B	1.902	2.3	2.112
λ	1.251	1.45	1.32
κ	1.155	1.35	1.253
F_{n3}	-1.7	-1.501	-1.679
F_2	2.45	2.85	2.573
F_e	-15.5	-13.006	-14.221
η	0.703	0.95	0.873
ρ_m	0.7	0.85	0.792
Lattice constant	8.1	8.13	8.127
f_e	1.701	2	1.844
Properties			
	**Target**	**PSO-optimized Set**	**Error (%)**

		Predicted Properties	
Cohesive energy (eV)	-23.78[18]	-23.54	0.99
Poisson Ratio	0.337[8,19,20]	0.397	17.8
C12 (GPa)	93.67[7]	88.68	5.32
C11 (GPa)	123.99[7]	134.90	8.79
C44 (GPa)	46.12[7]	46.22	0.21
Bulk Modulus (GPa)	104[7]	104.09	0.08
Surface Tension 100 (mN/m)	1237.6[8,9]	1232.53	0.41
Surface Tension 110 (mN/m)	-	-	-
Surface Tension 111 (mN/m)	1190[8,9]	1185.60	0.37
Density (gm/cc)	10.49[21]	10.36	1.24

Table S5: The table of range of values used by PSO for all 14 parameters with the PSO-optimized set of CG Cu. A comparison of properties predicted by CG models using PSO-optimized parameter sets against experimental target values, along with their corresponding errors is also presented.

Cu			
	**Min**	**Max**	**PSO-optimized Set**
ρ_e	13.2	15	13.684
α	18	20.5	18.035
β	3.8	4.4	4.286
A	0.35	0.55	0.363
B	2.05	2.5	2.087
λ	0.8	1.15	1.073
κ	1.2	1.55	1.292

F_{n3}	-1.4	-1.2	-1.279
F_2	0.65	1.05	0.75
F_e	-18.8	-14	-18.399
η	0.3	0.8	0.71
ρ_m	0.45	0.8	0.693
Lattice constant	7.175	7.21	7.2
f_e	1.3	2.4	1.555
Properties			
	**Target**	**PSO-optimized Set Predicted Properties**	**Error (%)**
Cohesive energy (eV)	-28.19[18]	-28.23	0.13
Poisson Ratio	0.323[22]	0.37	15.81
C12 (GPa)	121.4[23]	111.85	7.86
C11 (GPa)	168.4[23]	187.18	11.15
C44 (GPa)	75.4[23]	75.32	0.1
Bulk Modulus (GPa)	137[7,23]	136.96	0.03
Surface Tension 100 (mN/m)	1840.8[8,9]	1815.87	1.35
Surface Tension 111 (mN/m)	1770[8,9]	1825.49	3.13
Density (gm/cc)	8.96[24]	8.93	0.29

Table S6: The table of range of values used by PSO for all 14 parameters with the PSO-optimized set of CG Pt. A comparison of properties predicted by CG models using PSO-optimized parameter sets against experimental target values, along with their corresponding errors is also presented.

**Pt**			
	**Min**	**Max**	**PSO-optimized Set**
ρ_e	18.7	22	18.845

α	19.5	23.5	20.509
β	4.8	7.5	6.063
A	0.35	0.55	0.429
B	1.95	3.05	2.989
λ	1.65	2.55	1.673
κ	1.65	2.65	1.877
F_{n3}	-1	-0.65	-0.756
F_2	1.65	2.5	1.703
F_e	-29.5	-20.5	-26.266
η	0.95	1	0.972
ρ_m	0.75	0.85	0.821
Lattice constant	7.81	7.855	7.819
f_e	1.4	2.2	1.771
Properties			
	**Target**	**PSO-optimized Set Predicted Properties**	**Error (%)**
Cohesive energy (eV)	-46.72[25]	-42.23	9.62
Poisson Ratio	0.393[5,8]	0.43	10.89
C12 (GPa)	251[5]	257.27	2.5
C11 (GPa)	347[5]	333.08	4.01
C44 (GPa)	76[5]	75.82	0.24
Bulk Modulus (GPa)	283[7]	282.54	0.16
Surface Tension 100 (mN/m)	2558.4[8,9]	2567.05	0.34
Surface Tension 111 (mN/m)	2460[8,9]	2469.82	0.4
Density (gm/cc)	21.46[26]	23.54	9.71

S2.2 Comparison of Properties Predicted by PSO-optimized Parameters with Existing Models:

Table S7 to **S11** shows the comparison of PSO-predicted parameters with all-atom models reported in the literature.

Table S7: A comparison of the properties predicted by the PSO-optimized CG EAM potential with those predicted by the existing all-atom EAM potential for Pd. “-” is added because those properties were not reported by authors.

**Properties**	**Pd**										
	**Target**	**PSO-optimized Set Predicted Properties**	[27]	[28,29]	[30,31]	[32,33]	[31]	[33]	[34]	[28]	[35]
Cohesive energy (eV)	-31.28	-31.03	-31.28	-31.28	-31.283	-31.123	-31.079	-	-	-	-
ESI_FCC_metalsPoisson Ratio	0.374	0.41	-	-	-	-	-	-	-	-	-
C12 (GPa)	176	170.62	183.017	184.456	200.476	175.684	200.473	-	-	-	-
C11 (GPa)	234.1	241.06	221.062	218.188	246.656	249.471	246.654	-	-	-	-
C44 (GPa)	71.2	70.44	72.737	65.076	56.16	81.259	56.16	-	-	-	-
Bulk Modulus (GPa)	193	194.1	195.698	195.7	215.869	200.279	215.867	-	-	-	-
Surface Tension 100 (mN/m)	2059.2	2158.54	1451.95	-	-	-	-	1627.96	1734.6	1364.29	765.71
Surface Tension 111 (mN/m)	1980	1974.71	1300.52	-	-	-	-	1515.21	1433.69	1215.29	667.21
Density (gm/cc)	12.023	12.027	12.009	12.009	12.056	12.005	12.056	-	-	-	-

Table S8. A comparison of the properties predicted by the PSO-optimized CG EAM potential with those predicted by the existing all-atom EAM potential for Au. "-" is added because those properties were not reported by authors.

**Properties**	**Au**										
	**Target**	**PSO-optimized Set Predicted Properties**	[36]	[37]	[27]	[38]	[39]	[40]	[41]	[42]	[32,33]
Cohesive Energy	-30.48	-27.8	-30.824	-30.312	-31.44	-31.397	-31.394	-30.48	-31.44	-30.48	-31.44
Poisson's Ratio	0.42	0.427	0.464	0.457	0.464	0.457	0.457	0.457	0.45	0.433	0.452
C12	163	155.67	162.502	157.754	158.714	169.845	169.552	169.972	167.808	162.593	157.359
C11	192	208.4	188.073	187.537	183.249	202.157	201.674	201.633	205.419	213.035	190.722
C44	42.3	52.73	43.337	43.322	45.336	46.278	46.088	45.586	48.898	65.47	44.927
Bulk Modulus	173	173.25	171.026	167.682	166.892	180.615	180.259	180.525	180.345	179.407	168.48
ST100	1540	1538.78	1377.107	-	-	-	-	-	-	-	-
ST110	1600	1613.85	1524.321	-	-	-	-	-	-	-	-
ST111	1480	1474.69	1250.786	-	-	-	-	-	-	-	-
Density	19.3	19.89	19.018	19.29	19.26	19.42	19.4	19.26	19.29	19.47	19.26

Table S9. A comparison of the properties predicted by the PSO-optimized CG EAM potential with those predicted by the existing all-atom EAM potential for Ag. "-" is added because those properties were not reported by authors.

**Properties**	**Ag**											
	**Target**	**PSO-optimized Set Predicted Properties**	[30,31]	[37]	[37]	[27]	[28]	[43]	[44]	[45]	[32,33]	

Cohesive energy (eV)	-23.78	-23.54	-22.8	-23.74	-23.74	-22.8	-22.8	-23.771	-22.8	-22.626	-22.8	-22.8
Poisson Ratio	0.337	0.397	0.43	0.429	0.429	0.414	0.413	0.433	0.43	0.427	0.417	0.418
C12 (GPa)	93.67	88.68	93.875	94.028	94.028	91.075	91.043	94.162	93.875	90.62	93.772	93.49
C11 (GPa)	123.99	134.9	124.243	125.119	125.119	129.07	129.137	123.438	124.243	121.422	131.174	130.409
C44 (GPa)	46.12	46.22	46.551	47.194	47.194	56.802	56.845	47.153	46.553	47.149	50.529	50.518
Density (gm/cc)	10.49	10.36	10.471	10.501	10.501	10.471	10.471	9.947	10.471	10.47	10.473	10.473

**Properties**	**Ag**													
	**Target**	**This work**	46	32,33	35	27	37	28						
Bulk Modulus (GPa)	104	-	-	-	-	-	-	-	104.449	115.341	103.851	103.425	103.71	95.178	95.378
Surface Tension 100 (mN/m)	1237.6	1043.77	977.74	977.73	614.09	703.27	759.56	701.84	-	-	-	-	-	-
Surface Tension 111 (mN/m)	1190	906.72	906	905.99	546.82	618.45	621.03	617.06	-	-	-	-	-	-

Table S10. A comparison of the properties predicted by the PSO-optimized CG EAM potential with those predicted by the existing all-atom EAM potential for Cu. “-” is added because those properties were not reported by authors.

	**Target**	**This work**	32	47	48	49	50	51	52	53	33	54	55	56
Cohesive	-28.19	-28.2	28.318	28.155	28.32	28.32	28.32	27.382	26.265	28.32	28.32	28.32	-	-

Energy		3												
Poisson's Ratio	0.323	0.37	-	-	-	-	-	-	-	-	-	-	-	-
C12	121.4	111.85	117.109	122.099	123.755	124.154	123.468	127.194	127.81	122.651	122.598	115.325	-	-
C11	168.4	187.18	180.411	169.863	168.063	167.265	168.642	174.137	174.458	169.928	198.206	184.173	-	-
C44	75.4	75.32	70.994	76.621	79.052	76.644	76.762	83.853	84.54	76.408	92.61	68.852	-	-
Bulk Modulus	137	136.96	-	-	-	-	-	-	-	-	-	-	-	-
ST100	1840.8	1815.87	1506.75	-	-	-	-	1198.39	1082.89	1345.25	1563.06	-	1512.56	1267.92
ST110			1635.91	-	-	-	-	1276.71	1153.83	1475.39	1742.68	-	1591.32	1286.33
ST111	1770	1825.49	1446.39	-	-	-	-	1020.6	902.6	1239.46	1501.2	-	1251.18	1087.03
Density	8.96	8.93	8.936	8.935	8.935	8.935	8.935	8.759	8.759	8.935	8.935	8.937	-	-

Table S11. A comparison of the properties predicted by the PSO-optimized CG EAM potential with those predicted by the existing all-atom EAM potential for Pt. "-" is added because those properties were not reported by authors.

**Properties**	**Pt**							
	**Target**	**This work**	[27]	[28]	[57]	[32,33]	[57]	[37]
Cohesive energy (eV)	-46.72	-42.23	-46.16	-46.16	-44.048	-46.16	-44.048	-
Poisson Ratio	0.393	0.43	0.473	0.474	0.456	0.38	0.475	-
C12 (GPa)	251	257.27	272.6	273.1	221.9	251.1	227.8	-
C11 (GPa)	347	333.08	304.1	303.1	264.4	410.3	252.1	-
C44 (GPa)	76	75.82	72.086	68.451	69.037	116.195	69.029	-

Bulk Modulus (GPa)	283	282.54	-	-	-	247.7	-	270.9
Surface Tension 100 (mN/m)	2558.4	2567.05	-	-	-	-	-	-
Surface Tension 111 (mN/m)	2460	2469.82	-	-	-	-	-	-
Density (gm/cc)	21.46	23.54	21.509	21.509	20.612	21.509	20.612	-

Section S3: Data Generation and Analysis

S3.1 Sobol Sequences:

The coefficient of variations (CoV) values for all properties with varying levels of IP perturbation are shown in **Figures S1** to **S10**. In the case of Pd, Au, and Ag CG EAM models, the cohesive energy and poisson ratio values were only slightly affected, shown by their lowest CoV for high input parameters perturbation of 4 %. This was followed by density where CoV values were 7.12, 6.99, and 6.98 for Pd, Au, and Ag, respectively. The surface properties for Pd and Ag showed the similar variation as density, followed by highest variation in elastic properties. However, for Au, the CoV for surface properties (7.28 for ST100 and 8.77 for ST111) were higher than CoV for density (6.99), but similar to the elastic properties. In general for all three metals, shear modulus (C44) exhibited the highest CoV of 14.85, 10.46, and 10.66 for Pd, Au, and Ag respectively, indicating that C44 was significantly affected by the 4% input parameters perturbation. On the other hand, for Cu, and Pt, there are substantially higher degrees of variation in properties compared to Pd, Ag, and Au.

Moreover, for the case of Pd, poisson ratio (1.72 for 4% input parameters variation) and cohesive energy (1.89 for 4% input parameters variation) exhibited lowest CoVs, followed by density (7.12 for 4% input parameters variation) and surface tensions at 100 (6.70 for 4% input parameters variation) and 111 (7.53 for 4% input parameters variation) surfaces. The elastic properties of Pd model show a CoV values of 9-15 for a maximum of 4 % input parameters variation, while 4.5-7.5 for 2 % input parameters variation, which suggests excessively high levels of property perturbation with 4 % input parameters variation compared to 2 % and below.

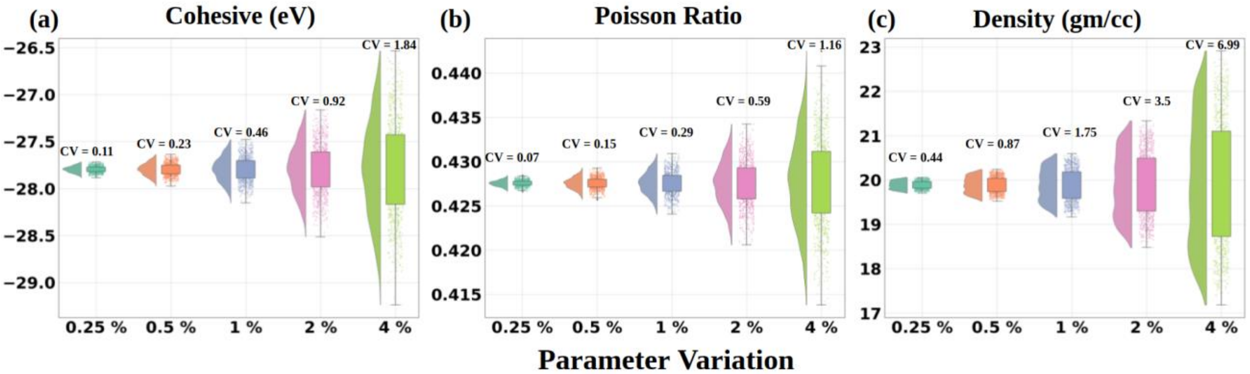

Figure S1. The variation of **(a)** cohesive energy, **(b)** poisson ratio, and **(c)** density calculated *via* CG MD simulations on sobol perturbed input parameters for CG EMA model of Au.

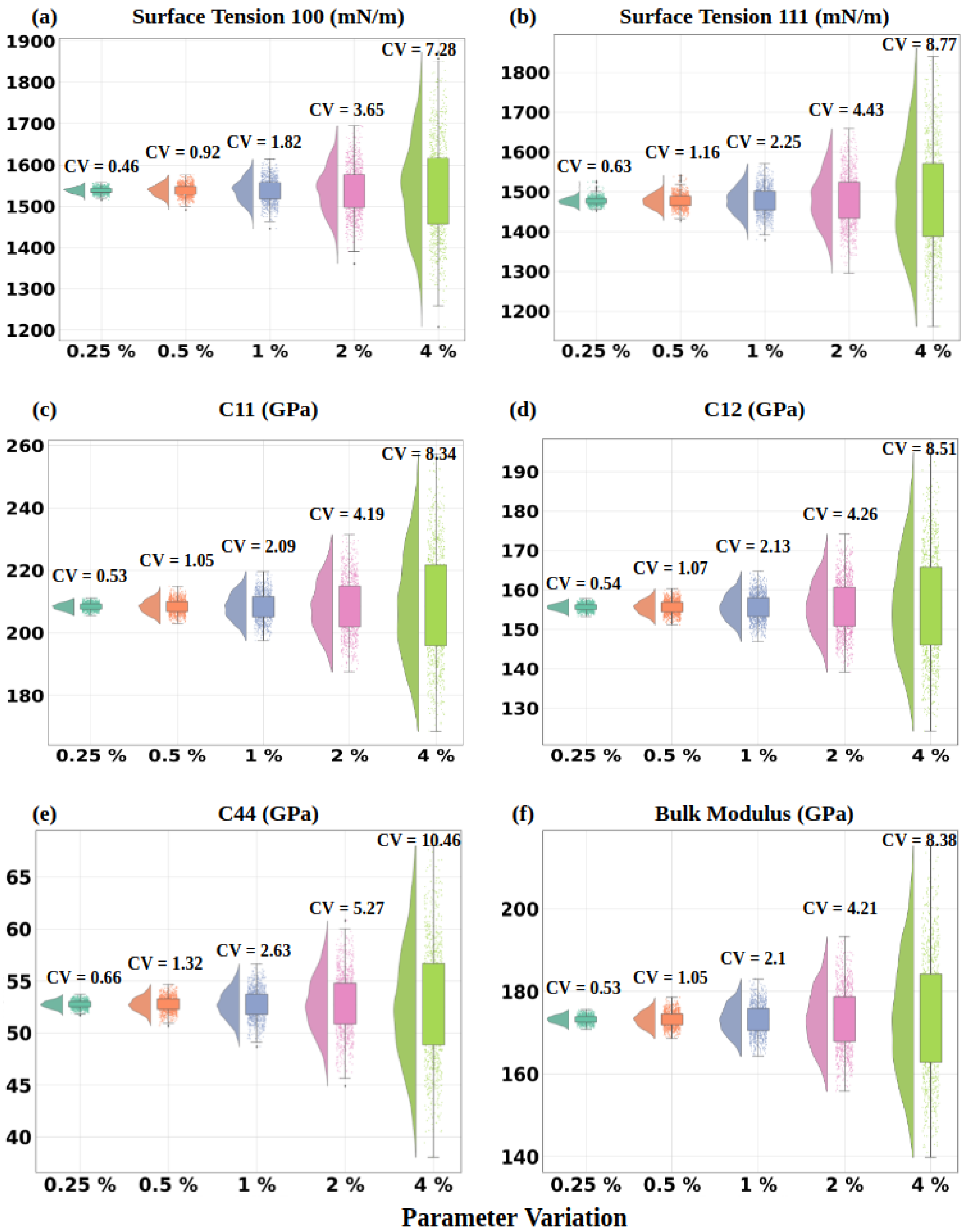

Figure S2. The variation of **(a)** surface tension at 100 surface, **(b)** surface tension at 111 surface, **(c)** C11, **(d)** C12, **(e)** C44, and **(f)** bulk modulus, calculated *via* CG MD simulations on sobol perturbed input parameters for CG EMA model of Au.

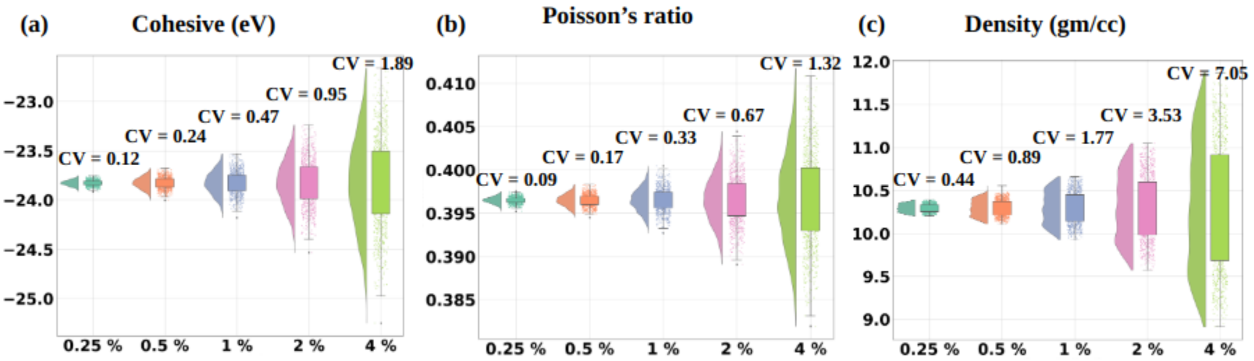

Figure S3. The variation of **(a)** cohesive energy, **(b)** poisson ratio, and **(c)** density calculated *via* CG MD simulations on sobol perturbed input parameters for CG EMA model of Ag.

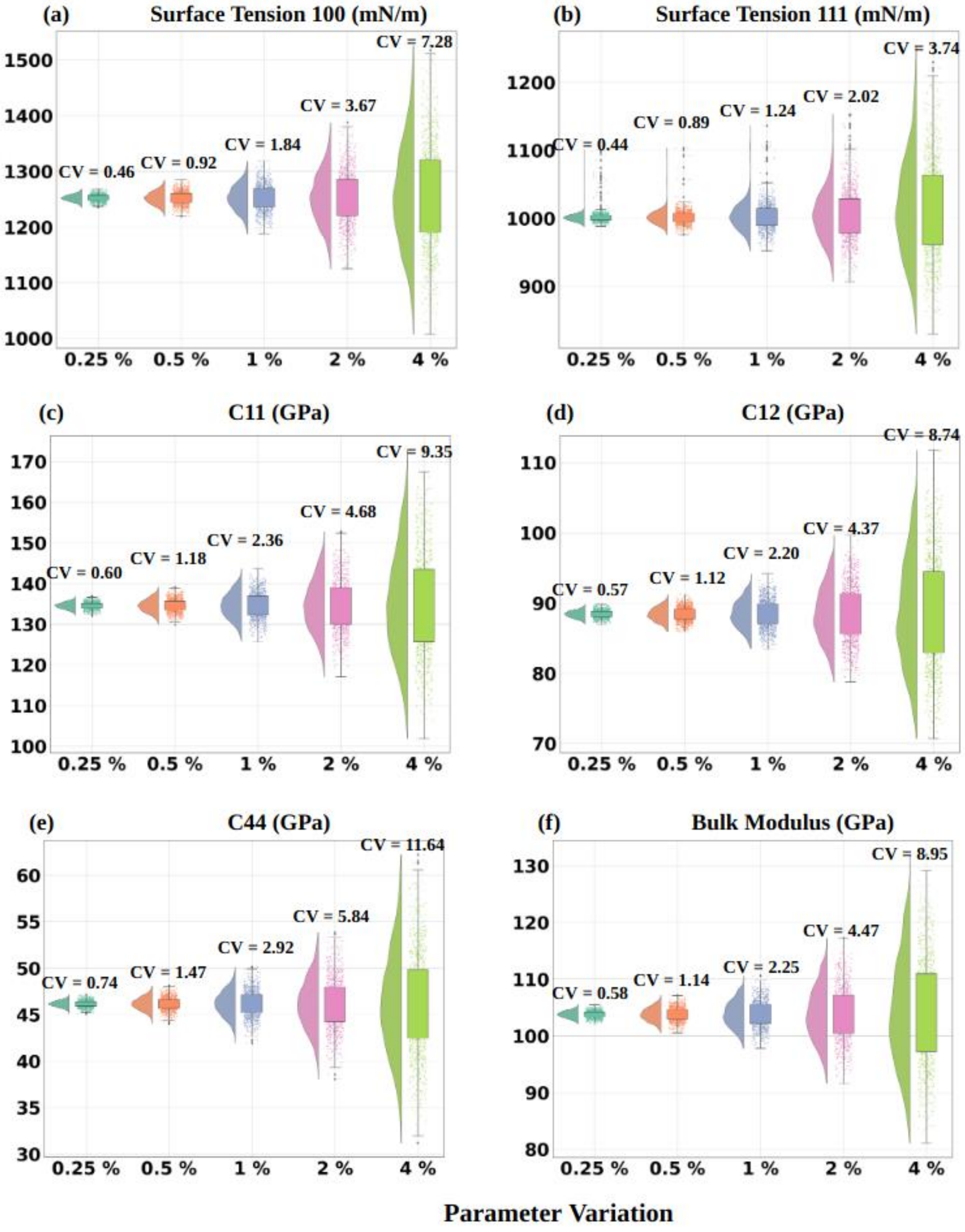

Figure S4. The variation of **(a)** surface tension at 100 surface, **(b)** surface tension at 111 surface, **(c)** C11, **(d)** C12, **(e)** C44, and **(f)** bulk modulus calculated *via* CG MD simulations on sobol perturbed input parameters for CG EMA model of Ag.

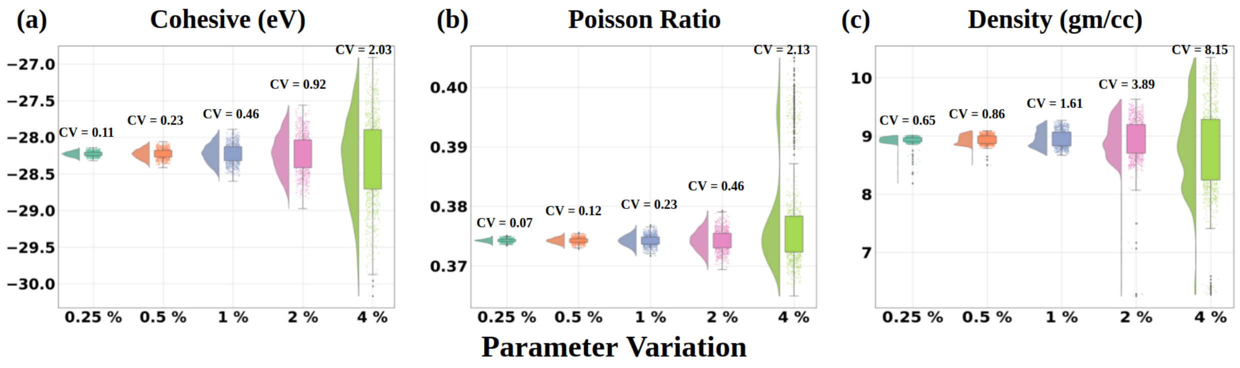

Figure S5. The variation of **(a)** cohesive energy, **(b)** poisson ratio, and **(c)** density calculated *via* CG MD simulations on sobol perturbed input parameters for CG EMA model of Cu.

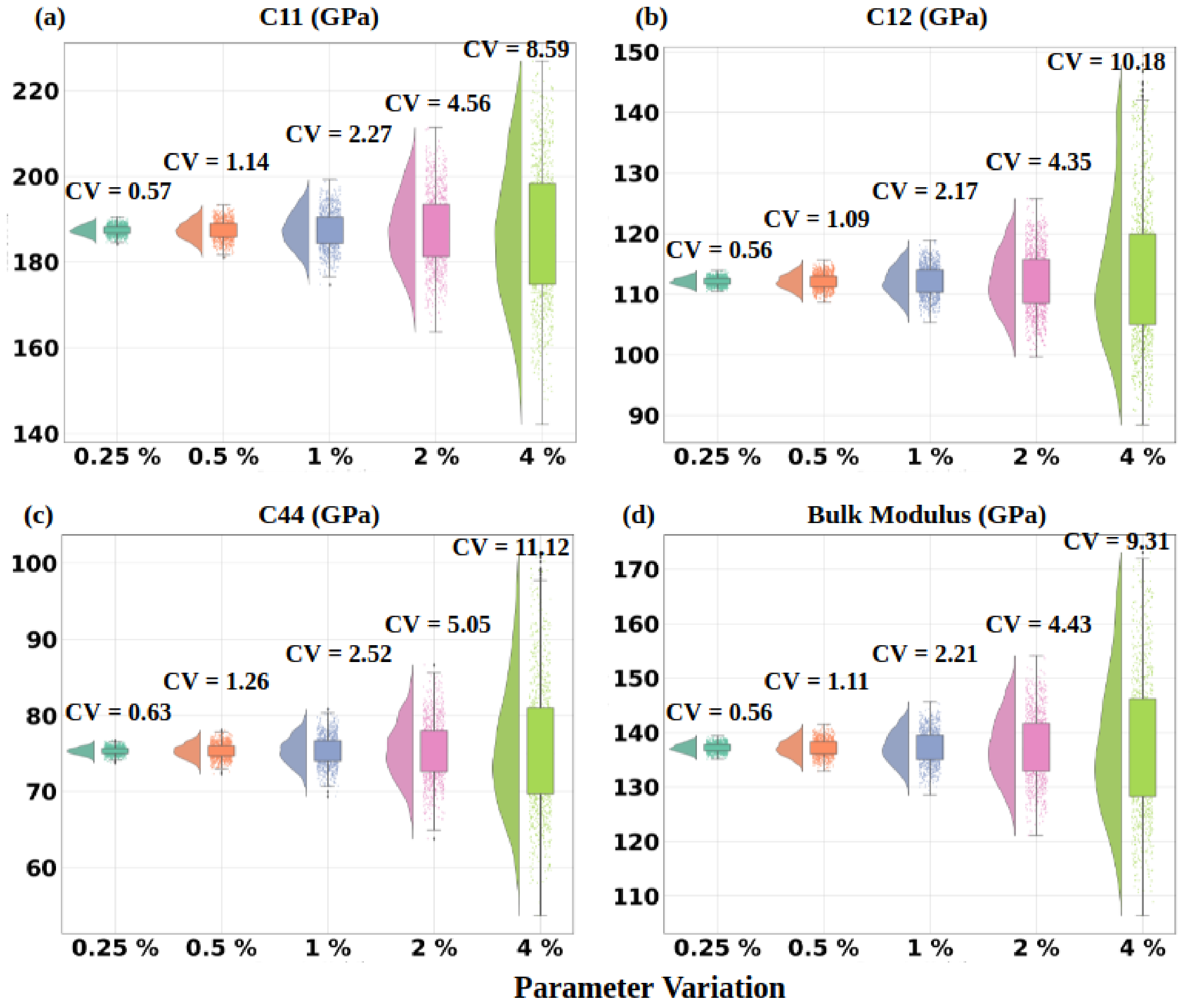

Figure S6. The variation of **(a)** C11, **(b)** C12, **(c)** C44, and **(d)** bulk modulus calculated *via* CG MD simulations on sobol perturbed input parameters for CG EMA model of Cu.

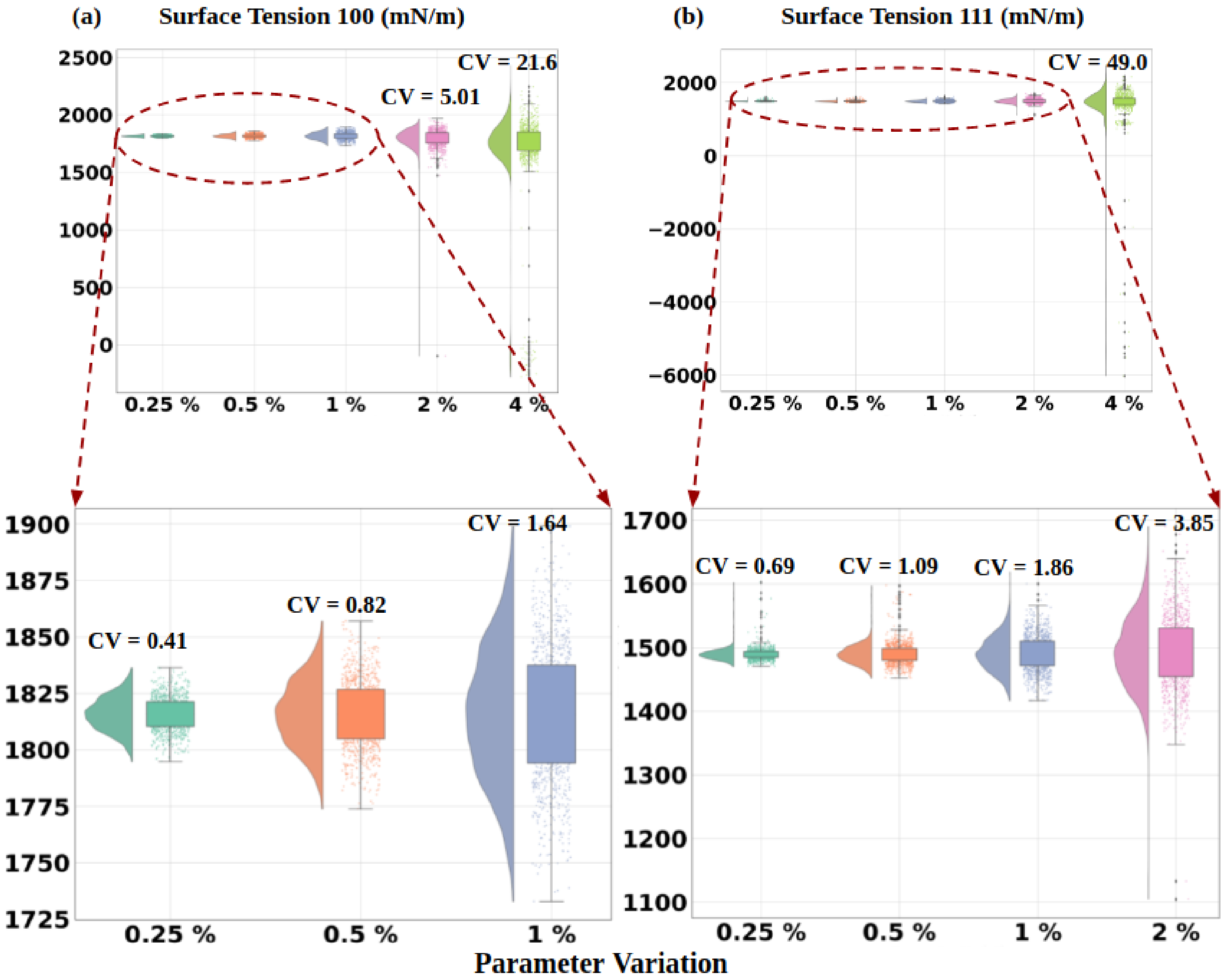

Figure S7. The variation of **(a)** surface tension at 100 surface, and **(b)** surface tension at 111 surface, calculated *via* CG MD simulations on sobol perturbed input parameters for CG EMA model of Cu.

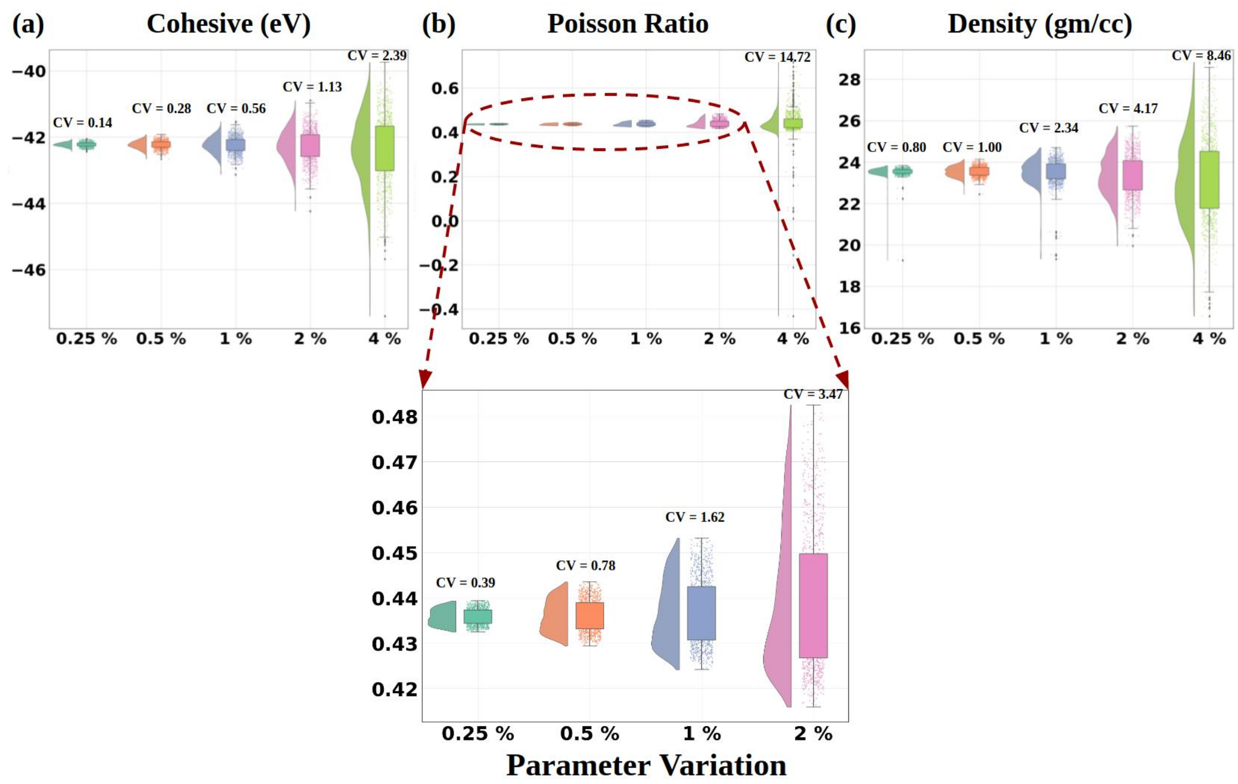

Figure S8. The variation of **(a)** cohesive energy, **(b)** poisson ratio, and **(c)** density calculated *via* CG MD simulations on sobol perturbed input parameters for CG EMA model of Pt.

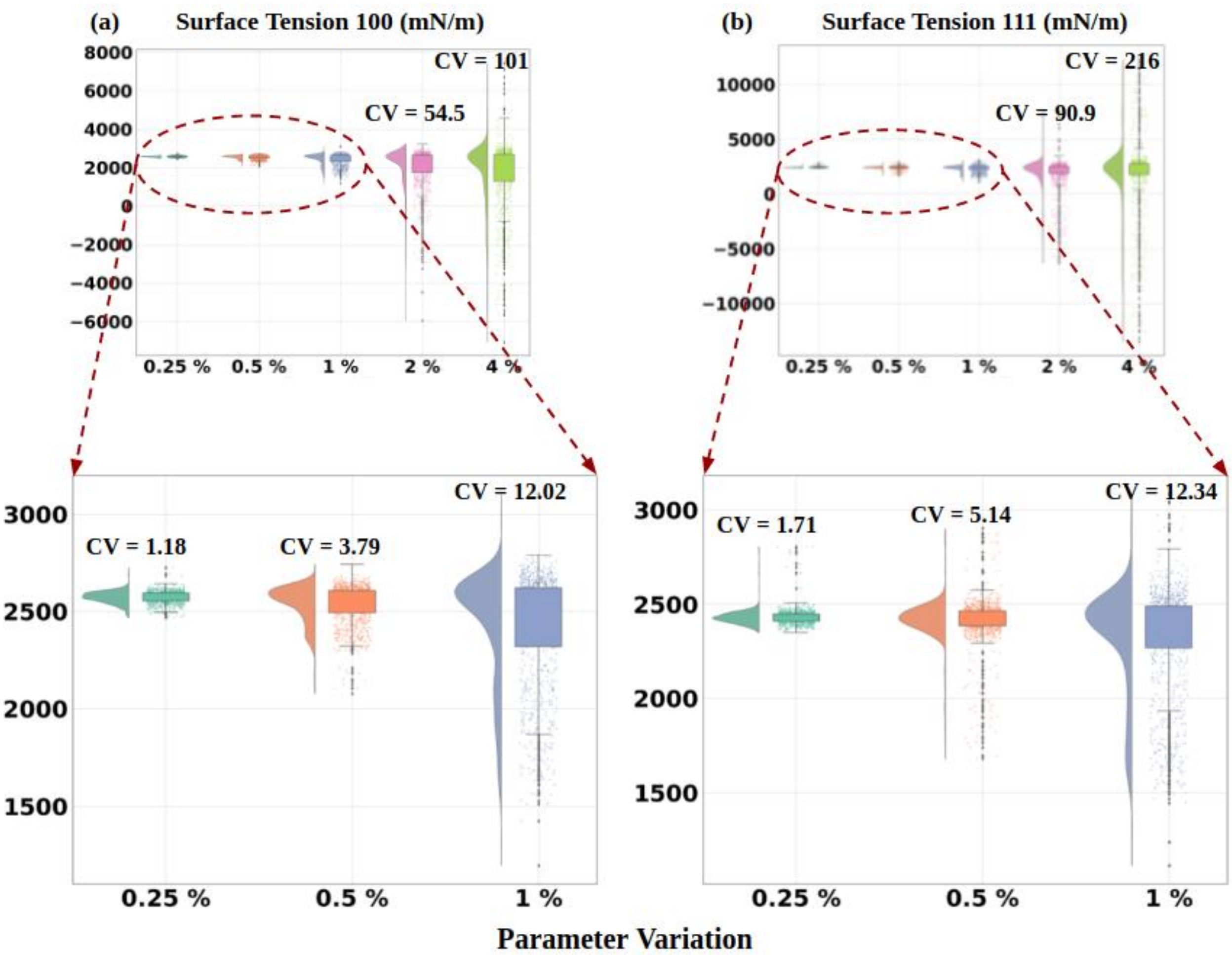

Figure S9. The variation of **(a)** surface tension at 100 surface, and **(b)** surface tension at 111 surface, calculated *via* CG MD simulations on sobol perturbed input parameters for CG EMA model of Pt.

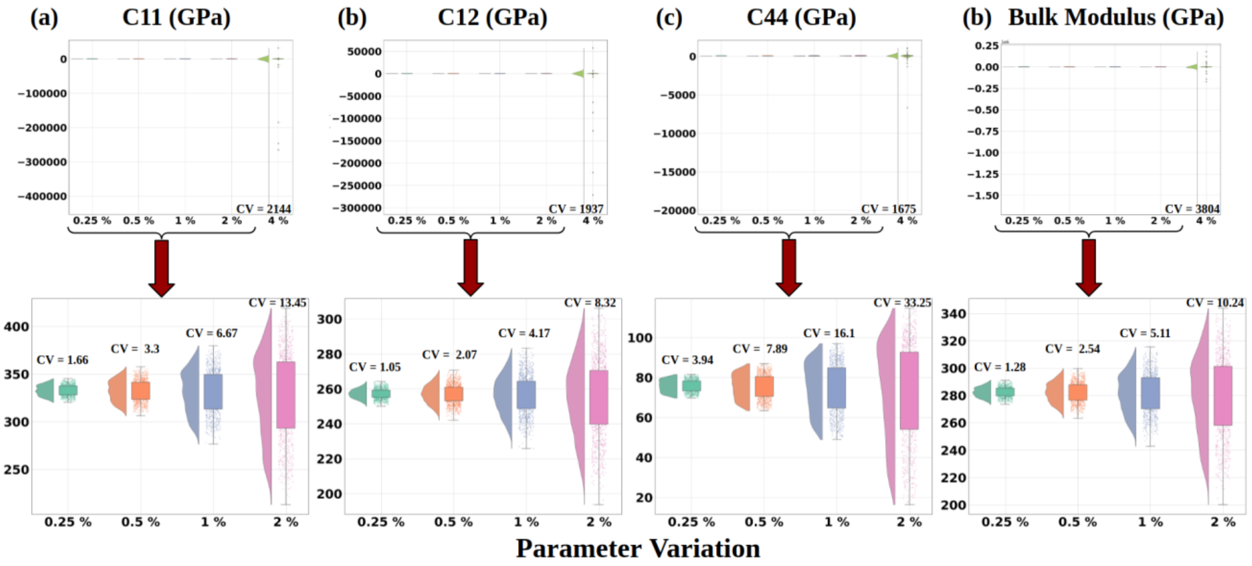

Figure S10. The variation of **(a)** C11, **(b)** C12, **(c)** C44, and **(d)** Bulk modulus calculated *via* CG MD simulations on sobol perturbed input parameters for CG EMA model of Pt.

S3.2 Gaussian Process Regression (GPR) Surrogate Model Training and Development:

Table S12: R^2 values comparing GPR model and MD predictions for each metal are shown, along with the optimal kernel used for each property.

Cohesive Energy	Poisson Ratio	C11	C12	C44	Bulk Modulus	Surface Tension 100	Surface Tension 111	Density
**Pd**								
RBF + RBF	Matern 52	Matern 52	Matern 52	RBF	RBF +RBF	RBF +RBF	Matern 52	RBF +RBF
0.949	0.995	0.996	0.993	0.998	0.994	0.993	0.683	0.983
**Au**								
RBF + RBF	Matern 52	Matern 52	Matern 52	RBF	Matern 52	RBF	RBF + RBF	Matern 52
0.992	0.993	0.997	0.992	0.999	0.992	0.996	0.295	0.999
**Ag**								
RBF	Maten 32	Matern 32	Matern 32	Matern 52	Matern 32	Matern 52	RBF + RBF	RBF
0.999	0.948	0.993	0.984	0.999	0.988	0.999	0.967	0.999
**Cu**								
RBF	Matern 52	Matern 52	Matern 52	RBF	Matern 52	RBF	Matern 32	RBF + RBF
0.968	0.906	0.995	0.987	0.998	0.992	0.356	0.636	0.817
**Pt**								
RBF	RBF	RBF	Matern 52	RBF + RBF	RBF + RBF	Exponential	RBF	RBF
0.956	0.752	0.885	0.909	0.869	0.897	-0.032	0.255	0.81

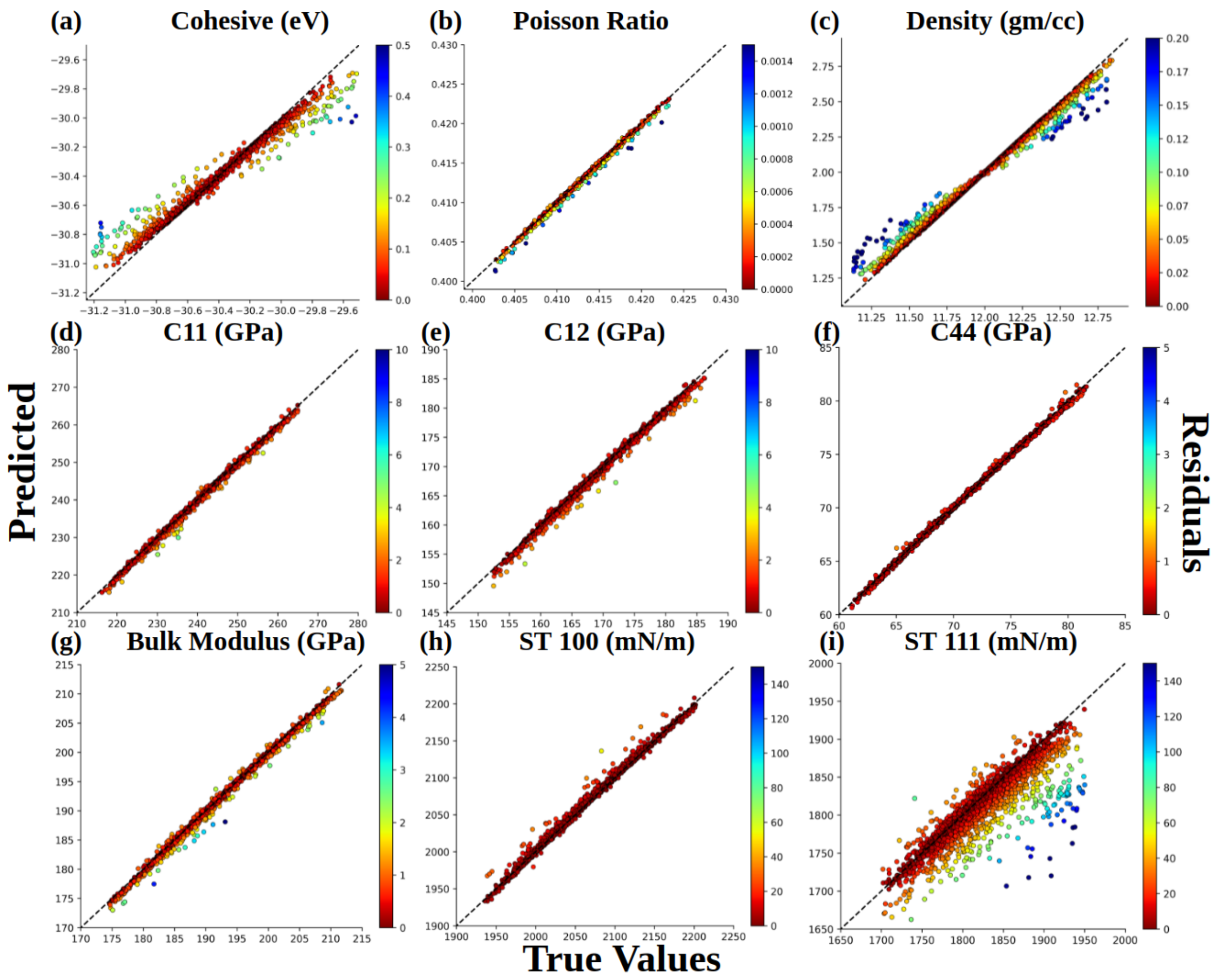

Figure S11. The performance of GP models represented by prediction versus true value of the properties specifically **(a)** Cohesive energy (in eV), **(b)** Poisson's ratio, **(c)** Density (in g/cc), **(d)** C11 (in GPa), **(e)** C12 (in GPa), and **(f)** C44 (in GPa), **(g)** Bulk Modulus (in GPa), **(h)** Surface Tension at 100 surface (in mN/m), and **(i)** Surface Tension at 111 surface (in mN/m) for the Pd model.

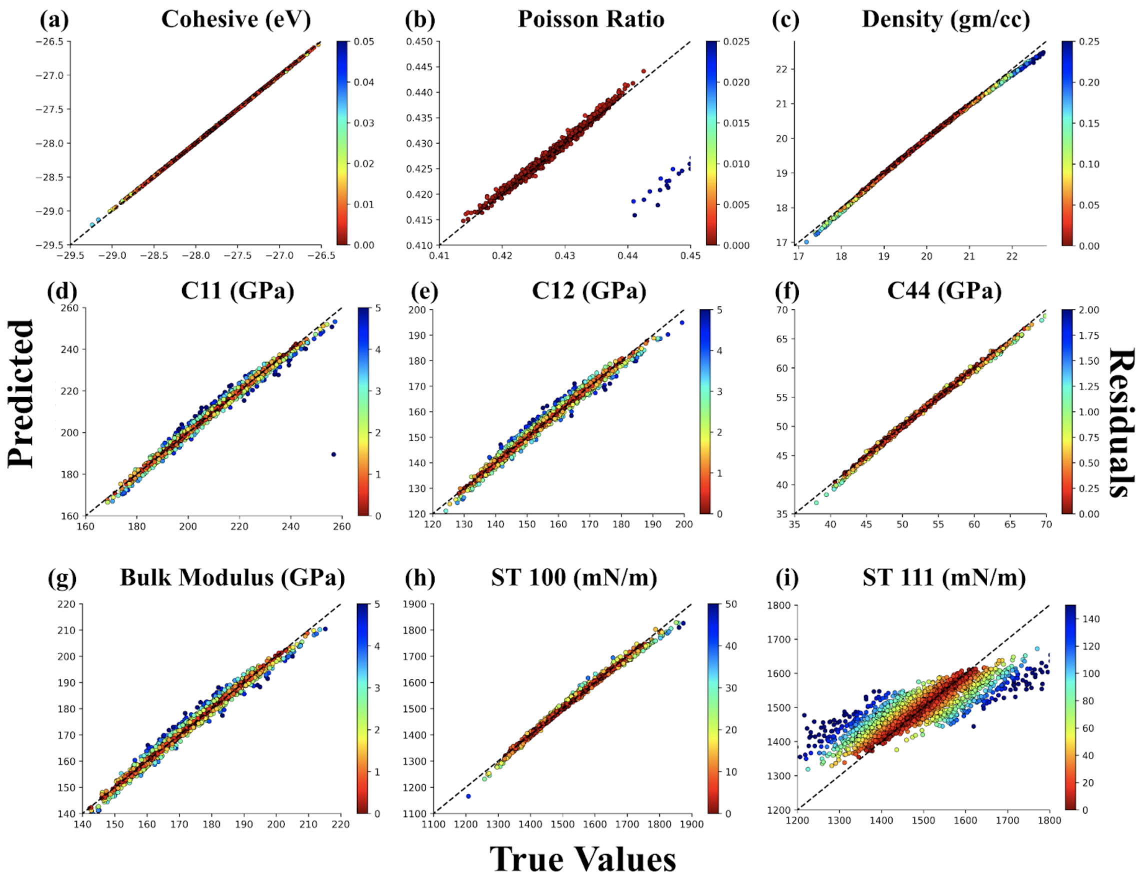

Figure S12. The performance of GP models represented by prediction versus true value of the properties specifically **(a)** Cohesive energy (in eV), **(b)** Poisson's ratio, **(c)** Density (in g/cc), **(d)** C11 (in GPa), **(e)** C12 (in GPa), and **(f)** C44 (in GPa), **(g)** Bulk Modulus (in GPa), **(h)** Surface Tension at 100 surface (in mN/m), and **(i)** Surface Tension at 111 surface (in mN/m) for the Au model.

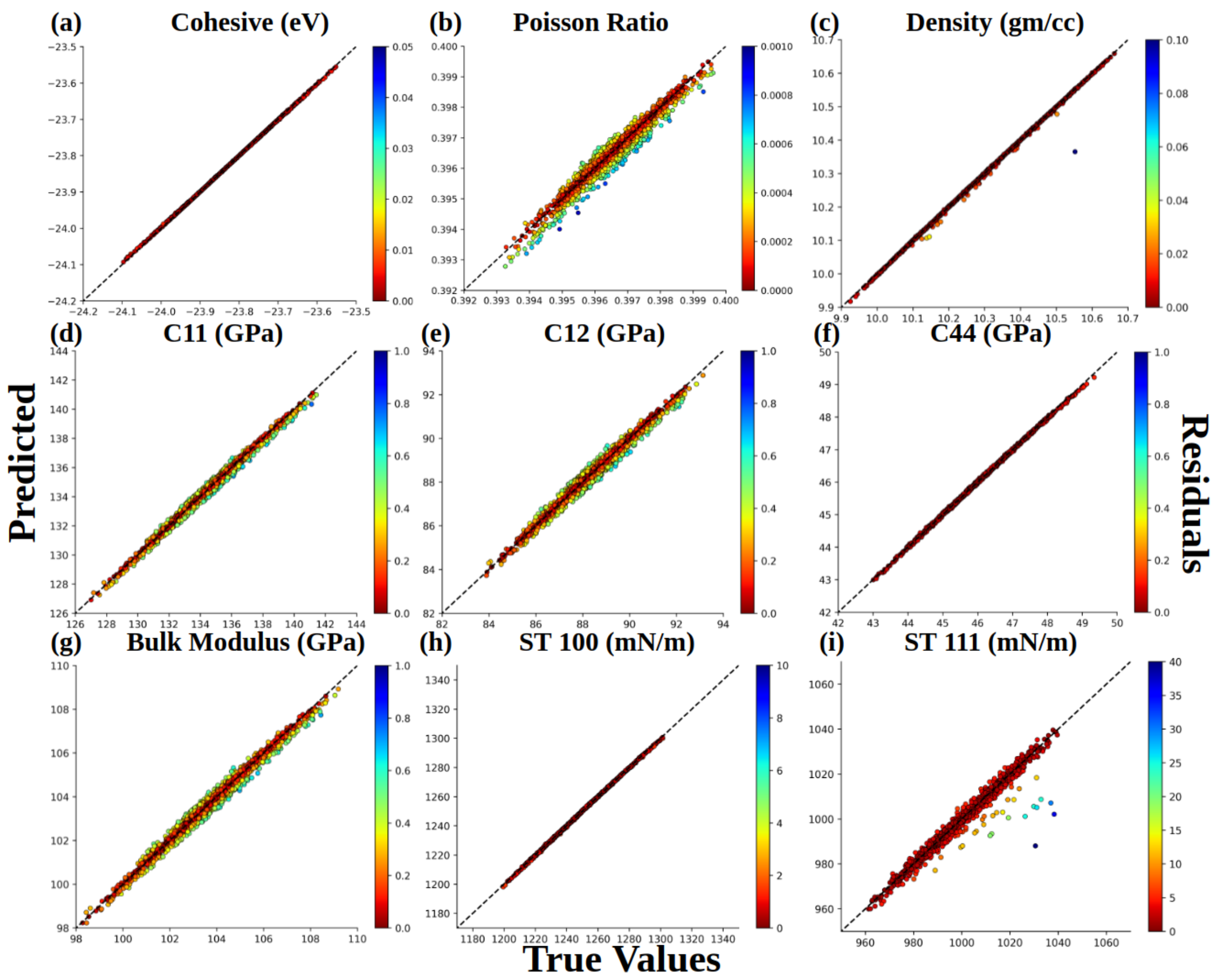

Figure S13. The performance of GP models represented by prediction versus true value of the properties specifically **(a)** Cohesive energy (in eV), **(b)** Poisson's ratio, **(c)** Density (in g/cc), **(d)** C11 (in GPa), **(e)** C12 (in GPa), and **(f)** C44 (in GPa), **(g)** Bulk Modulus (in GPa), **(h)** Surface Tension at 100 surface (in mN/m), and **(i)** Surface Tension at 111 surface (in mN/m) for the Ag model.

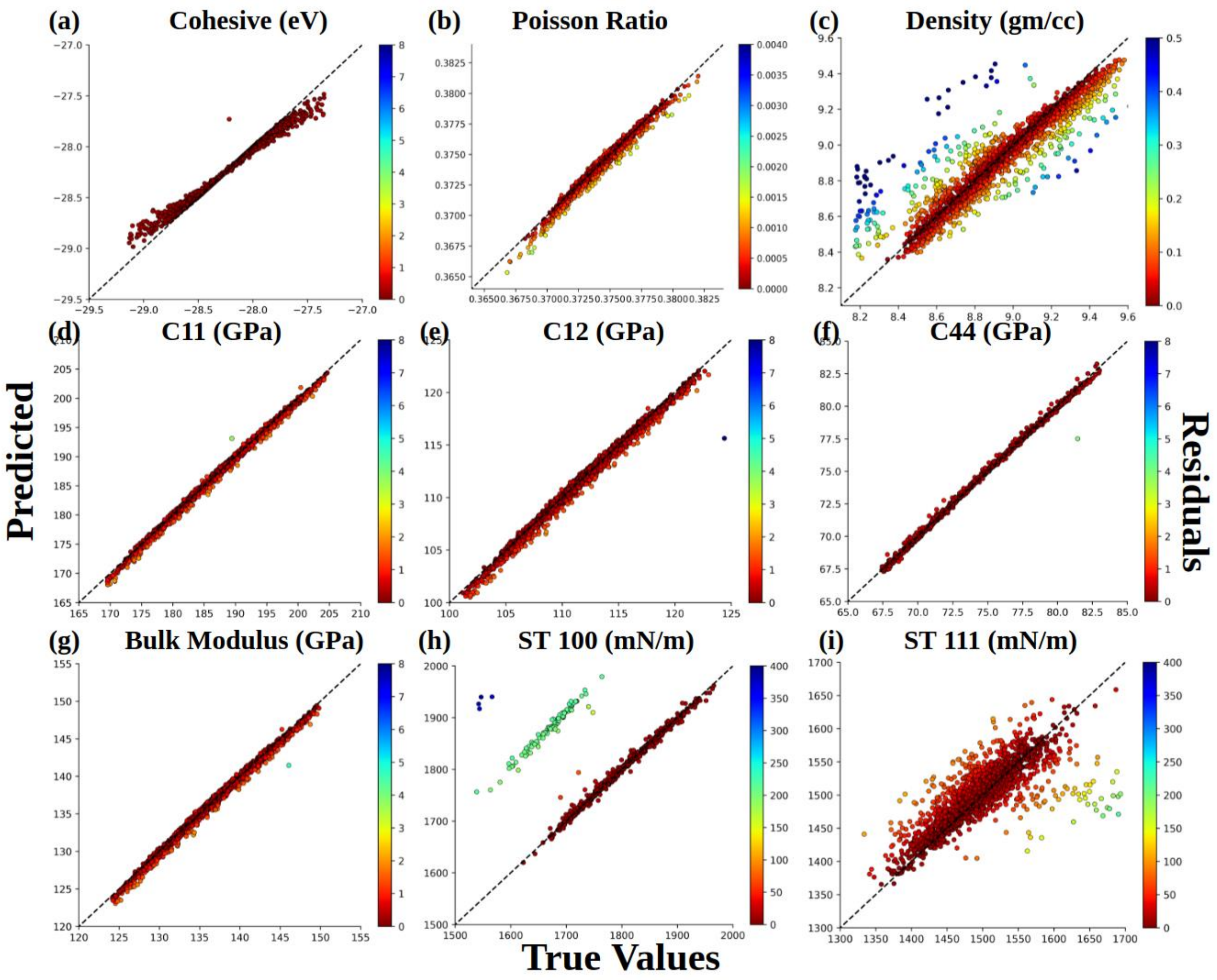

Figure S14. The performance of GP models represented by prediction versus true value of the properties specifically **(a)** Cohesive energy (in eV), **(b)** Poisson's ratio, **(c)** Density (in g/cc), **(d)** C11 (in GPa), **(e)** C12 (in GPa), and **(f)** C44 (in GPa), **(g)** Bulk Modulus (in GPa), **(h)** Surface Tension at 100 surface (in mN/m), and **(i)** Surface Tension at 111 surface (in mN/m) for the Cu model.

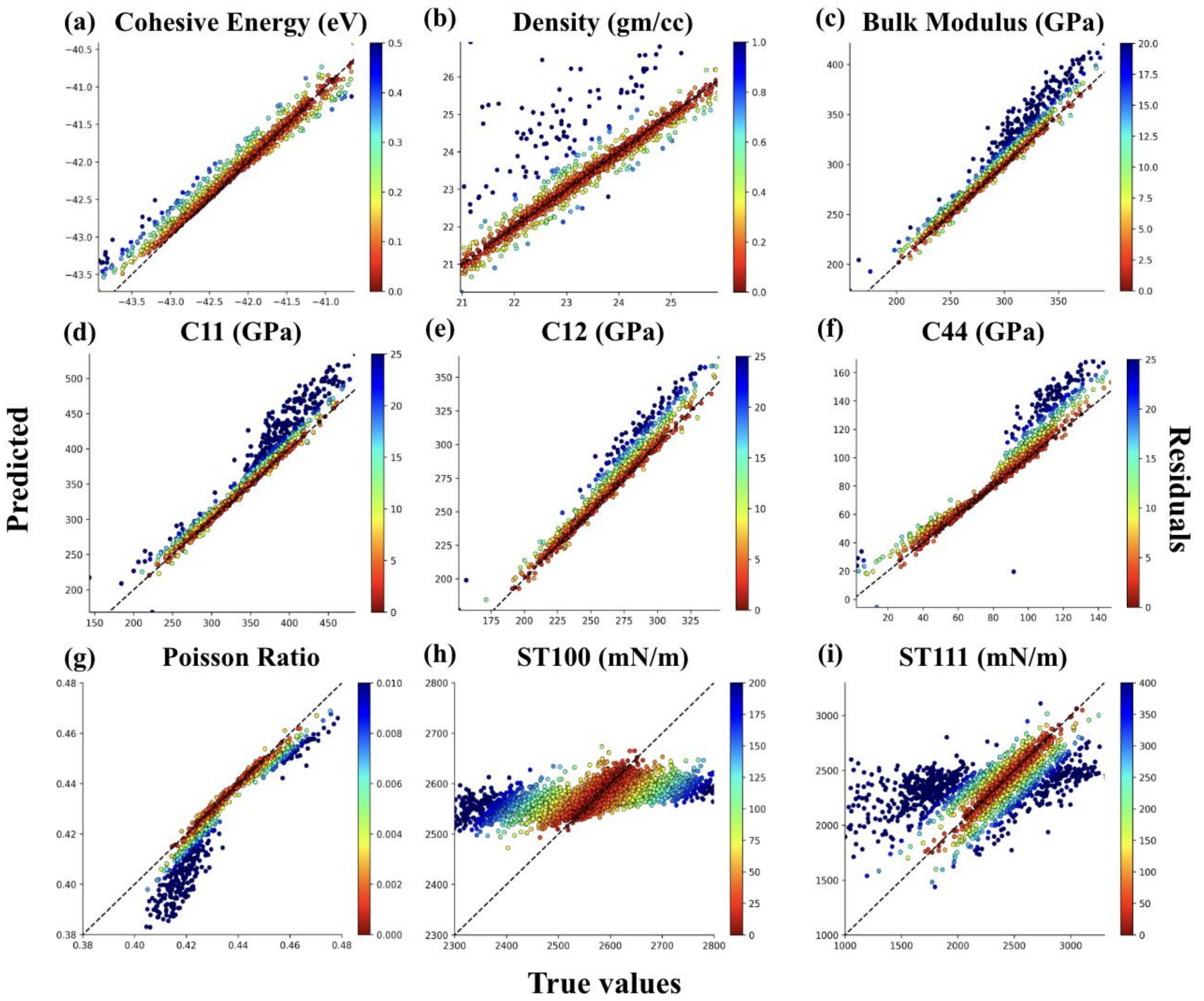

Figure S15. The performance of GP models represented by prediction versus true value of the properties specifically **(a)** Cohesive energy (in eV), **(b)** Poisson's ratio, **(c)** Density (in g/cc), **(d)** C11 (in GPa), **(e)** C12 (in GPa), and **(f)** C44 (in GPa), **(g)** Bulk Modulus (in GPa), **(h)** Surface Tension at 100 surface (in mN/m), and **(i)** Surface Tension at 111 surface (in mN/m) for the Pt model.

Section S4: Bayesian Parameter Estimation and Uncertainty Quantification Analysis

S4.1 Ensemble Slice Sampling Algorithm:

Ensemble Slice Sampling (ESS) is a powerful Markov Chain Monte Carlo (MCMC) method that combines ensemble methods and slice sampling to efficiently sample from complex, high-dimensional distributions. By leveraging the strengths of both approaches, ESS provides an effective way to explore the parameter space and obtain representative samples. Ensemble

methods, first introduced by Goodman and Weare in 2010, utilize multiple walkers or chains to explore the distribution independently. This allows for a more thorough exploration of the parameter space, increasing the chances of finding regions with high posterior density. On the other hand, slice sampling, proposed by Neal in 2003,[58] is a univariate method that involves iteratively sampling points from the posterior distribution by "slicing" through the distribution.[59,60]

1. In ESS, the algorithm starts by initializing an ensemble of points sampled from the prior distribution. One point is randomly selected from the ensemble as the current point.
2. The next step involves choosing a direction vector by taking the difference between the current point and another randomly selected point from the ensemble.
3. A slice level is then drawn uniformly between zero and the probability density of the current point in the chosen direction. This slice level serves as a threshold for accepting or rejecting proposed points. The algorithm defines an interval along the chosen direction vector that contains points with densities above the slice level. This interval is constructed by stepping outwards from the current point until the bracket encompasses the slice.
4. Next, a new point is proposed uniformly within this bracket. If the proposed point lies on the slice (i.e., its probability density is above the slice level), it is accepted as a sample. Otherwise, the bracket is shrunk to exclude the rejected point, and a new point is proposed within the shrunken bracket. This process of proposing points and accepting or rejecting them continues until the ensemble of points adequately represents the posterior distribution.
5. The differential move is a specific type of move used in ESS, where points are drawn along a vector formed by the current point and a random point in the ensemble. This move allows for a more informed exploration of the parameter space by taking advantage of the ensemble information.

In summary, ESS is an advanced MCMC method that combines ensemble methods and slice sampling to efficiently sample from complex, high-dimensional distributions. By utilizing ensemble information and employing the slice sampling technique, ESS provides an effective way

to explore the parameter space and obtain representative samples. The algorithm iteratively selects points, proposes new samples, and adjusts the interval based on acceptance or rejection criteria until an adequate representation of the posterior distribution is achieved.

The primary objective of sampling posterior distributions is to obtain solutions that effectively predict the likelihood. Consequently, the effectiveness of ESS sampling strongly depends on how well the posterior aligns with the likelihood. Therefore, the likelihood remains a critical component. The experimental likelihood, represented by Gaussian distributions characterized by their means and standard deviations, is reported for all metals in **Tables S13** and **S14**.

Table S13: The mean experimental target values and standard deviation to define a likelihood function for Au, Pd, and Cu.

	**Pd**			**Au**			**Ag**		
	**Mean**	**Std. Dev.**	**CoV**	**Mean**	**Std. Dev.**	**CoV**	**Mean**	**Std. Dev.**	**CoV**
Cohesive Energy	-31.28	0.004	0.013	-30.48	0.057	0.186	-23.776	0.053	0.222
Poisson's Ratio	0.374	0.013	3.448	0.42	0.003	0.814	0.337	0.008	2.454
C12	176	12.047	6.845	163	5.408	3.318	93.67	2.619	2.796
C11	234.1	11.815	5.047	192	7.877	4.102	123.99	3.214	2.592
Bulk Modulus	193	11.792	6.11	173	5.53	3.197	104	6.269	6.028
C44	71.2	8.208	11.527	42.3	1.468	3.471	46.12	3.546	7.689
ST100	2059.2	133.92	6.504	1540	60.1	3.903	1237.6	152.003	12.282
ST111	1980	119.619	6.041	1480	60.1	4.061	1190	149.451	12.559
Density	12.023	0.195	1.62	19.3	0.059	0.306	10.49	0.151	1.443

Table S14: The mean experimental target values and standard deviation to define a likelihood function for Ag and Pt.

	**Cu**			**Pt**		
	**Mean**	**Std. Dev.**	**CoV**	**Mean**	**Std. Dev.**	**CoV**

Cohesive Energy	-28.192	0.079	0.281	-46.72	0.095	0.203
Poisson's Ratio	0.323	0.011	3.45	0.393	0.03	7.541
C12	121.4	3.827	3.153	251	12.435	4.954
C11	168.4	6.723	3.992	347	43.05	12.406
Bulk Modulus	137	3.556	2.596	283	27.67	9.778
C44	75.4	10.602	14.061	76	14.112	18.568
ST100	1840.8	266.926	14.501	2558.4	183.338	7.166
ST111	1770	241.697	13.655	2460	248.709	10.11
Density	8.96	0.089	0.992	21.46	0.306	1.424

The Autocorrelation time (ACT) metric was used to analyze convergence of ESS sampling algorithm.[61] It is presented in **Figure S16**. The ACT points intersecting with the unity line represents that convergence was attained.

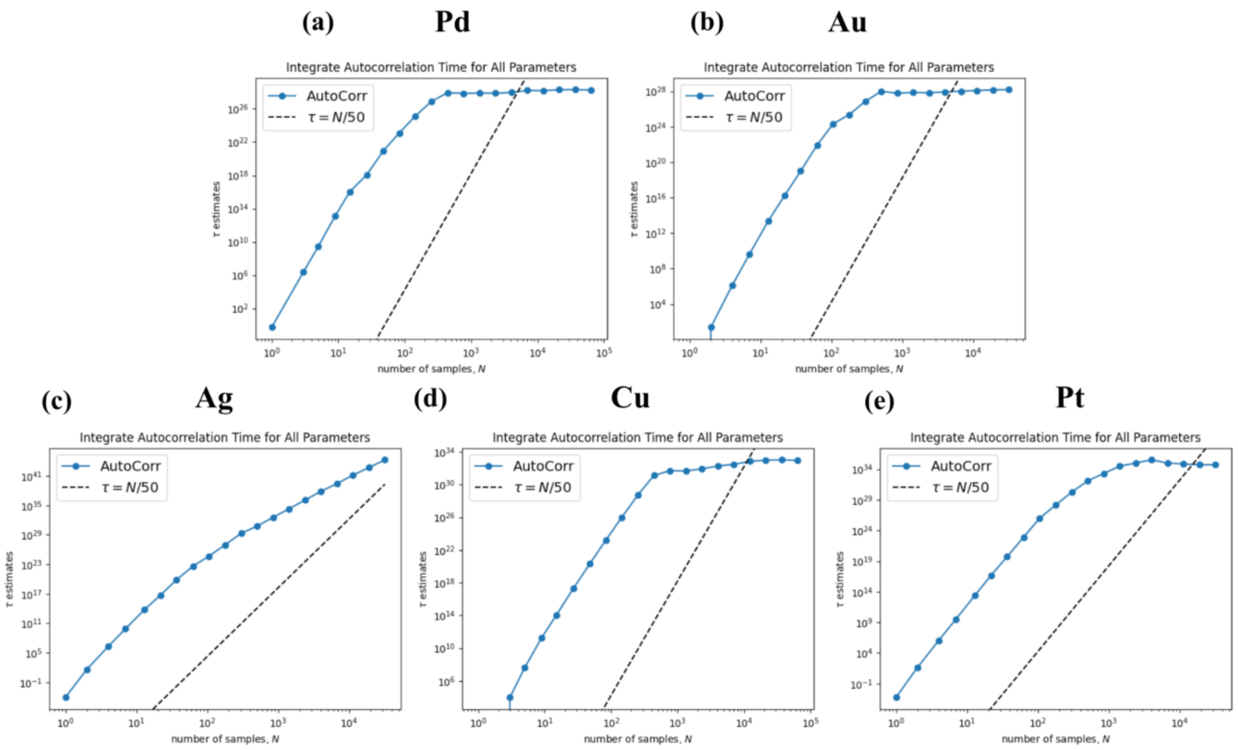

Figure S16. The Autocorrelation time for all parameters of all five metals *viz.* **(a)** Pd, **(b)** Au, **(c)** Ag, **(d)** Cu, and **(e)** Pt as an indication of when the Bayesian parameter estimation using ESS sampling has reached convergence.

S4.2 Bayesian Corner Plots and Pearson’s Correlation Coefficients:

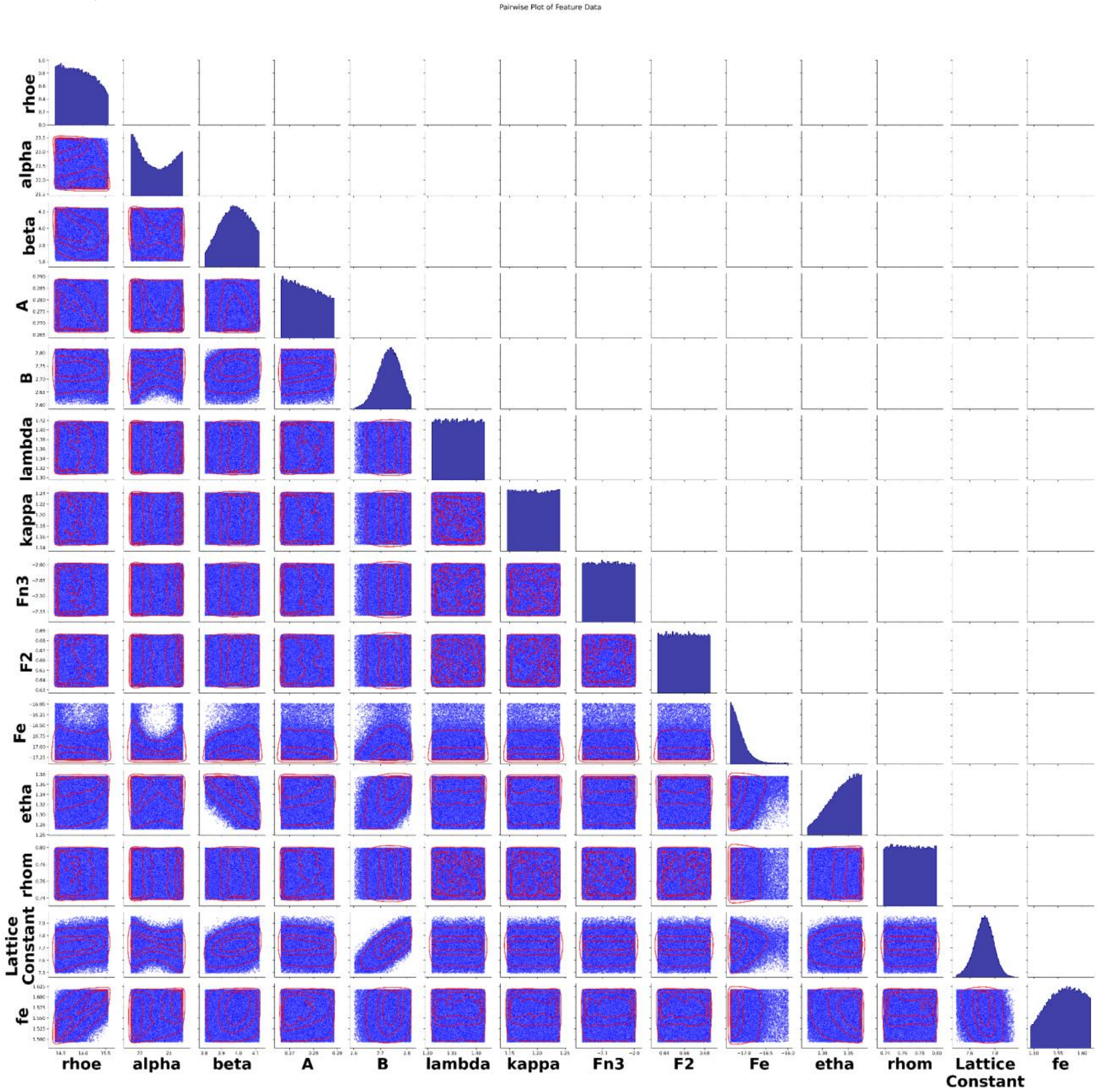

Figure S17. The corner plot of all 14 parameters for Pd estimated by ESS sampler. **Table S15** provides a catalogue of shapes observed in scattered plots including their corresponding symbols and names.

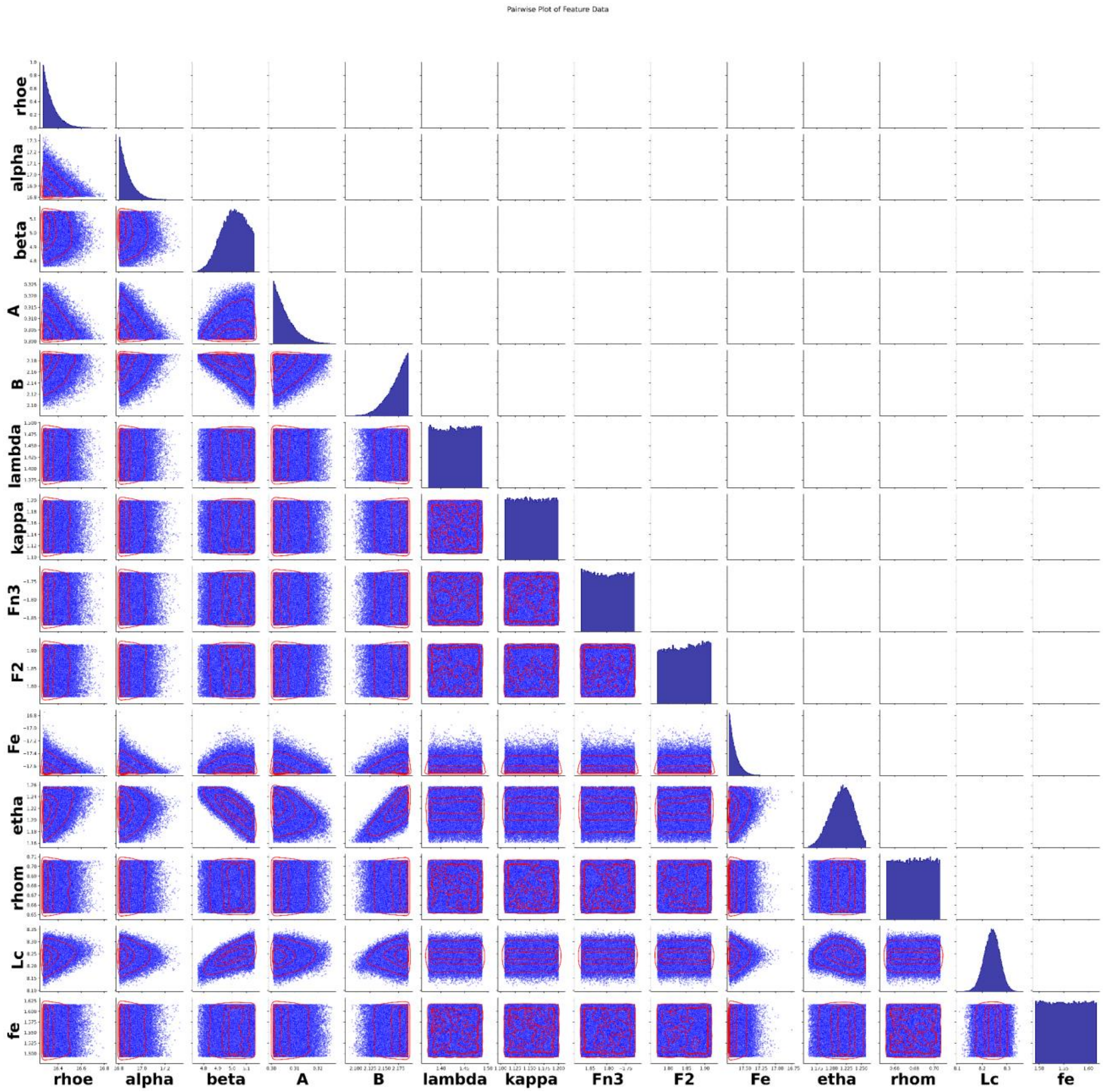

Figure S18. The corner plot of all 14 parameters for Au estimated by ESS sampler. **Table S15** provides a catalogue of shapes observed in scattered plots including their corresponding symbols and names.

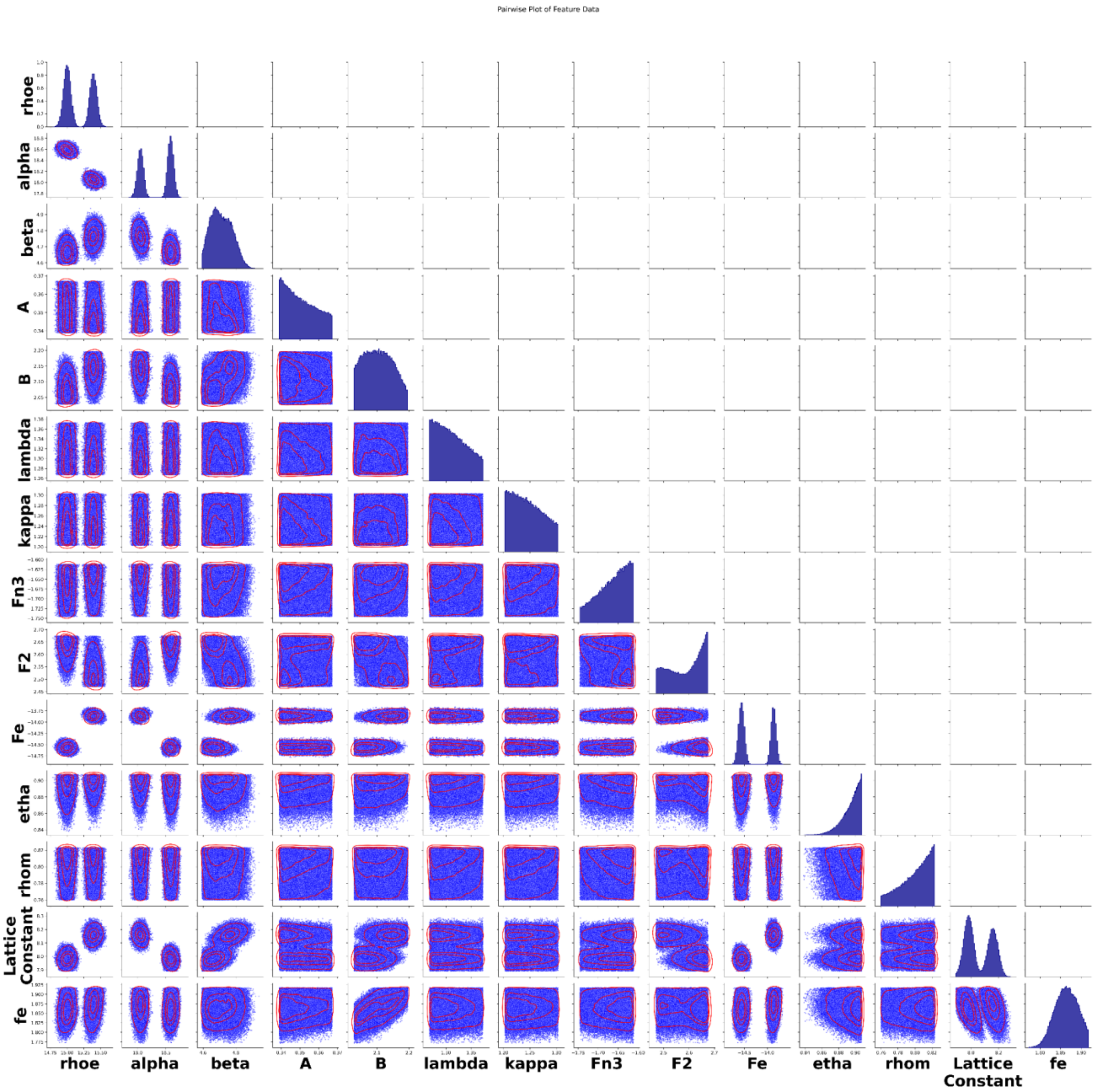

Figure S19. The corner plot of all 14 parameters for Ag estimated by ESS sampler. **Table S15** provides a catalogue of shapes observed in scattered plots including their corresponding symbols and names.

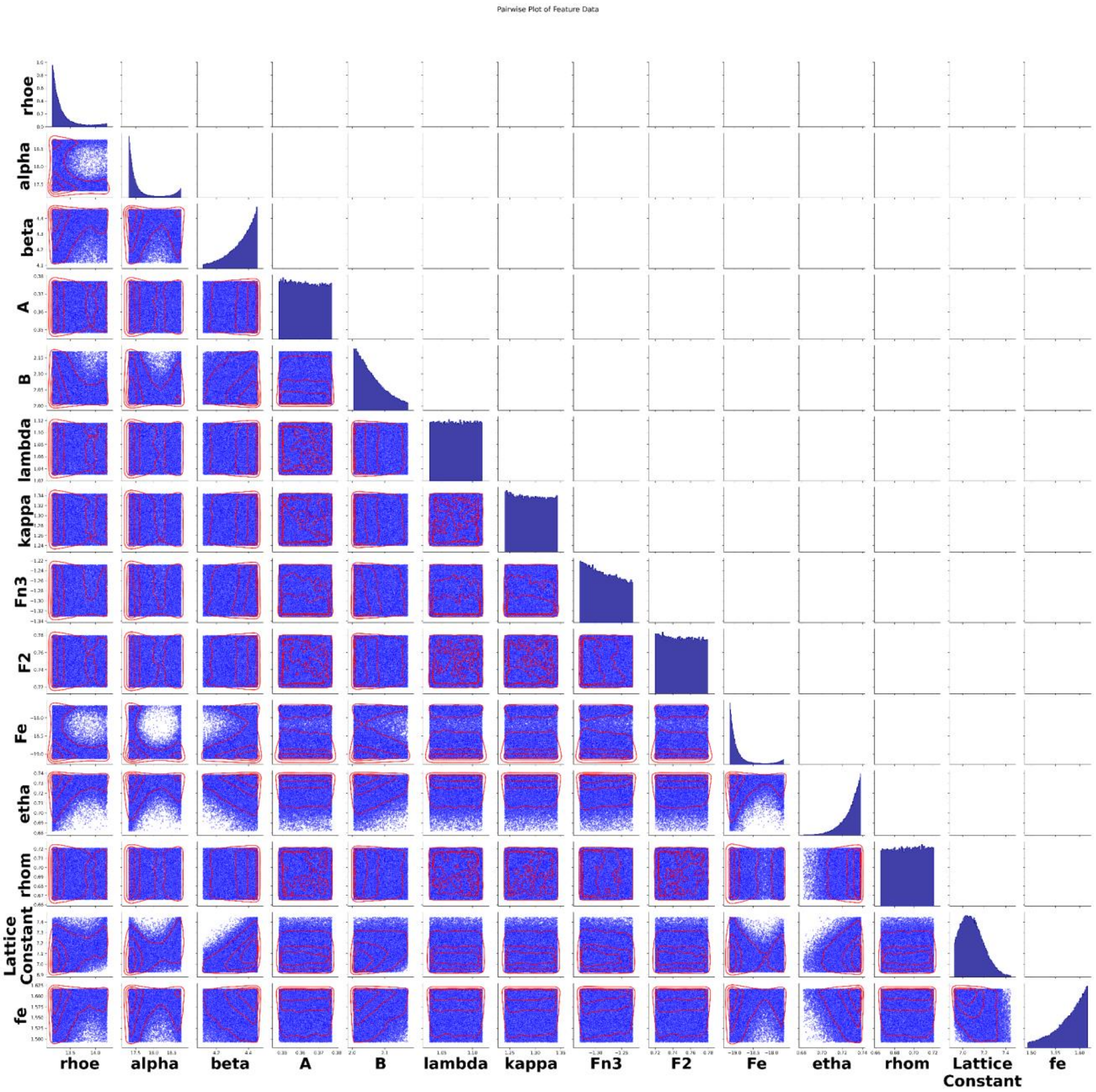

Figure S20. The corner plot of all 14 parameters for Cu estimated by ESS sampler. **Table S15** provides a catalogue of shapes observed in scattered plots including their corresponding symbols and names.

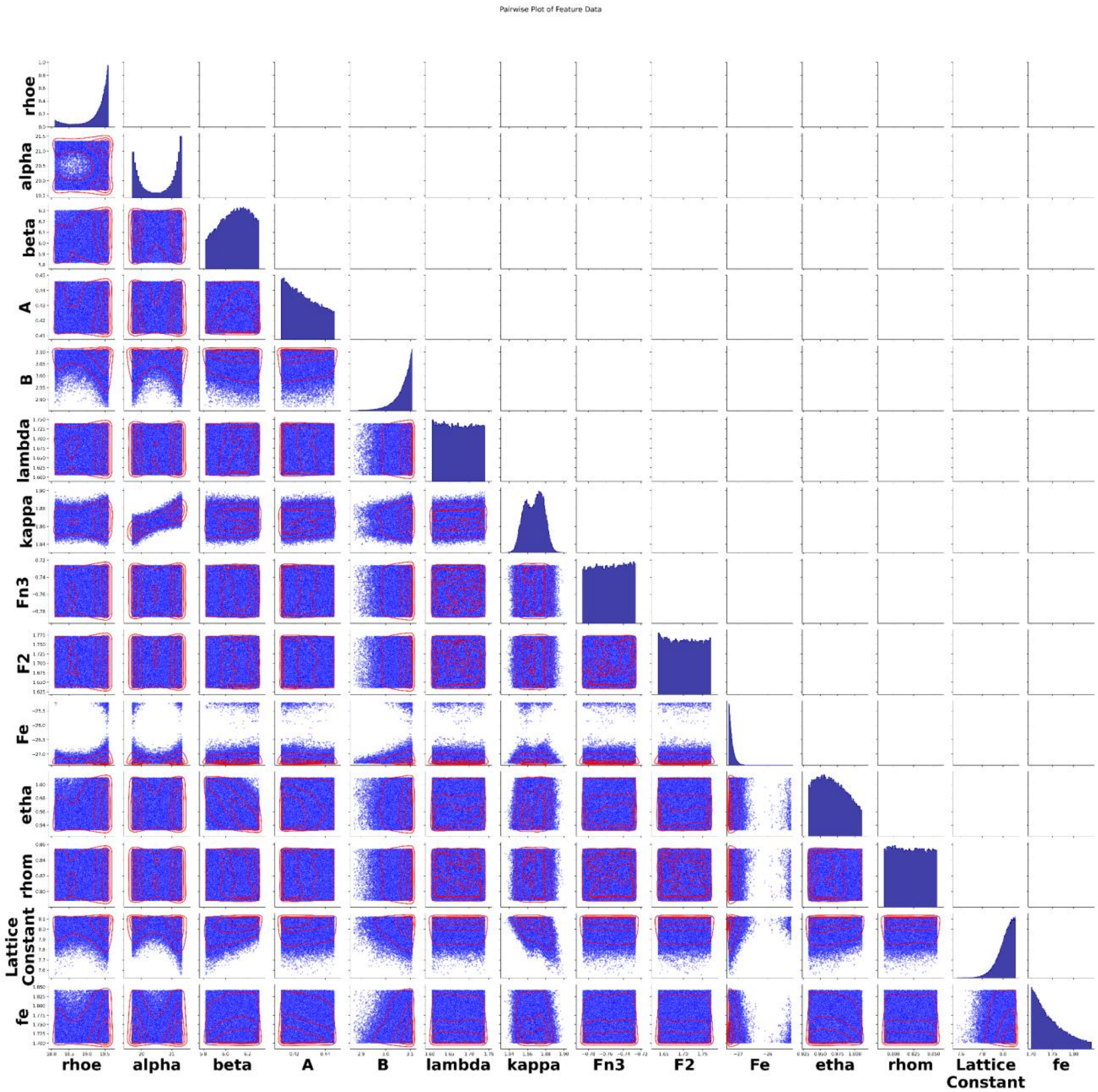

Figure S21. The corner plot of all 14 parameters for Pt estimated by ESS sampler. **Table S15** provides a catalogue of shapes observed in scattered plots including their corresponding symbols and names.

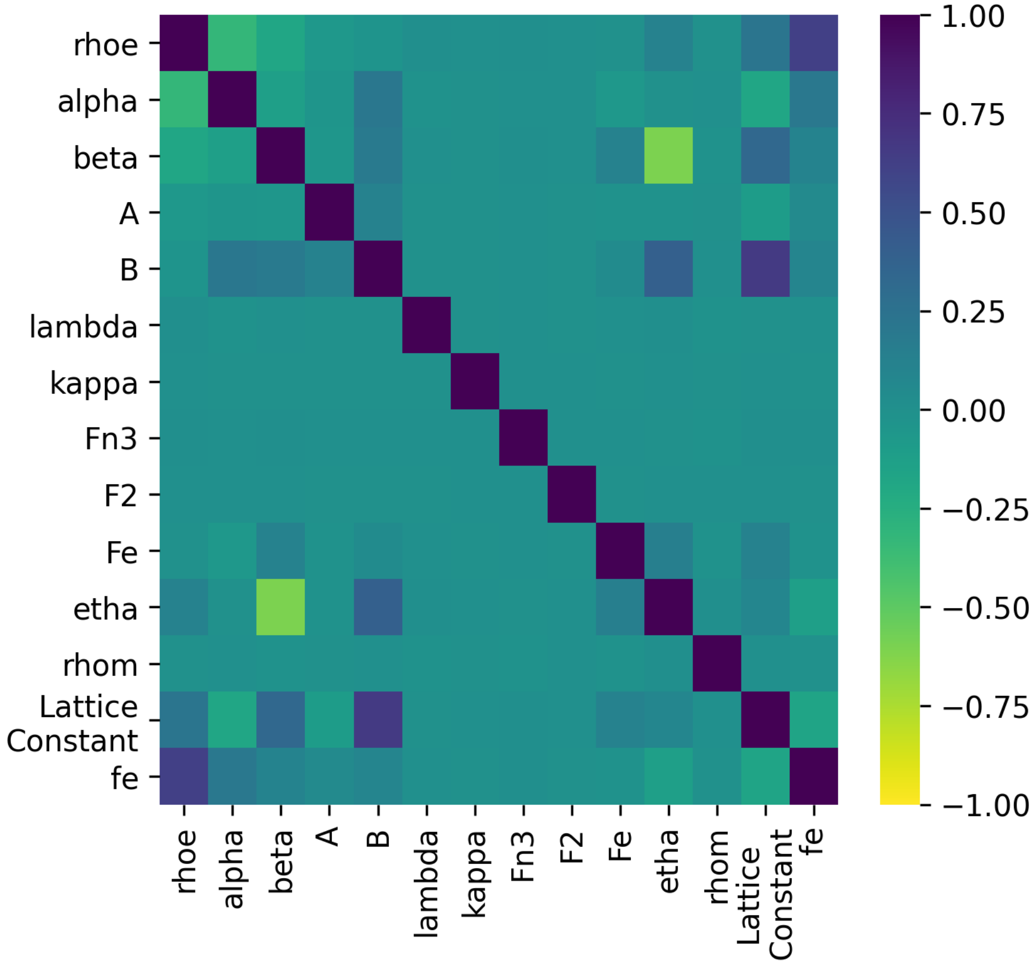

Figure S22. The heatmaps of Pearson correlation coefficients, illustrating the correlation between all 14 CG EAM parameters, for Pd estimated by ESS sampler.

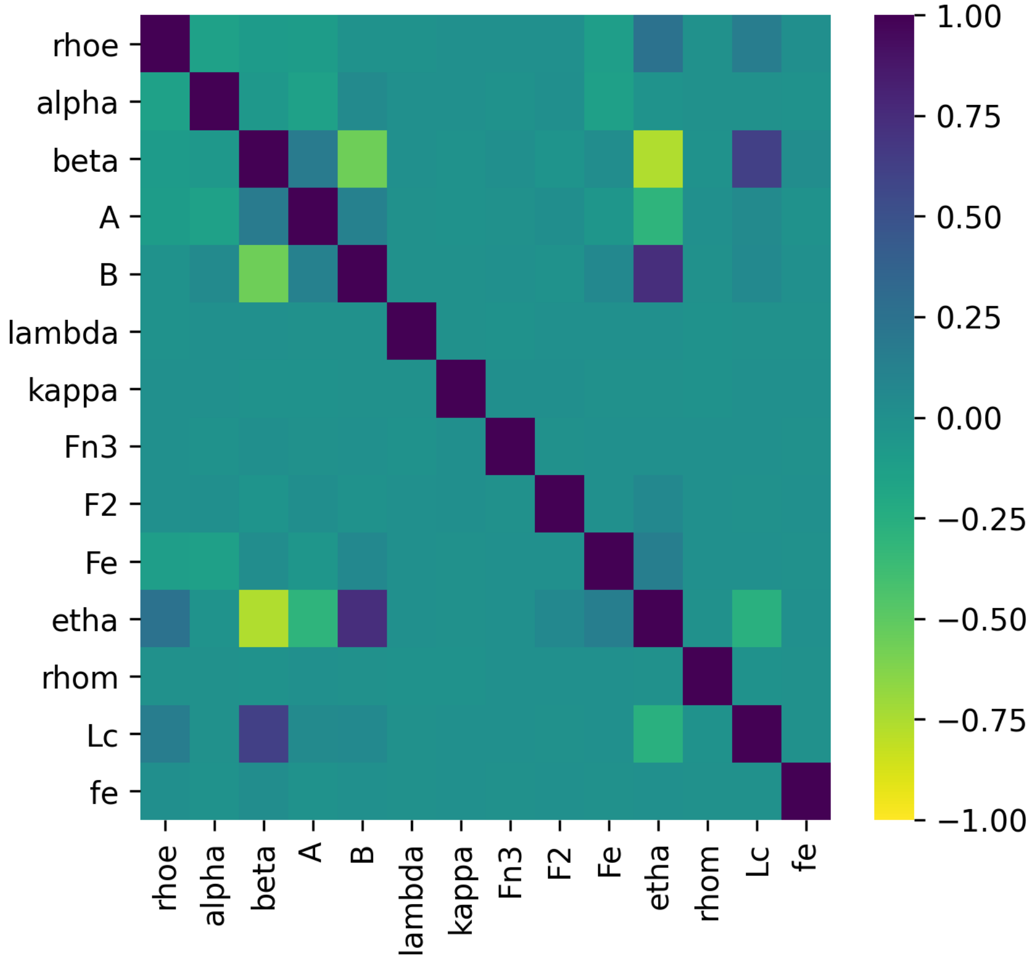

Figure S23. The heatmaps of Pearson correlation coefficients, illustrating the correlation between all 14 CG EAM parameters, for Au estimated by ESS sampler.

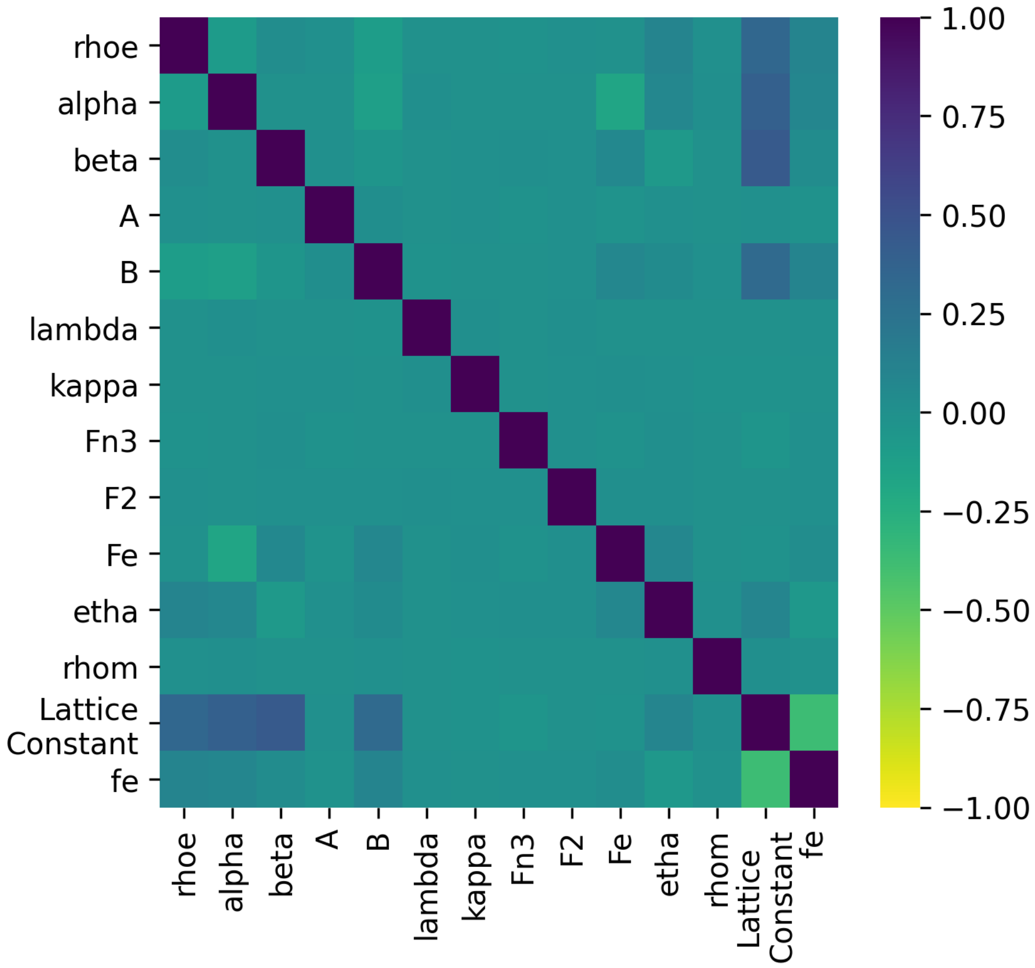

Figure S24. The heatmaps of Pearson correlation coefficients, illustrating the correlation between all 14 CG EAM parameters, for Ag estimated by ESS sampler.

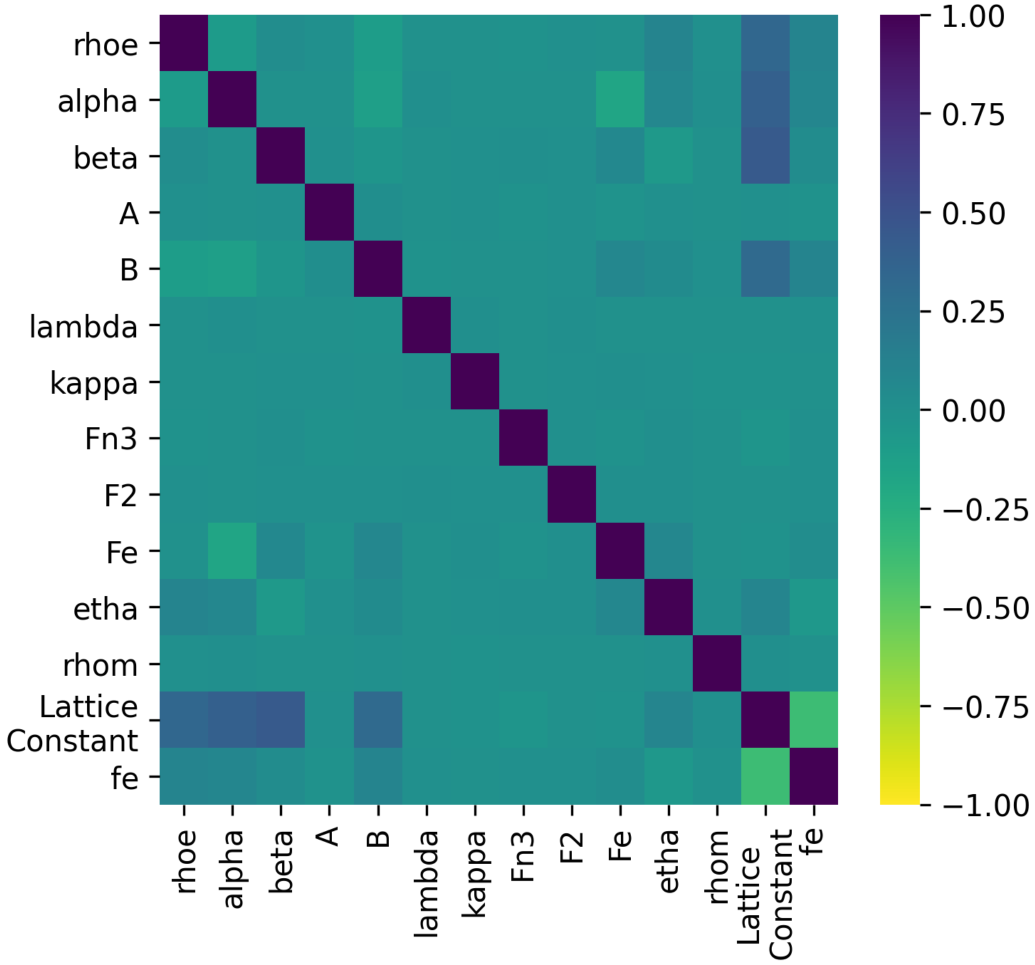

Figure S25. The heatmaps of Pearson correlation coefficients, illustrating the correlation between all 14 CG EAM parameters, for Cu estimated by ESS sampler.

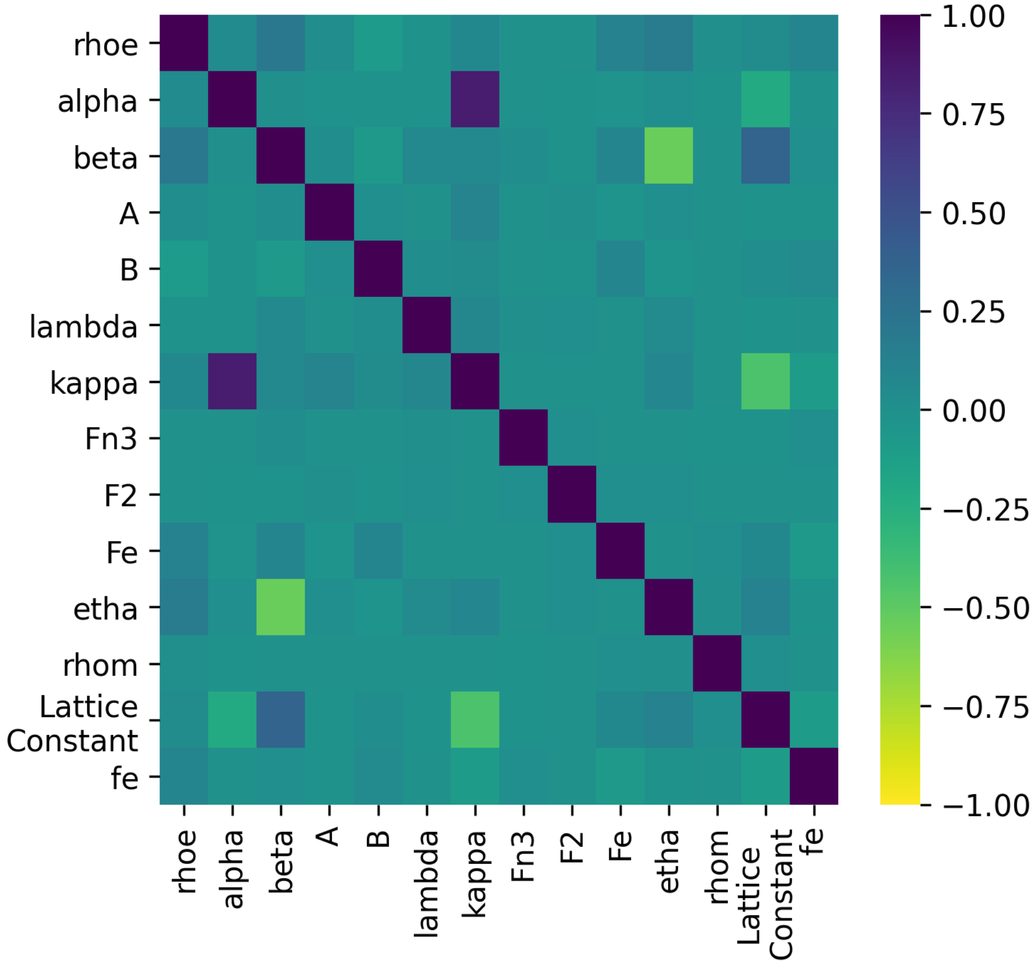

Figure S26. The heatmaps of Pearson correlation coefficients, illustrating the correlation between all 14 CG EAM parameters, for Pt estimated by ESS sampler.

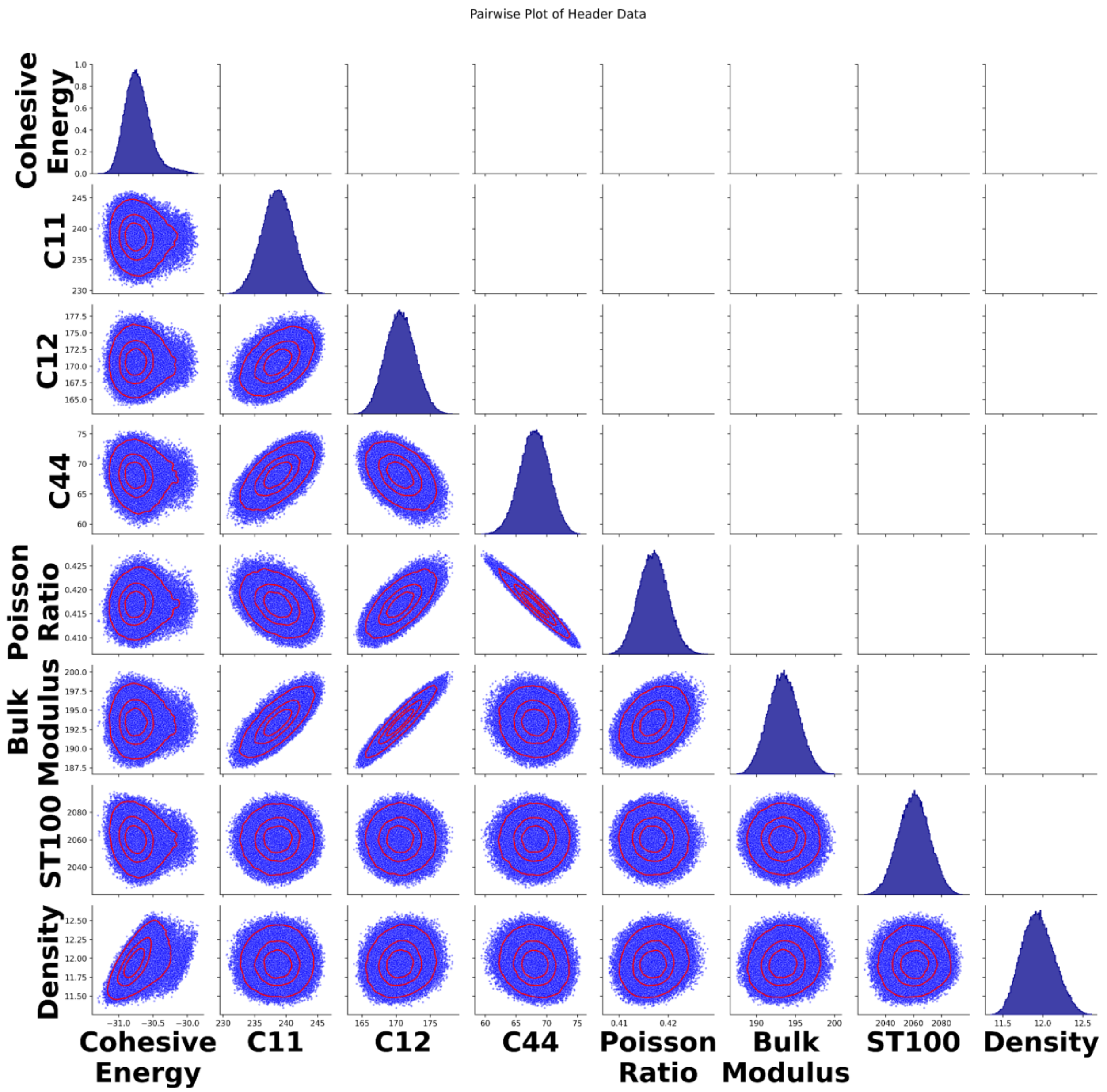

Figure S27. The corner plot of all properties for Pd predicted by surrogate GPR model on ESS estimated parameters. **Table S15** provides a catalogue of shapes observed in scattered plots including their corresponding symbols and names.

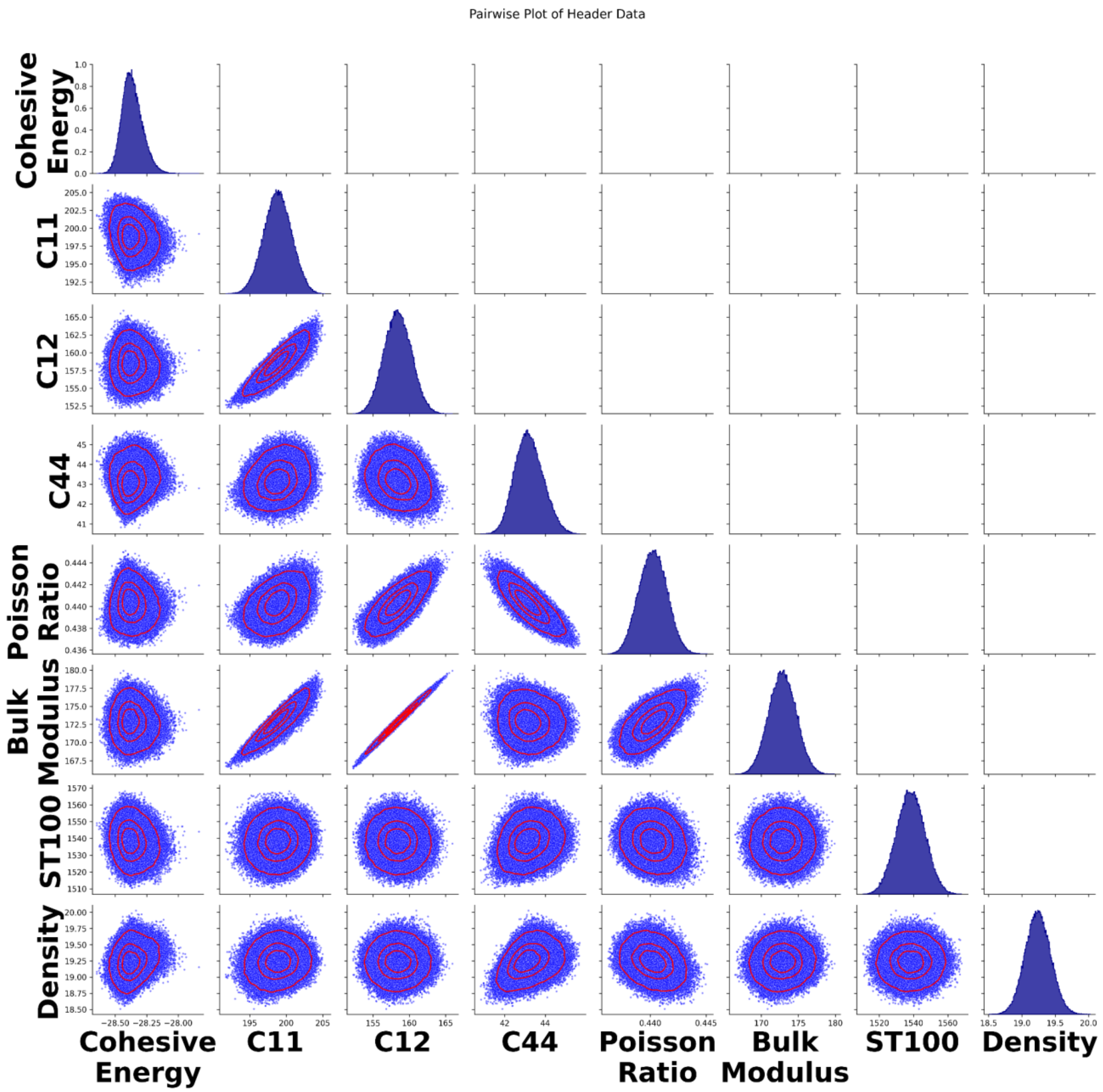

Figure S28. The corner plot of all properties for Au predicted by GP on ESS estimated parameters. **Table S15** provides a catalogue of shapes observed in scattered plots including their corresponding symbols and names.

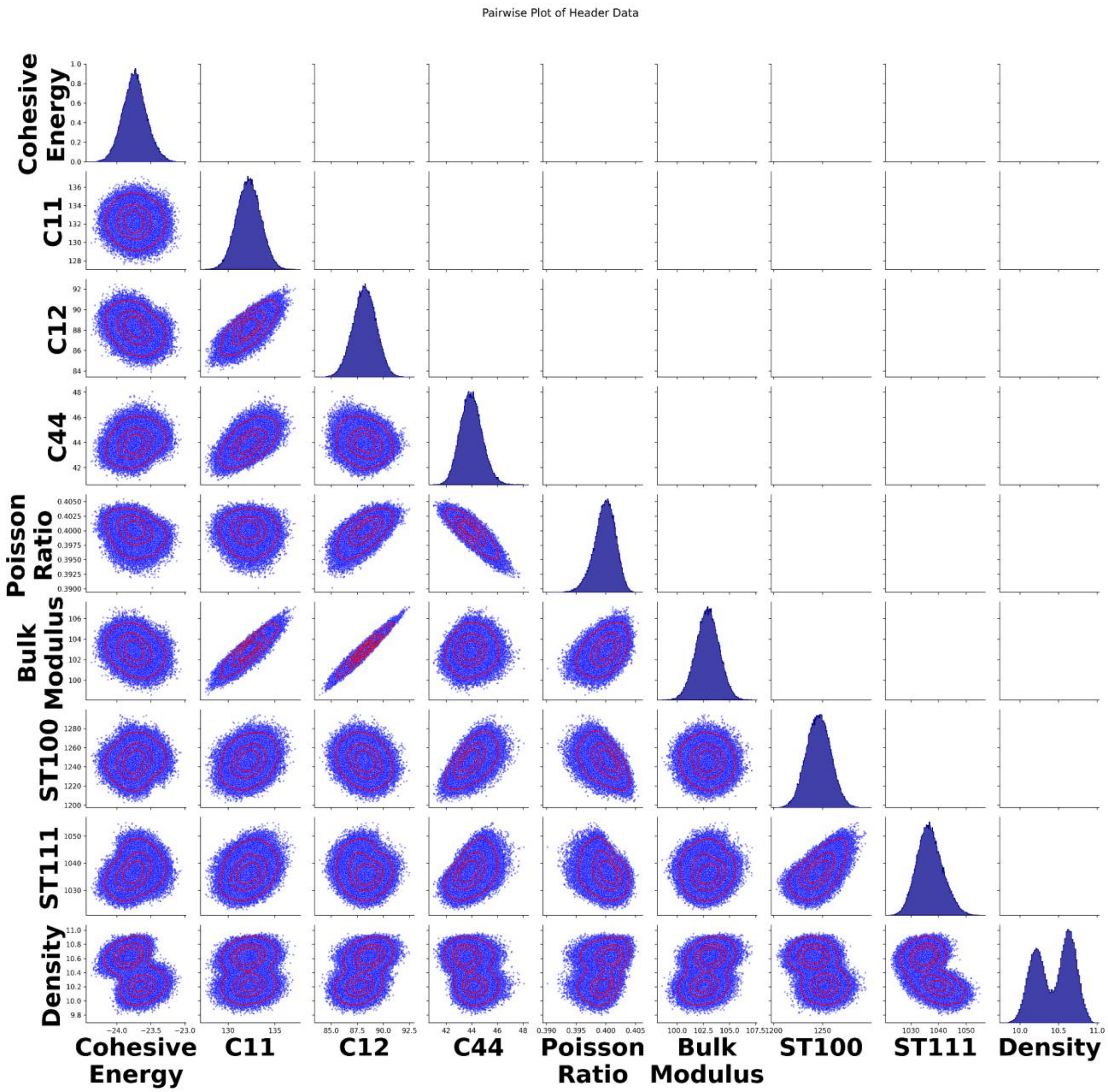

Figure S29. The corner plot of all properties for Ag predicted by GP on ESS estimated parameters. **Table S15** provides a catalogue of shapes observed in scattered plots including their corresponding symbols and names.

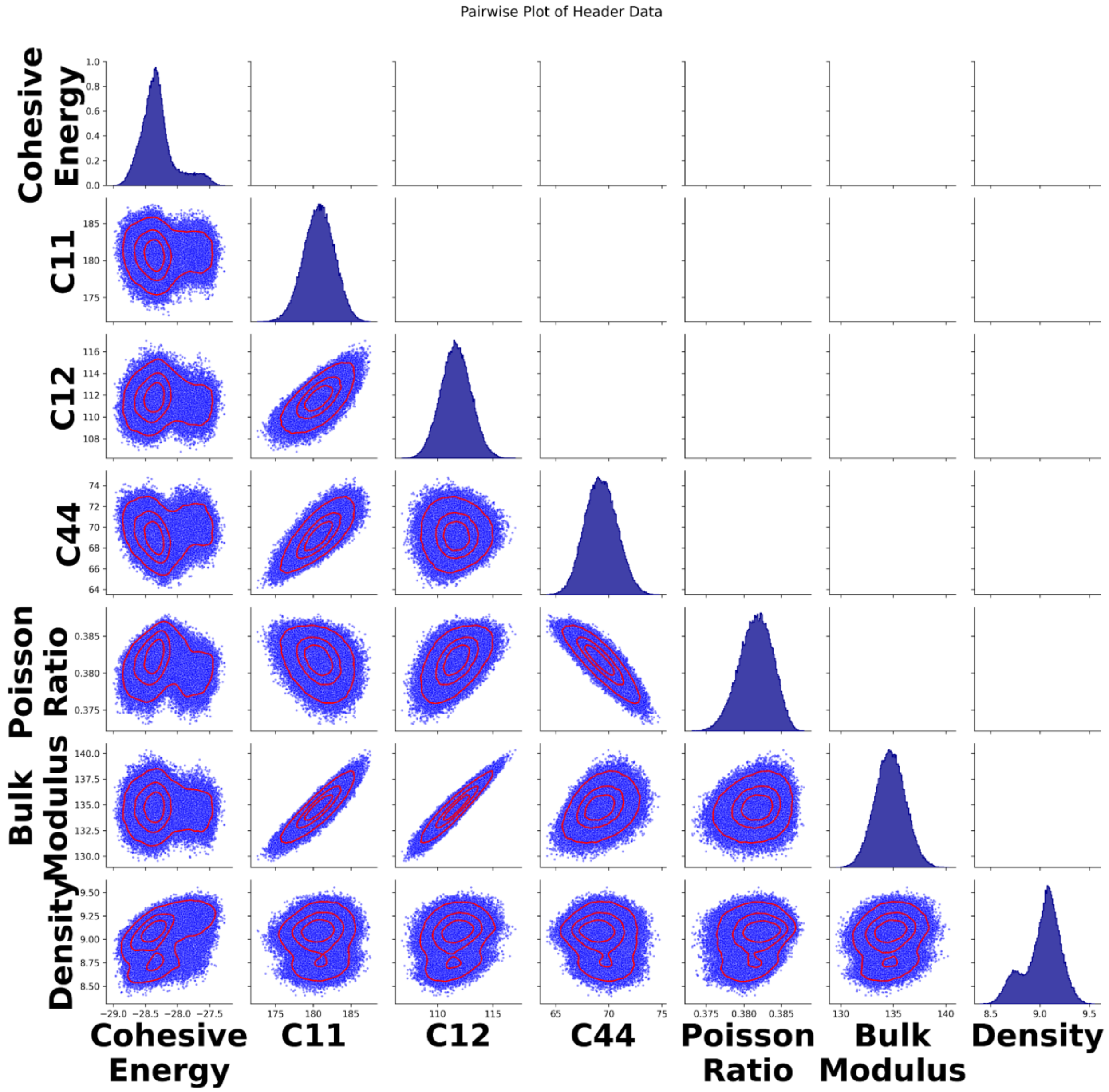

Figure S30. The corner plot of all properties for Cu predicted by GP on ESS estimated parameters. **Table S15** provides a catalogue of shapes observed in scattered plots including their corresponding symbols and names.

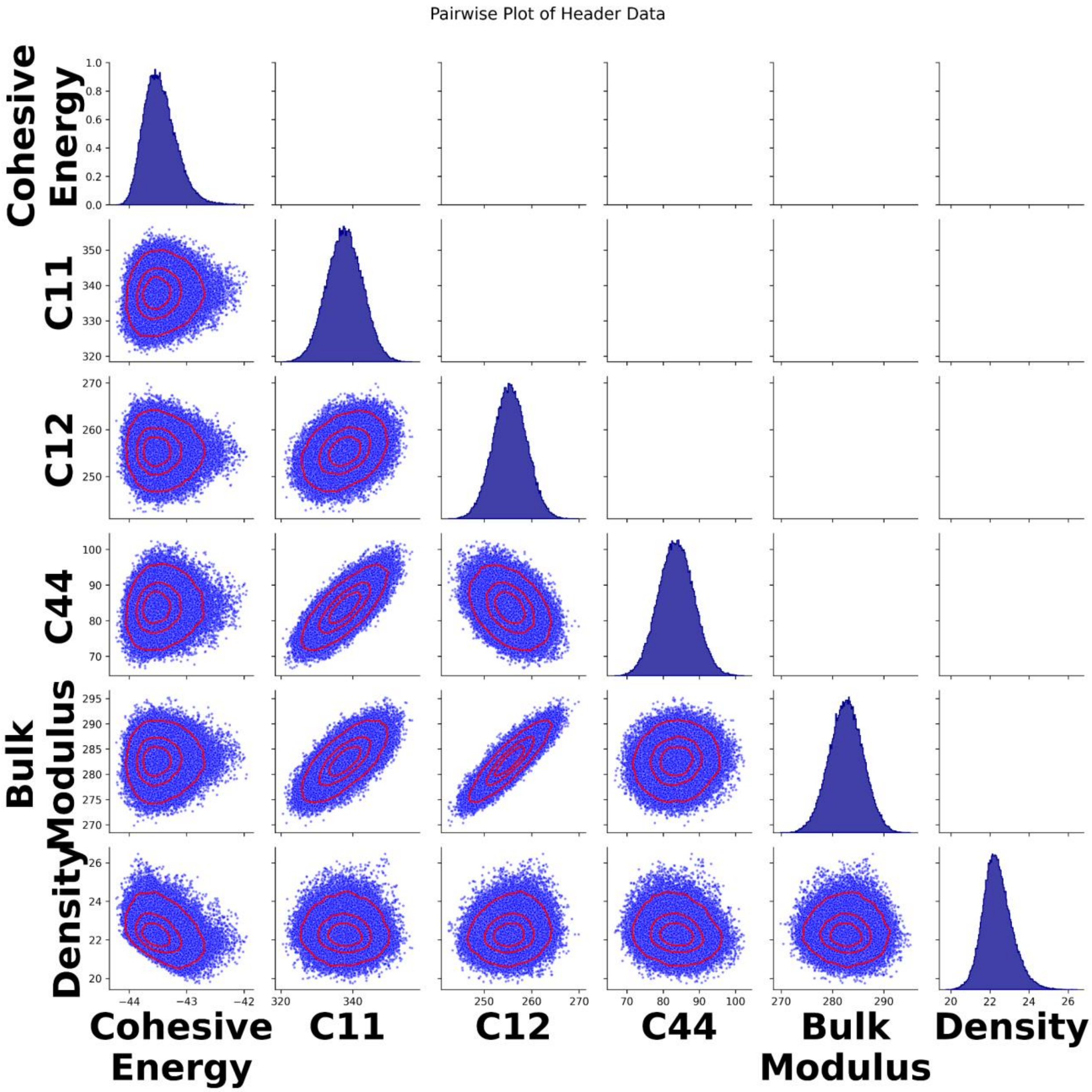

Figure S31. The corner plot of all properties for Pt predicted by GP on ESS estimated parameters.

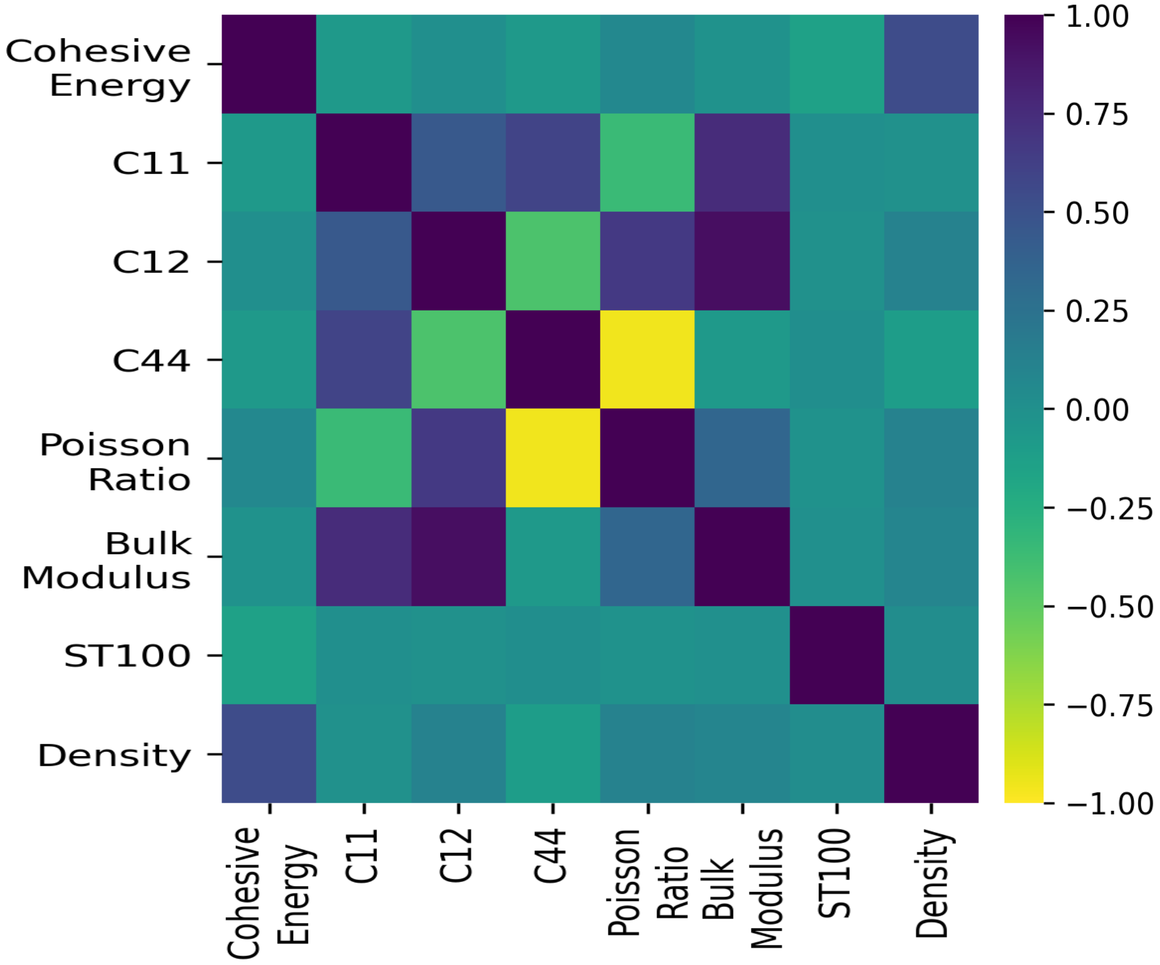

Figure S32. The heatmaps of Pearson correlation coefficients, illustrating the correlation between all properties, for Pd estimated by ESS sampler.

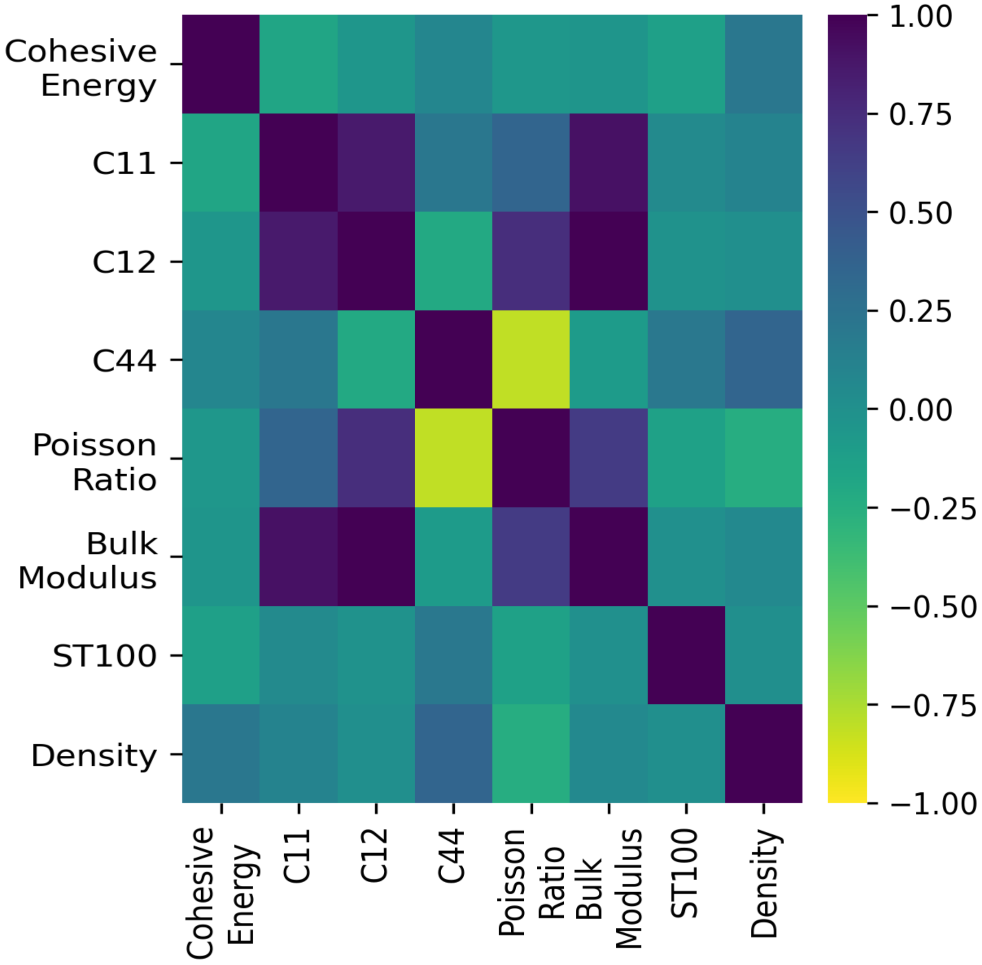

Figure S33. The heatmaps of Pearson correlation coefficients, illustrating the correlation between all properties, for Au estimated by ESS sampler.

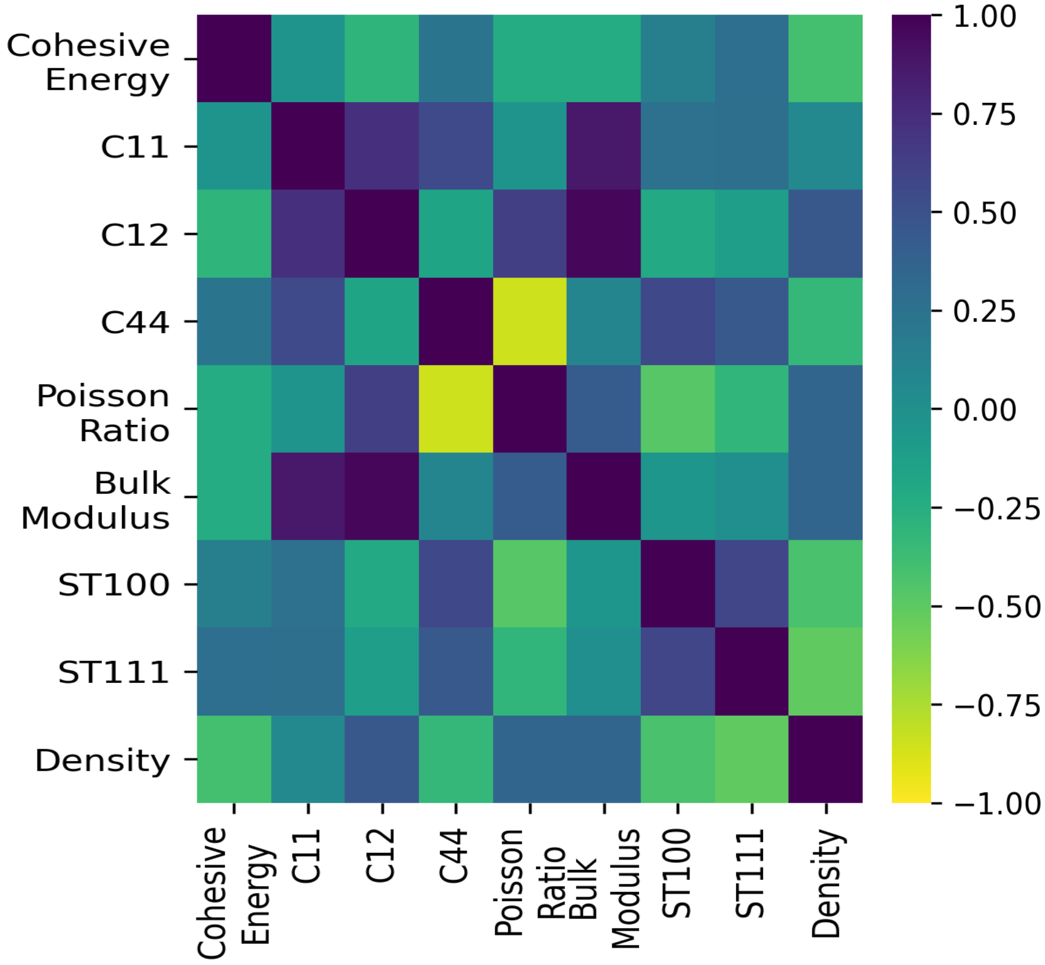

Figure S34. The heatmaps of Pearson correlation coefficients, illustrating the correlation between all properties, for Ag estimated by ESS sampler.

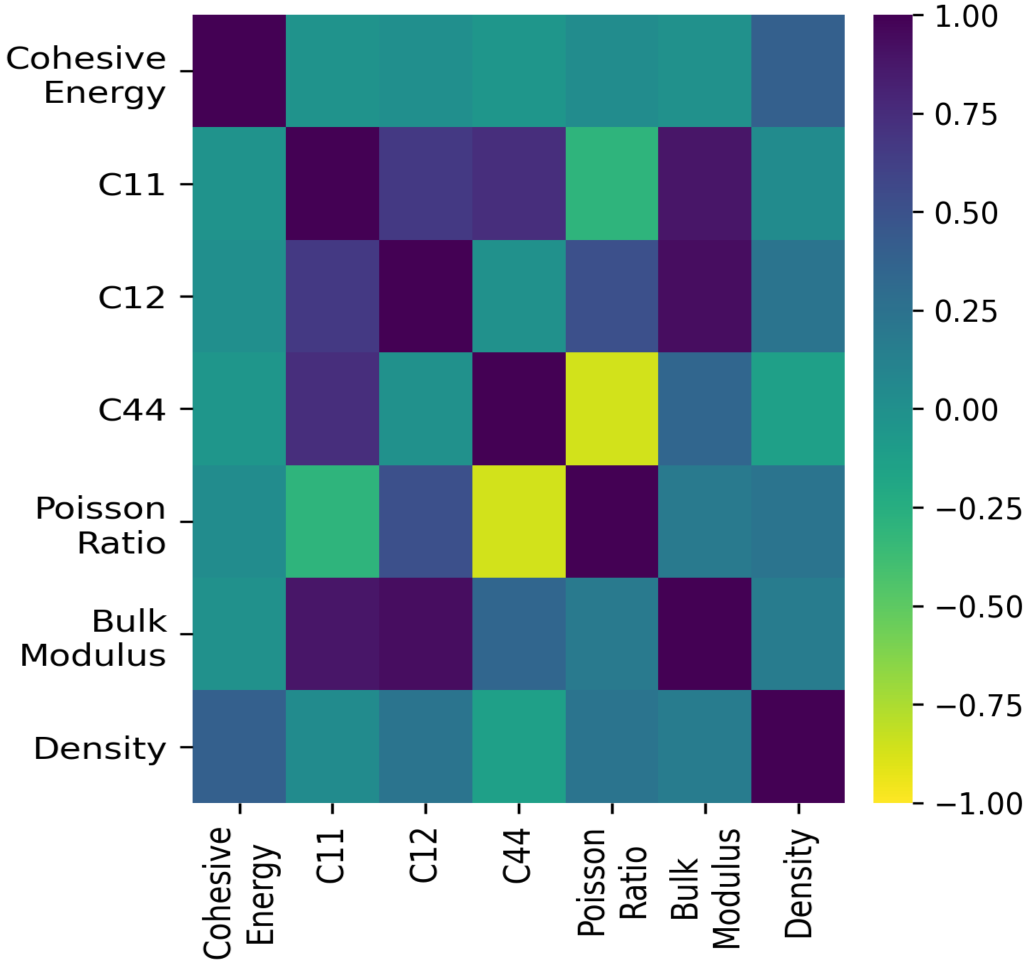

Figure S35. The heatmaps of Pearson correlation coefficients, illustrating the correlation between all properties, for Cu estimated by ESS sampler.

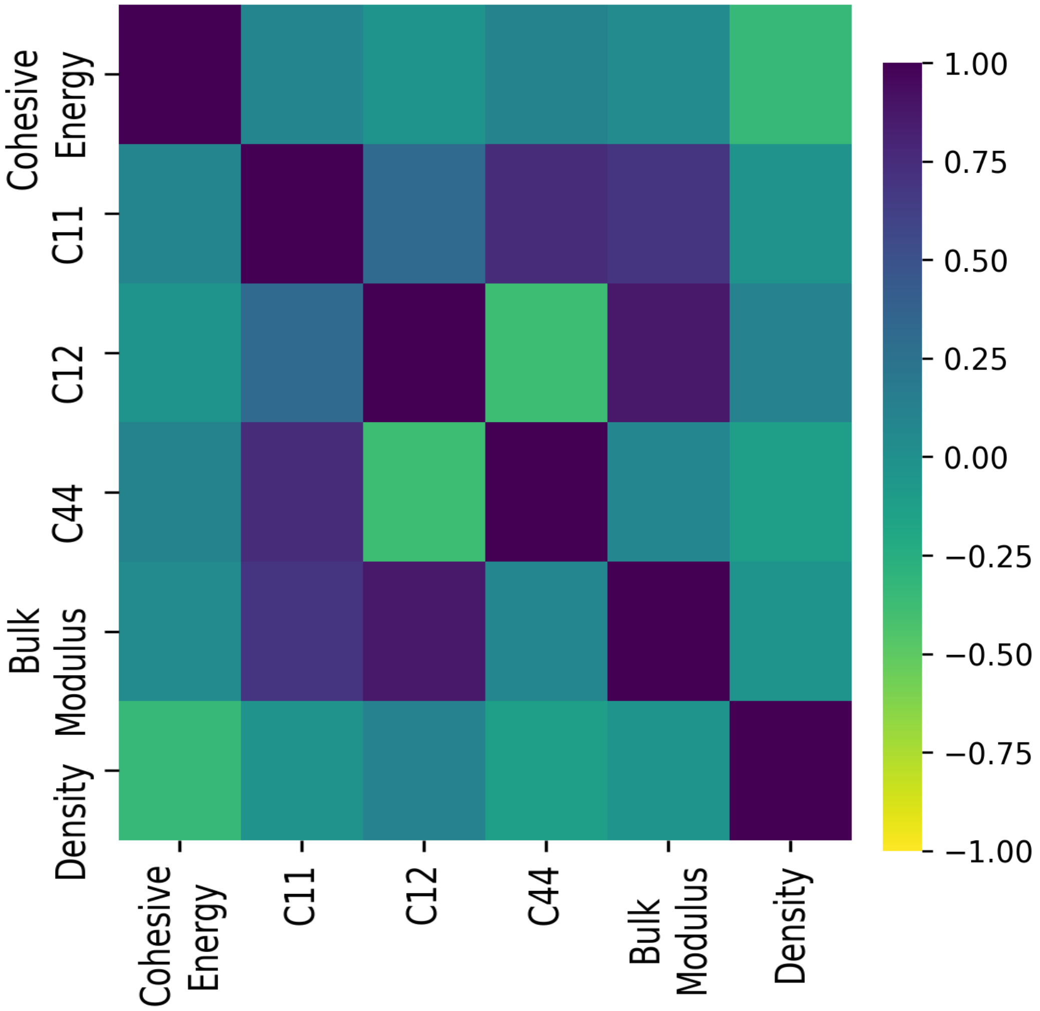

Figure S36. The heatmaps of Pearson correlation coefficients, illustrating the correlation between all properties, for Pt estimated by ESS sampler.

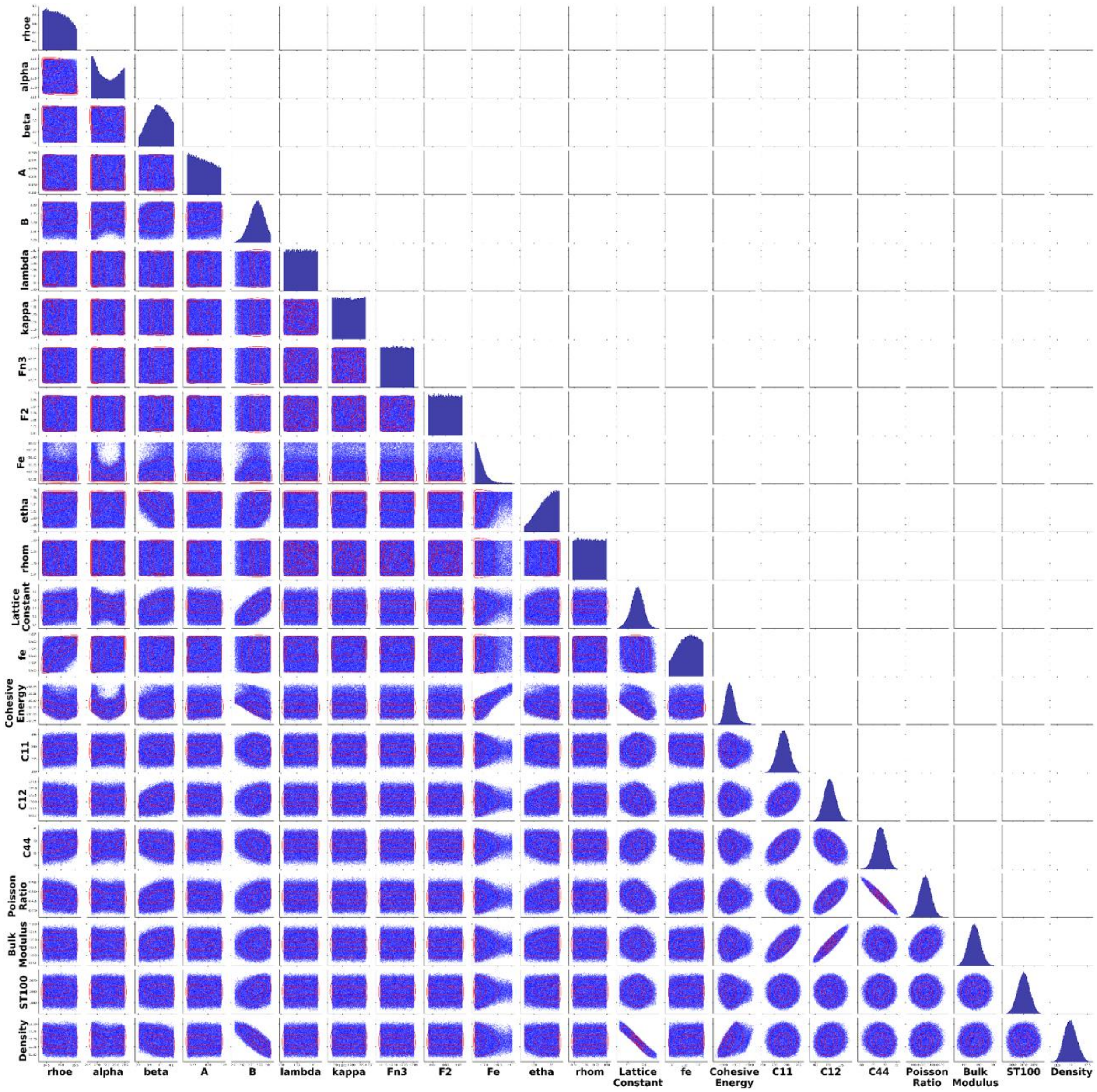

Figure S37. The corner plot of all ESS estimated parameters as well as properties predicted by GPR model on ESS estimated parameters for Pd. **Table S15** provides a catalogue of shapes observed in scattered plots including their corresponding symbols and names.

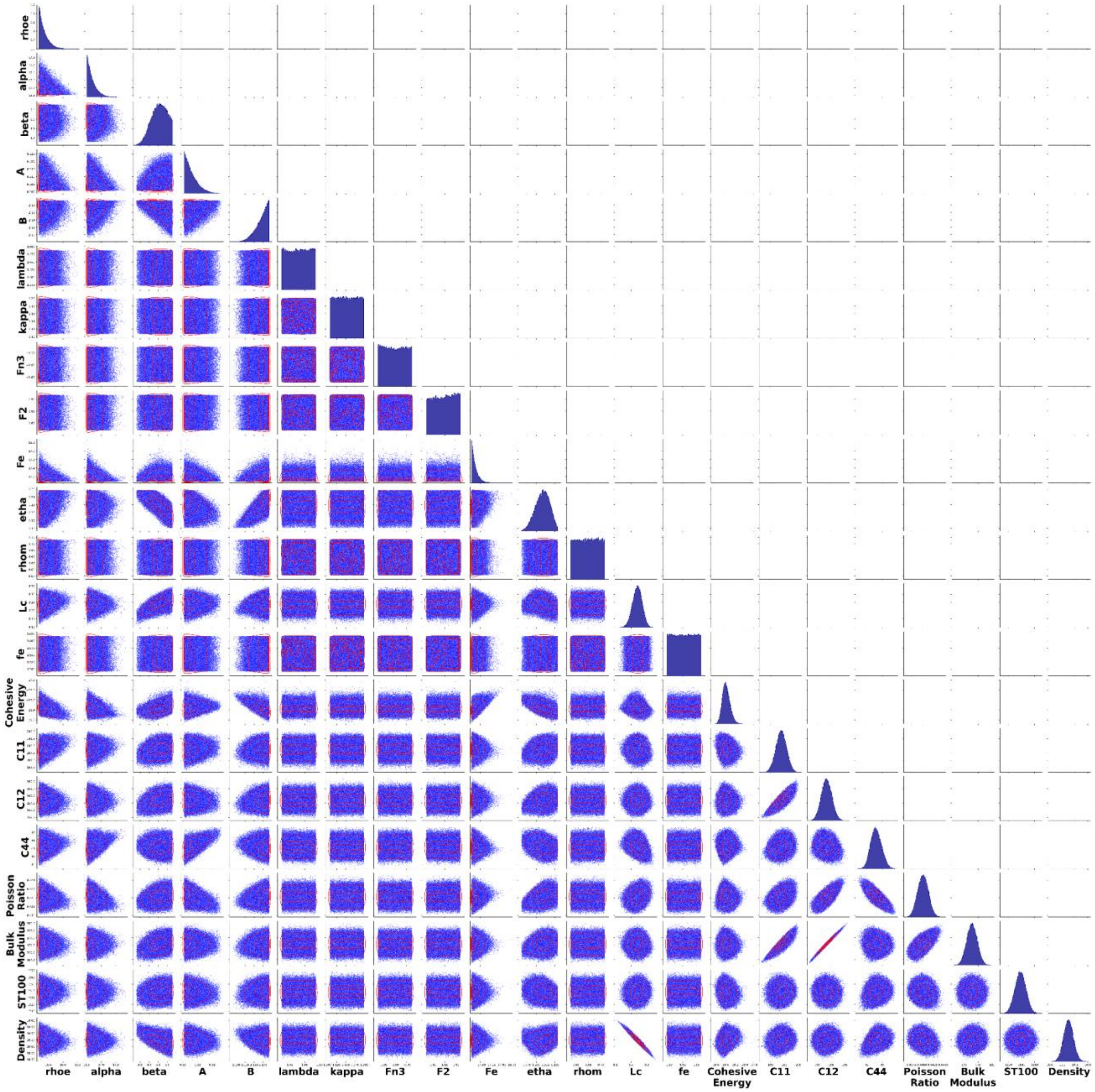

Figure S38. The corner plot of all ESS estimated parameters as well as properties predicted by GPR model on ESS estimated parameters for Au. **Table S15** provides a catalogue of shapes observed in scattered plots including their corresponding symbols and names.

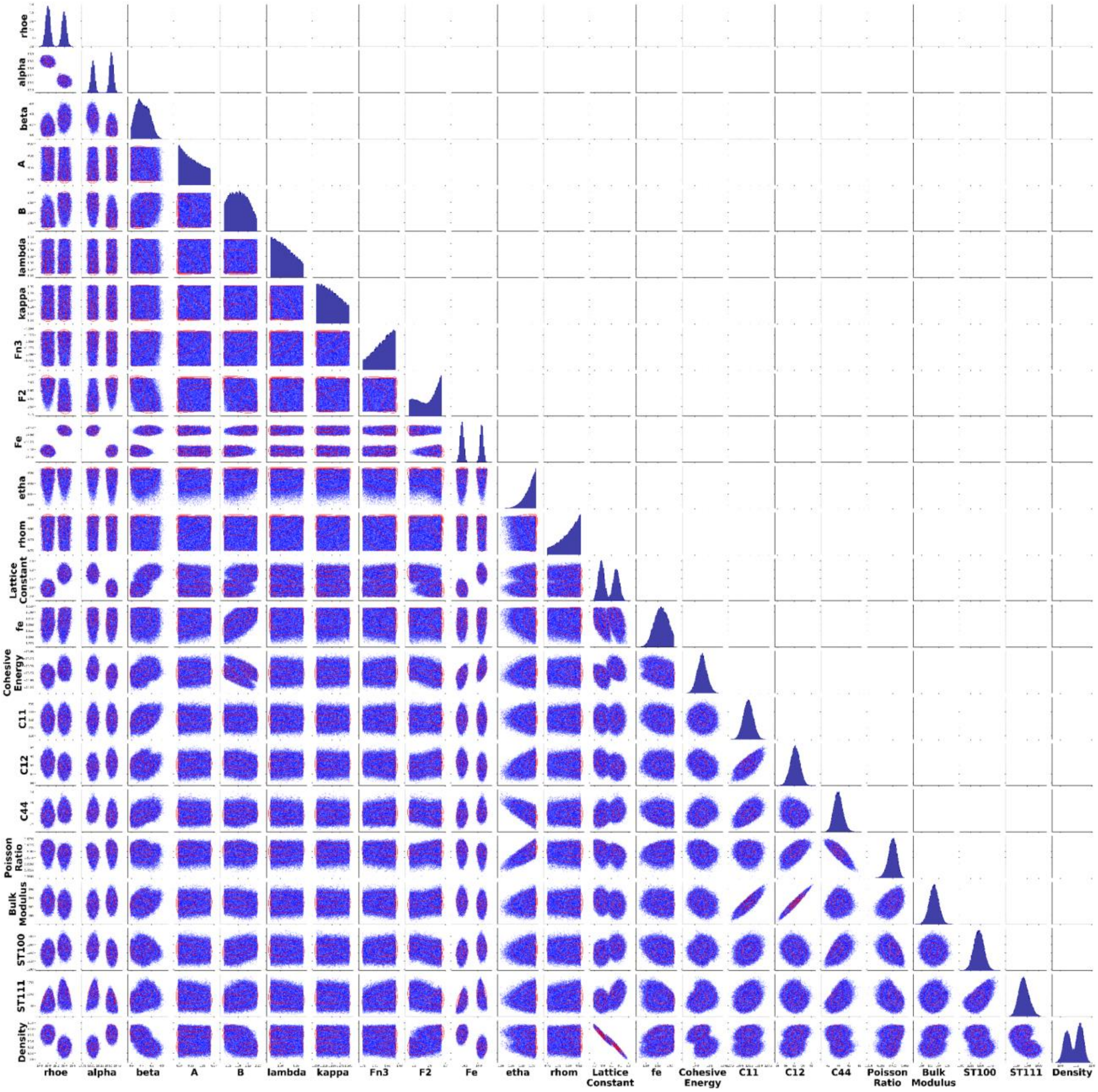

Figure S39. The corner plot of all ESS estimated parameters as well as properties predicted by GPR model on ESS estimated parameters for Ag. **Table S15** provides a catalogue of shapes observed in scattered plots including their corresponding symbols and names.

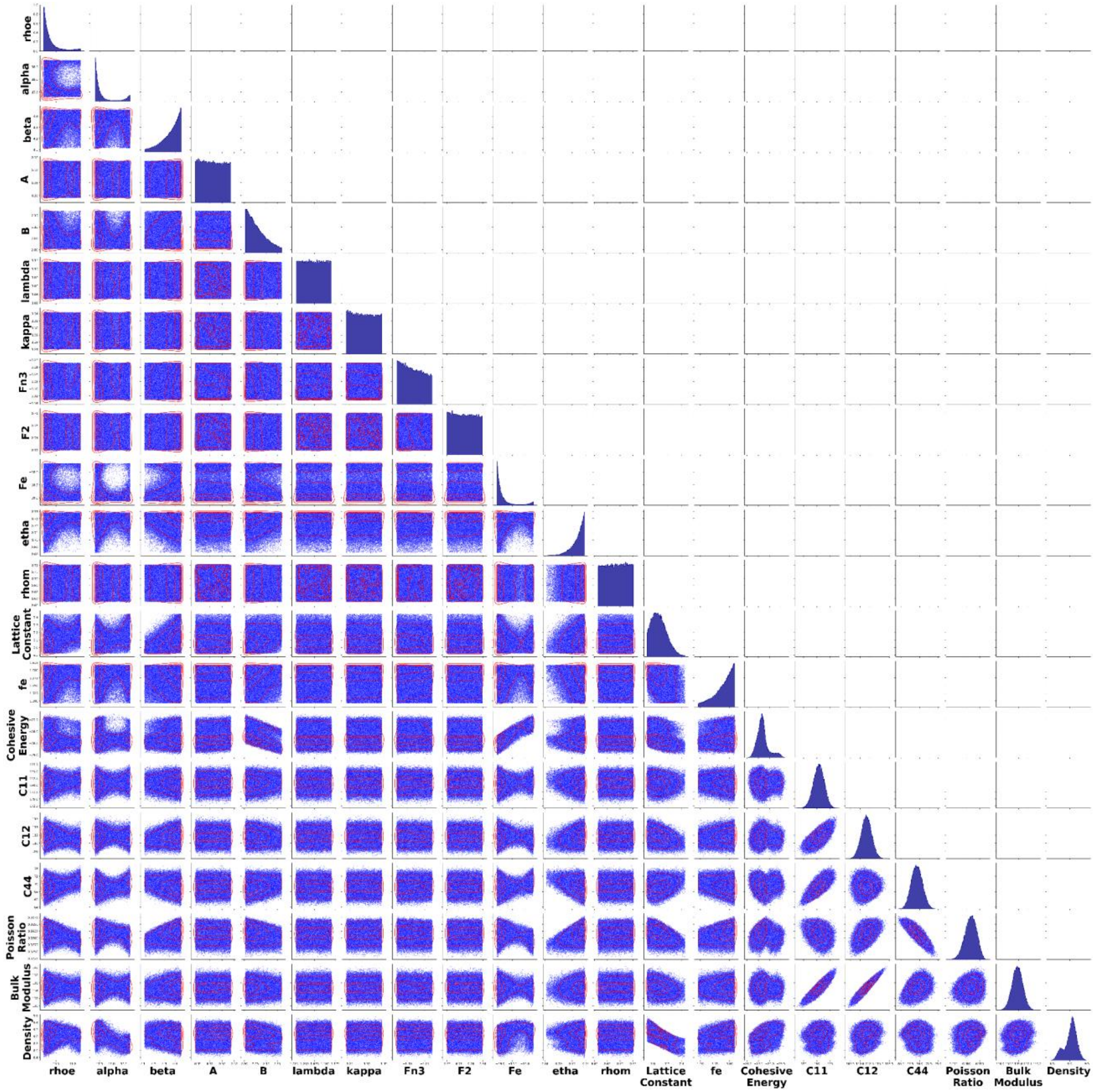

Figure S40. The corner plot of all ESS estimated parameters as well as properties predicted by GPR model on ESS estimated parameters for Cu. **Table S15** provides a catalogue of shapes observed in scattered plots including their corresponding symbols and names.

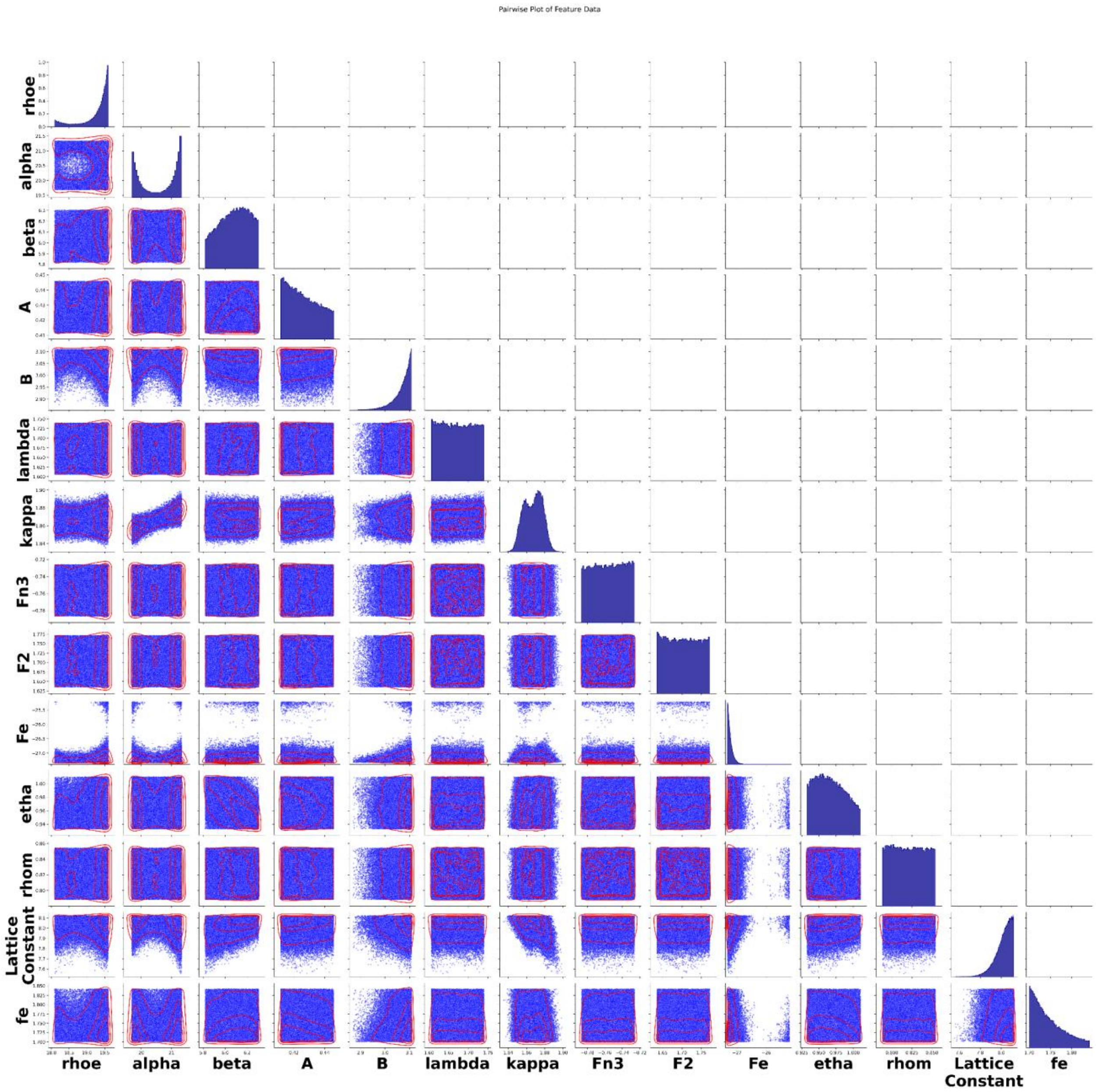

Figure S41. The corner plot of all ESS estimated parameters as well as properties predicted by GPR model on ESS estimated parameters for Pt. **Table S15** provides a catalogue of shapes observed in scattered plots including their corresponding symbols and names.

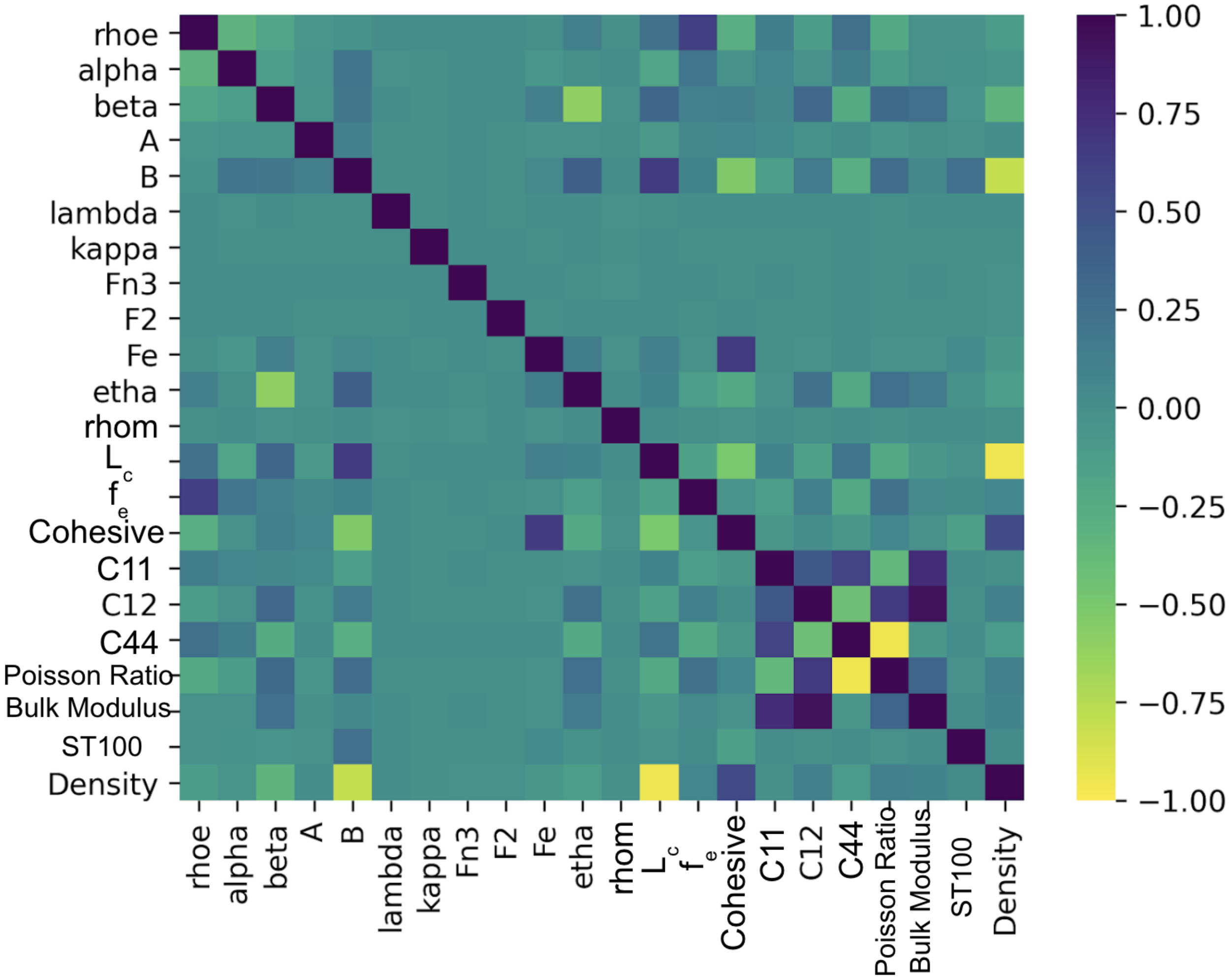

Figure S42. The heatmaps of Pearson correlation coefficients, illustrating the correlation between all ESS estimated parameters as well as properties predicted by GPR model on ESS estimated parameters, for Pd estimated by ESS sampler.

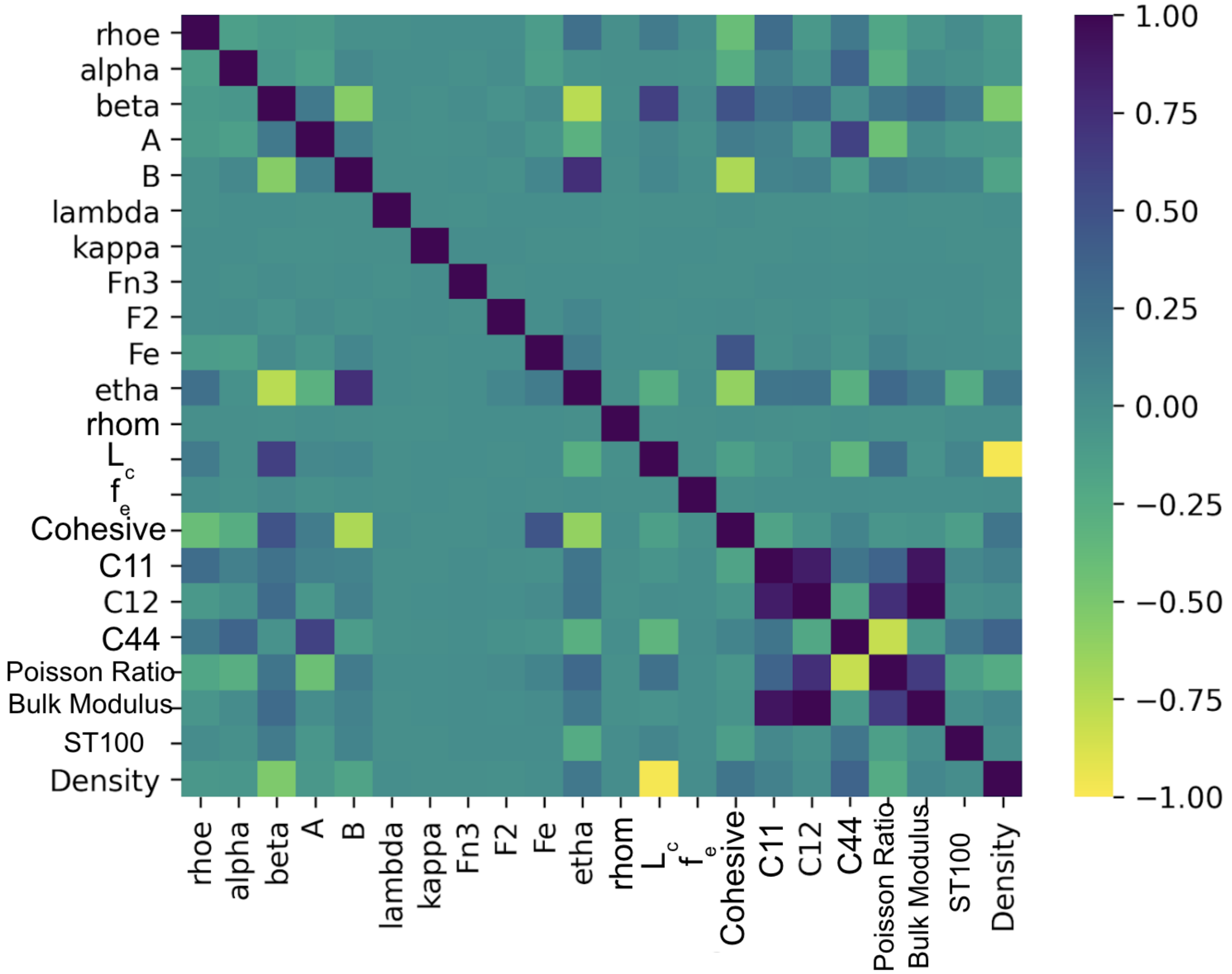

Figure S43. The heatmaps of Pearson correlation coefficients, illustrating the correlation between all ESS estimated parameters as well as properties predicted by GPR model on ESS estimated parameters, for Au estimated by ESS sampler.

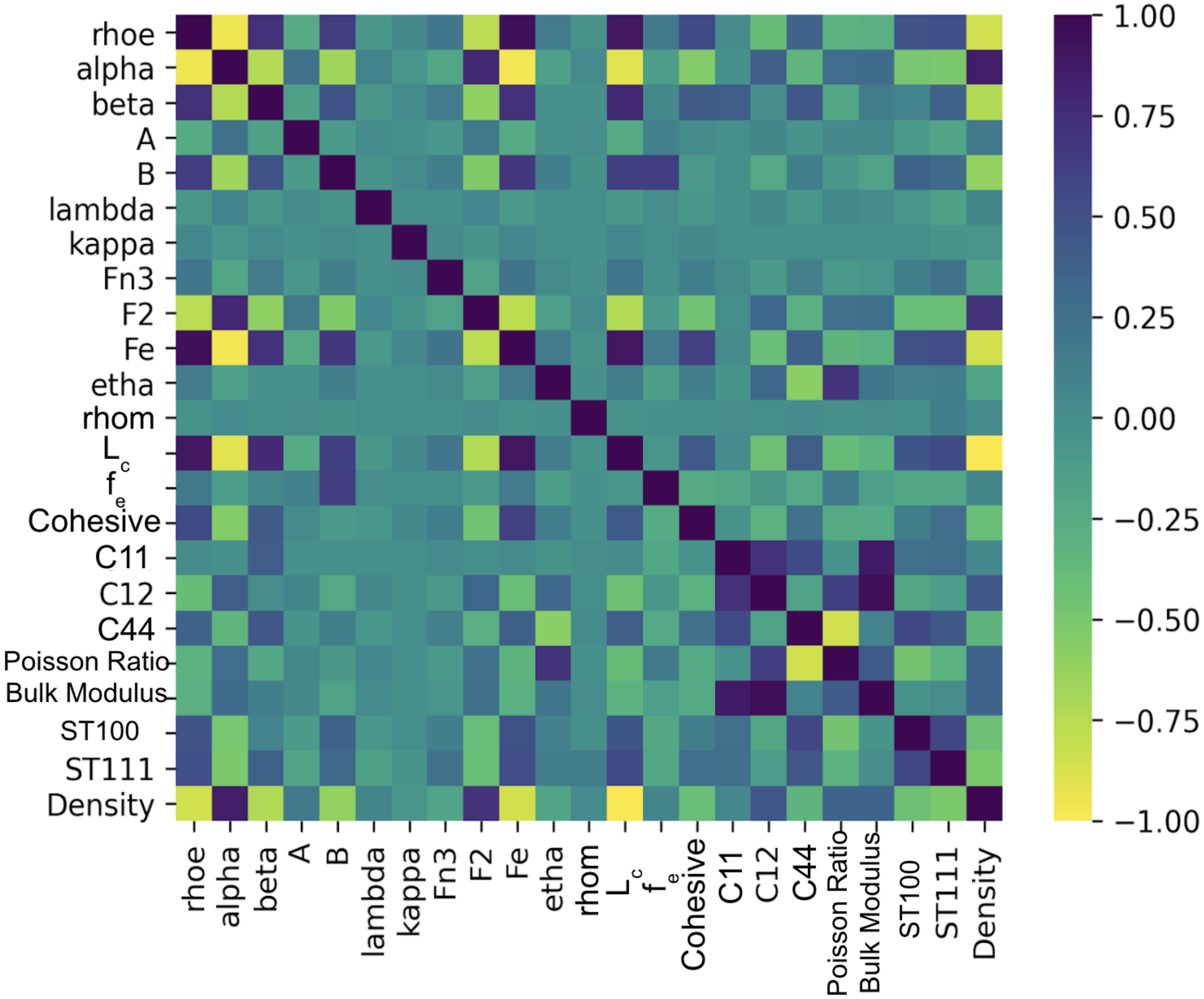

Figure S44. The heatmaps of Pearson correlation coefficients, illustrating the correlation between all ESS estimated parameters as well as properties predicted by GPR model on ESS estimated parameters, for Ag estimated by ESS sampler.

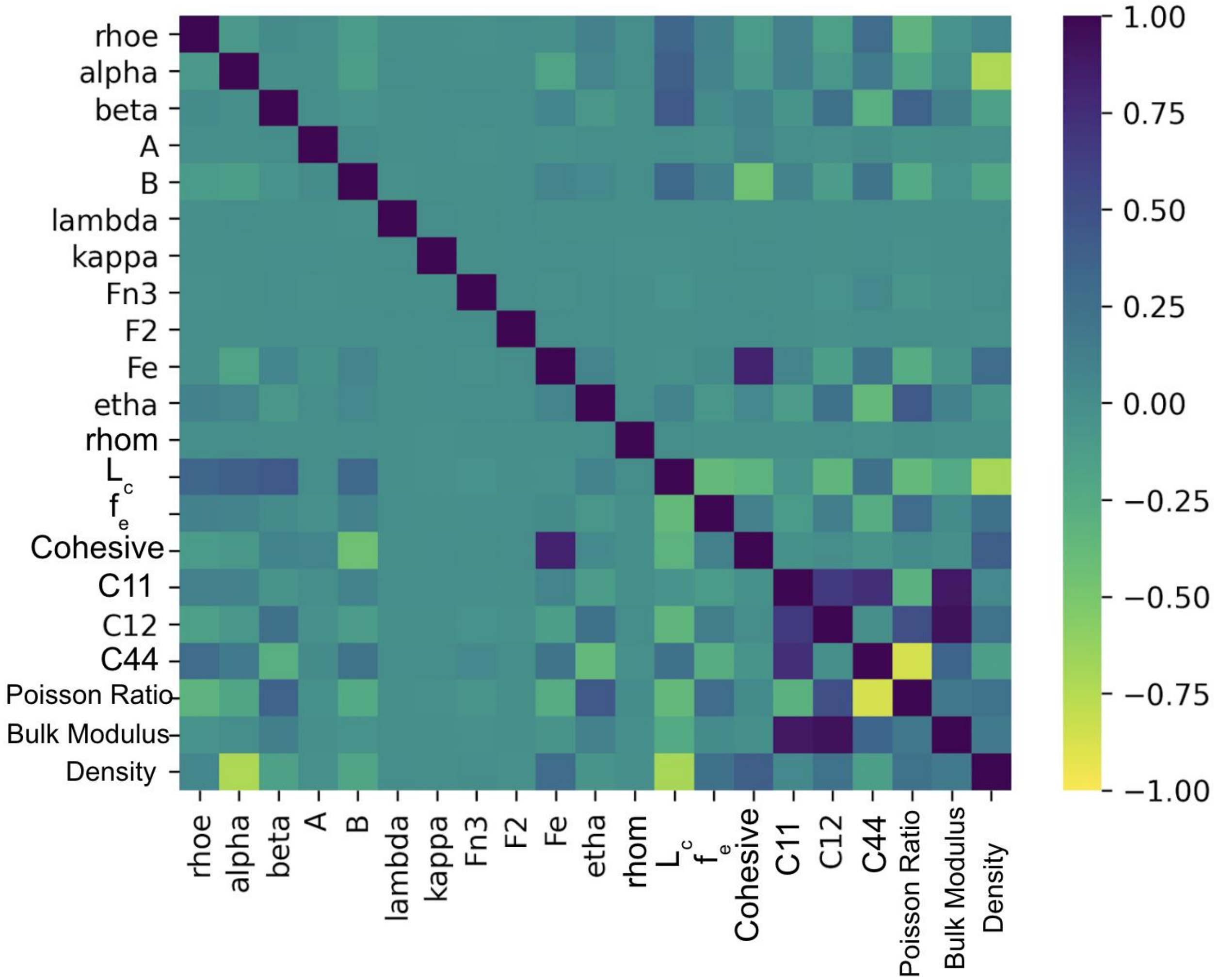

Figure S45. The heatmaps of Pearson correlation coefficients, illustrating the correlation between all ESS estimated parameters as well as properties predicted by GPR model on ESS estimated parameters, for Cu estimated by ESS sampler.

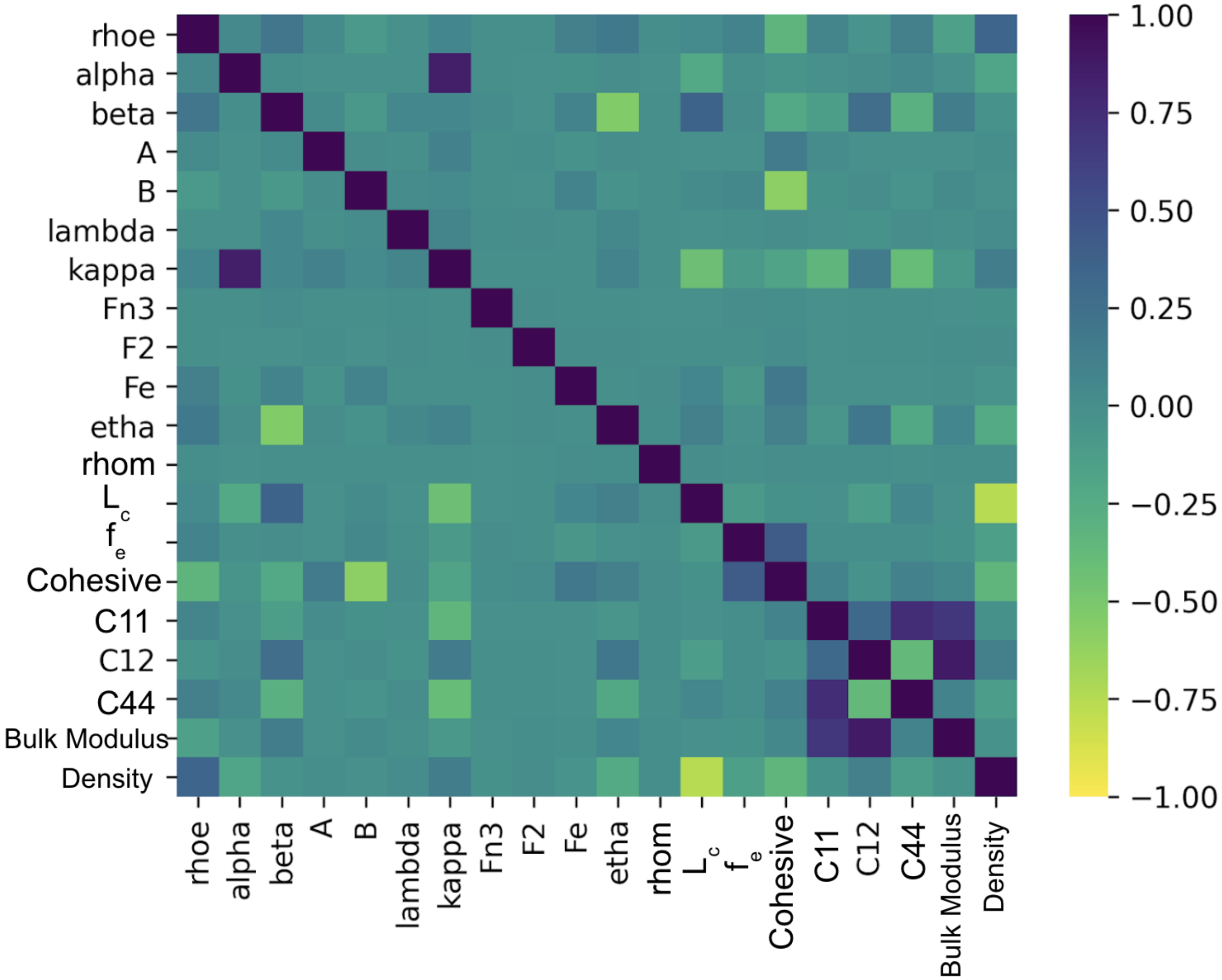

Figure S46. The heatmaps of Pearson correlation coefficients, illustrating the correlation between all ESS estimated parameters as well as properties predicted by GPR model on ESS estimated parameters, for Pt estimated by ESS sampler.

Table S15. A tabular representation of all the symbols and names used to denote different types of scatter plots of BUQ.

Symbol	Name	Representative Figure
D	Dumbbell	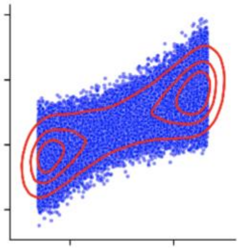

T	Triangle	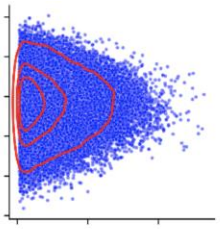
C	Circle	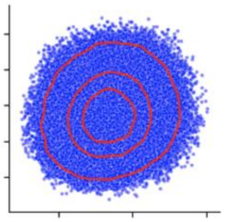
U	U-shape	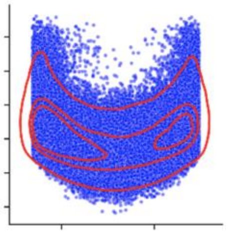
VB	Vertical stripes	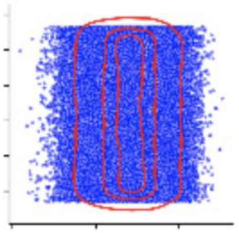
HB	Horizontal stripes	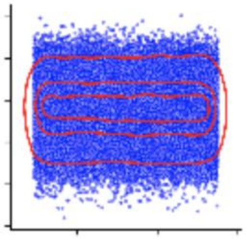

S4.3 95% Credible Intervals - Posterior Distribution for input parameters

Table S16. A comparison of values defining the 95% credible intervals for parameters obtained for Pd. The posterior distribution mean represents the average parameter values obtained after Bayesian parameter estimation. The lower and upper limits define the 95% credible interval for the posterior distribution. PSO refers to the parameter values obtained from the original optimization using the PSO algorithm. The "negative %" and "positive %" indicate the percentage difference between the lower and upper credible interval limits relative to the PSO value. A "-" in

the “negative %” column or “positive % column” indicates that the PSO value was outside of the credible intervals .

Pd	Posterior distribution Mean	lower credible limit	upper credible limit	PSO	Negative %	Positive %
ρ_e	14.916	14.256	15.576	14.96	4.71	4.12
α	22.52	21.388	23.652	22.581	5.28	4.74
β	3.977	3.819	4.135	3.963	3.63	4.34
A	0.277	0.265	0.289	0.278	4.68	3.96
B	2.732	2.65	2.814	2.71	2.21	3.84
λ	1.363	1.301	1.425	1.363	4.55	4.55
κ	1.193	1.137	1.249	1.193	4.69	4.69
F_{n3}	-2.08	-2.176	-1.984	-2.08	4.62	4.62
F_2	0.66	0.63	0.69	0.66	4.55	4.55
F_e	-17.085	-17.515	-16.655	-16.652	5.18	-
η	1.337	1.285	1.389	1.324	2.95	4.91
ρ_m	0.769	0.733	0.805	0.769	4.68	4.68
Lattice constant	7.714	7.566	7.862	7.711	1.88	1.96
f_e	1.562	1.496	1.628	1.555	3.79	4.69

Table S17. A comparison of values defining the 95% credible intervals for parameters obtained for Au. The posterior distribution mean represents the average parameter values obtained after Bayesian parameter estimation. The lower and upper limits define the 95% credible interval for the posterior distribution. PSO refers to the parameter values obtained from the original optimization using the PSO algorithm. The “negative %” and “positive %” indicate the percentage difference between the lower and upper credible interval limits relative to the PSO value. A “-” in the “negative %” column or “positive % column” indicates that the PSO value was outside of the credible intervals .

Au	Posterior distribution Mean	lower credible limit	upper credible limit	PSO	Negative %	Positive %
ρ_e	16.344	16.21	16.478	16.946	4.34	-

α	16.878	16.746	17.01	17.505	4.34	-
β	5.009	4.841	5.177	4.957	2.34	4.44
A	0.306	0.298	0.314	0.313	4.79	0.32
B	2.17	2.136	2.204	2.108	-	4.55
λ	1.431	1.365	1.497	1.43	4.55	4.69
κ	1.153	1.099	1.207	1.153	4.68	4.68
F_{n3}	-1.798	-1.882	-1.714	-1.797	4.73	4.62
F_2	1.846	1.76	1.932	1.844	4.56	4.77
F_e	-17.627	-17.789	-17.465	-17.034	4.43	-
η	1.217	1.179	1.255	1.209	2.48	3.8
ρ_m	0.679	0.647	0.711	0.679	4.71	4.71
Lattice constant	8.239	8.179	8.299	8.16	-	1.7
f_e	1.555	1.483	1.627	1.555	4.63	4.63

Table S18. A comparison of values defining the 95% credible intervals for parameters obtained for Ag, Mode 1. The posterior distribution mean represents the average parameter values obtained after Bayesian parameter estimation. The lower and upper limits define the 95% credible interval for the posterior distribution. PSO refers to the parameter values obtained from the original optimization using the PSO algorithm. The "negative %" and "positive %" indicate the percentage difference between the lower and upper credible interval limits relative to the PSO value. A "-" in the "negative %" column or "positive % column" indicates that the PSO value was outside of the credible intervals .

**Ag**	**Mode 1**					
	**Posterior distribution Mean**	**lower credible limit**	**upper credible limit**	**PSO**	**Negative %**	**Positive %**
ρ_e	15.393	15.289	15.497	15.17	-	2.16
α	18.049	17.941	18.157	18.331	2.13	-
β	4.764	4.674	4.854	4.783	2.28	1.48
A	0.349	0.335	0.363	0.353	5.1	2.83
B	2.138	2.072	2.204	2.112	1.89	4.36

λ	1.309	1.253	1.365	1.32	5.08	3.41
κ	1.248	1.192	1.304	1.253	4.87	4.07
F_{n3}	-1.656	-1.722	-1.59	-1.679	2.56	5.3
F_2	2.533	2.445	2.621	2.573	4.97	1.87
F_e	-13.865	-13.973	-13.757	-14.221	-	3.26
η	0.896	0.876	0.916	0.873	-	4.93
ρ_m	0.8	0.766	0.834	0.792	3.28	5.3
Lattice constant	8.158	8.086	8.23	8.127	0.5	1.27
f_e	1.868	1.814	1.922	1.844	1.63	4.23

Table S19. A comparison of values defining the 95% credible intervals for parameters obtained for Ag, Mode 2. The posterior distribution mean represents the average parameter values obtained after Bayesian parameter estimation. The lower and upper limits define the 95% credible interval for the posterior distribution. PSO refers to the parameter values obtained from the original optimization using the PSO algorithm. The "negative %" and "positive %" indicate the percentage difference between the lower and upper credible interval limits relative to the PSO value. A "-" in the "negative %" column or "positive % column" indicates that the PSO value was outside of the credible intervals .

**Ag**	**Mode 2**					
	**Posterior distribution Mean**	**lower credible limit**	**upper credible limit**	**PSO**	**Negative %**	**Positive %**
ρ_e	14.999	14.891	15.107	15.17	1.84	-
α	18.584	18.482	18.686	18.331	-	1.94
β	4.67	4.592	4.748	4.783	3.99	-0.73
A	0.353	0.337	0.369	0.353	4.53	4.53
B	2.078	2.016	2.14	2.112	4.55	1.33
λ	1.314	1.256	1.372	1.32	4.85	3.94
κ	1.245	1.191	1.299	1.253	4.95	3.67
F_{n3}	-1.671	-1.743	-1.599	-1.679	3.81	4.76
F_2	2.635	2.569	2.701	2.573	0.16	4.97

F_e	-14.562	-14.684	-14.44	-14.221	3.26	-
η	0.893	0.869	0.917	0.873	0.46	5.04
ρ_m	0.801	0.767	0.835	0.792	3.16	5.43
Lattice constant	7.983	7.913	8.053	8.127	2.63	-
f_e	1.86	1.808	1.912	1.844	1.95	3.69

Table S20. A comparison of values defining the 95% credible intervals for parameters obtained for Cu, Mode 1. The posterior distribution mean represents the average parameter values obtained after Bayesian parameter estimation. The lower and upper limits define the 95% credible interval for the posterior distribution. PSO refers to the parameter values obtained from the original optimization using the PSO algorithm. The "negative %" and "positive %" indicate the percentage difference between the lower and upper credible interval limits relative to the PSO value. A "-" in the "negative %" column or "positive % column" indicates that the PSO value was outside of the credible intervals .

**Cu**	**Mode 1**					
	**Posterior distribution Mean**	**lower credible limit**	**upper credible limit**	**PSO**	**Negative %**	**Positive %**
ρ_e	13.323	12.867	13.779	13.684	5.97	0.69
α	18.552	18.196	18.908	18.035	-	4.84
β	4.355	4.185	4.525	4.286	2.36	5.58
A	0.363	0.347	0.379	0.363	4.41	4.41
B	2.052	1.976	2.128	2.087	5.32	1.96
λ	1.073	1.023	1.123	1.073	4.66	4.66
κ	1.291	1.231	1.351	1.292	4.72	4.57
F_{n3}	-1.283	-1.341	-1.225	-1.279	4.85	4.22
F_2	0.75	0.716	0.784	0.75	4.53	4.53
F_e	-18.966	-19.438	-18.494	-18.399	5.65	-
η	0.729	0.711	0.747	0.71	-	5.21
ρ_m	0.693	0.661	0.725	0.693	4.62	4.62

Lattice constant	7.182	6.97	7.394	7.2	3.19	2.69
f_e	1.583	1.525	1.641	1.555	1.93	5.53

Table S21. A comparison of values defining the 95% credible intervals for parameters obtained for Cu, Mode 2. The posterior distribution mean represents the average parameter values obtained after Bayesian parameter estimation. The lower and upper limits define the 95% credible interval for the posterior distribution. PSO refers to the parameter values obtained from the original optimization using the PSO algorithm. The "negative %" and "positive %" indicate the percentage difference between the lower and upper credible interval limits relative to the PSO value. A "-" in the "negative %" column or "positive % column" indicates that the PSO value was outside of the credible intervals .

Cu	Mode 2					
	**Posterior distribution Mean**	**lower credible limit**	**upper credible limit**	**PSO**	**Negative %**	**Positive %**
ρ_e	13.366	12.844	13.888	13.684	6.14	1.49
α	17.472	17.15	17.794	18.035	4.91	-
β	4.356	4.194	4.518	4.286	2.15	5.41
A	0.363	0.347	0.379	0.363	4.41	4.41
B	2.062	1.978	2.146	2.087	5.22	2.83
λ	1.073	1.023	1.123	1.073	4.66	4.66
κ	1.291	1.231	1.351	1.292	4.72	4.57
F_{n3}	-1.283	-1.341	-1.225	-1.279	4.85	4.22
F_2	0.749	0.715	0.783	0.75	4.67	4.4
F_e	-18.833	-19.611	-18.055	-18.399	6.59	1.87
η	0.728	0.708	0.748	0.71	0.28	5.35
ρ_m	0.693	0.661	0.725	0.693	4.62	4.62
Lattice constant	7.074	6.888	7.26	7.2	4.33	0.83
f_e	1.577	1.515	1.639	1.555	2.57	5.4

Table S22. A comparison of values defining the 95% credible intervals for parameters obtained for Pt, Mode 1. The posterior distribution mean represents the average parameter values obtained

after Bayesian parameter estimation. The lower and upper limits define the 95% credible interval for the posterior distribution. PSO refers to the parameter values obtained from the original optimization using the PSO algorithm. The "negative %" and "positive %" indicate the percentage difference between the lower and upper credible interval limits relative to the PSO value. A "-" in the "negative %" column or "positive % column" indicates that the PSO value was outside of the credible intervals.

Pt	Mode 1					
	**Posterior distribution Mean**	**lower credible limit**	**upper credible limit**	**PSO**	**Negative %**	**Positive %**
ρ_e	19.255	18.469	20.041	18.845	2	6.35
α	21.101	20.689	21.513	20.509	-	4.9
β	6.081	5.823	6.339	6.063	3.96	4.55
A	0.426	0.406	0.446	0.429	5.36	3.96
B	3.069	2.995	3.143	2.989	-	5.15
λ	1.671	1.593	1.749	1.673	4.78	4.54
κ	1.874	1.862	1.886	1.877	0.8	0.48
F_{n3}	-0.756	-0.79	-0.722	-0.756	4.5	4.5
F_2	1.702	1.622	1.782	1.703	4.76	4.64
F_e	-27.209	-27.501	-26.917	-26.266	4.7	-
η	0.968	0.926	1.01	0.972	4.73	3.91
ρ_m	0.821	0.783	0.859	0.821	4.63	4.63
Lattice constant	8.009	7.845	8.173	7.819	-	4.53
f_e	1.746	1.674	1.818	1.771	5.48	2.65

Table S23. A comparison of values defining the 95% credible intervals for parameters obtained for Pt, Mode 2. The posterior distribution mean represents the average parameter values obtained after Bayesian parameter estimation. The lower and upper limits define the 95% credible interval for the posterior distribution. PSO refers to the parameter values obtained from the original optimization using the PSO algorithm. The "negative %" and "positive %" indicate the percentage difference between the lower and upper credible interval limits relative to the PSO value. A "-" in the "negative %" column or "positive % column" indicates that the PSO value was outside of the credible intervals .

Pt	Mode 2					
	**Posterior distribution Mean**	**lower credible limit**	**upper credible limit**	**PSO**	**Negative %**	**Positive %**
ρ_e	19.22	18.374	20.066	18.845	2.5	6.48
α	19.925	19.505	20.345	20.509	4.9	-
β	6.078	5.812	6.344	6.063	4.14	4.63
A	0.427	0.407	0.447	0.429	5.13	4.2
B	3.07	2.998	3.142	2.989	-	5.12
λ	1.673	1.595	1.751	1.673	4.66	4.66
κ	1.858	1.846	1.87	1.877	1.65	-
F_{n3}	-0.756	-0.792	-0.72	-0.756	4.76	4.76
F_2	1.703	1.623	1.783	1.703	4.7	4.7
F_e	-27.2	-27.586	-26.814	-26.266	5.03	-
η	0.967	0.925	1.009	0.972	4.84	3.81
ρ_m	0.821	0.783	0.859	0.821	4.63	4.63
Lattice constant	8.038	7.912	8.164	7.819	-	4.41
f_e	1.746	1.674	1.818	1.771	5.48	2.65

Section S4.4 95% Credible Intervals - Posterior Distribution for output properties.

Table S24: A comparison of values defining 95 % credible intervals for properties obtained for Pd metal.

Pd	Posterior Mean	2 Std. Dev	CoV(%)	Posterior Min	Posterior Max	PSO Derived Model	Experimental Target
Cohesive Energy	-30.718	0.382	0.6	-31.1	-30.336	-30.395	-31.28
Poisson's Ratio	0.417	0.006	0.7	0.411	0.423	0.413	0.374
C12	170.657	4.274	1.3	166.383	174.931	169.301	176
C11	238.612	4.822	1	233.79	243.434	240.628	234.1

Bulk Modulus	193.466	3.856	1	189.61	197.322	193.077	193
C44	68.041	4.728	3.5	63.313	72.769	71.328	71.2
ST100	2060.409	21.56	0.5	2038.849	2081.969	2070.414	2059.2
Density	11.942	0.4	1.7	11.542	12.342	11.994	12.023

Table S25. A comparison of values defining 95 % credible intervals for properties obtained for Au metal.

Au	Mean	2 Std. Dev	CoV	PSO	Target
Cohesive Energy	-28.364	0.162	0.003	-27.8	-30.48
Poisson's Ratio	0.44	0.002	0.003	0.427	0.42
C12	158.419	3.72	0.012	155.67	163
C11	198.818	3.758	0.009	208.4	192
Bulk Modulus	172.852	3.652	0.011	52.73	42.3
C44	43.207	1.38	0.016	173.25	173
ST100	1538.343	16.41	0.005	1538.78	1540
Density	19.24	0.378	0.01	19.89	19.3

Table S26. A comparison of values defining 95 % credible intervals for properties obtained for Ag metal.

Ag	Mode 1			Mode 2			PSO	Target
	Mean	2 Std. Dev	CoV	Mean	2 Std. Dev	CoV		
Cohesive Energy	-23.627	0.296	0.006	-23.829	0.276	0.006	-23.827	-23.776
Poisson's Ratio	0.399	0.004	0.004	0.4	0.004	0.004	0.397	0.337
C12	87.683	2.01	0.011	88.618	2.012	0.011	88.627	93.67
C11	132.229	2.588	0.01	132.198	2.476	0.009	134.8	123.99

Bulk Modulus	102.529	2.116	0.01	103.203	2.05	0.01	104.018	104
C44	44.302	1.486	0.017	43.677	1.63	0.019	46.174	46.12
ST100	1252.709	21.264	0.008	1240.402	21.234	0.009	1251.761	1237.6
ST111	1039.638	8.738	0.004	1034.703	6.9	0.003	1193.615	1190
Density	10.21	0.228	0.011	10.631	0.22	0.01	10.295	10.49

Table S27. A comparison of values defining 95 % credible intervals for properties obtained for Cu metal.

Cu	Mode 1			Mode 2			PSO	Target
	Mean	2 Std. Dev	CoV	Mean	2 Std. Dev	CoV		
Cohesive Energy	-28.346	0.418	0.007	-28.298	0.558	0.01	-28.228	-28.192
Poisson's Ratio	0.381	0.004	0.005	0.382	0.004	0.006	0.374	0.323
C12	111.379	2.608	0.012	111.71	2.668	0.012	111.852	121.4
C11	181.154	3.916	0.011	180.671	4.05	0.011	187.177	168.4
Bulk Modulus	134.658	2.83	0.011	134.719	2.874	0.011	136.961	137
C44	69.876	2.7	0.019	69.132	2.938	0.021	75.324	75.4
Density	8.756	0.224	0.013	9.084	0.264	0.015	8.934	8.96

Table S28. A comparison of values defining 95 % credible intervals for properties obtained for Pt metal.

Pt	Mode 1			Mode 2			PSO	Target
	Mean	2 Std. Dev	CoV	Mean	2 Std. Dev	CoV		
Cohesive Energy	-43.469	0.546	0.006	-43.437	0.594	0.007	-42.228	-46.72
C12	255.487	6.968	0.014	255.39	6.934	0.014	257.267	251
C11	337.667	9.852	0.015	337.904	9.58	0.014	333.084	347

Bulk Modulus	282.612	6.644	0.012	282.714	6.488	0.011	282.54	283
C44	83.767	9.762	0.058	83.49	9.532	0.057	75.817	76
Density	22.283	1.568	0.035	22.551	1.384	0.031	23.543	21.46

Section S5 Validation of GPR Model Predicted Properties via CG MD Simulations

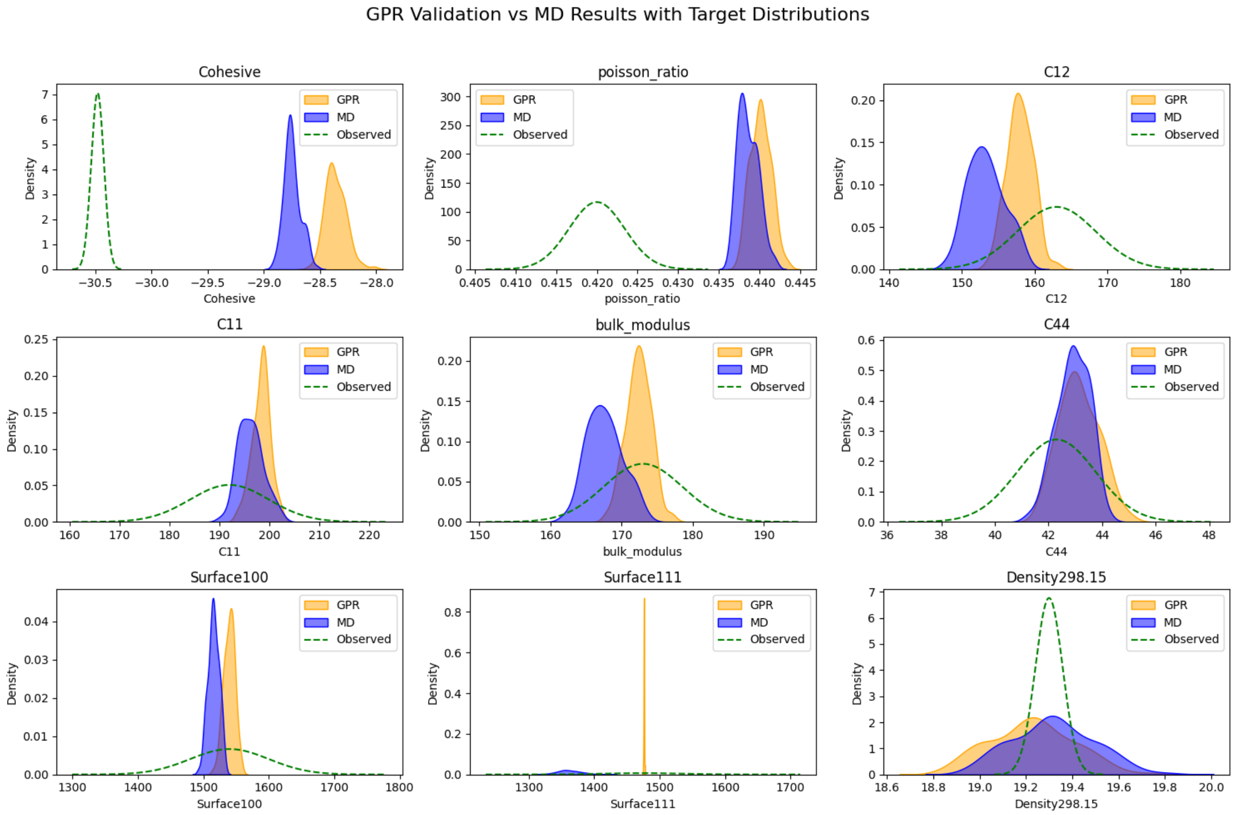

Figure S47. The comparison of GP predictions with the MD validation of 100 posterior points for Au.

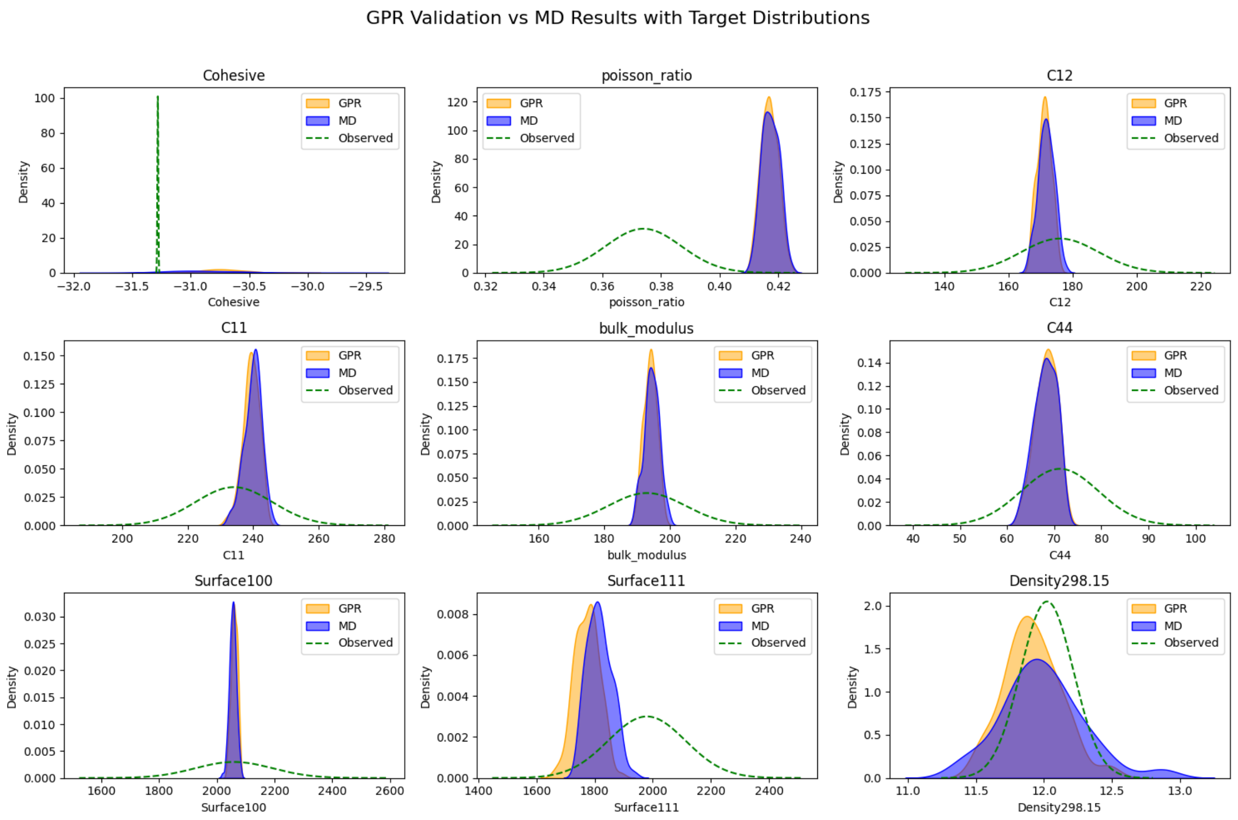

Figure S48. The comparison of GP predictions with the MD validation of 100 posterior points for Pd.

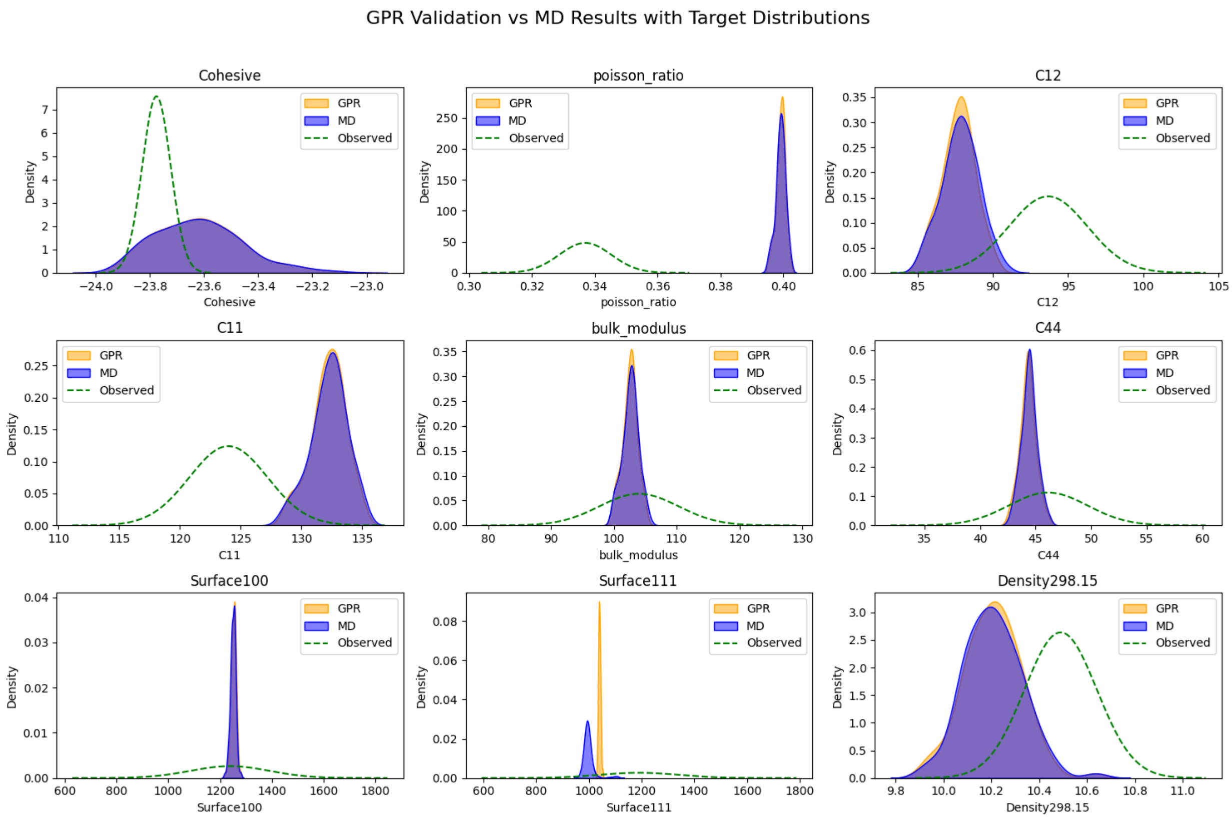

Figure S49. The comparison of GP predictions with the MD validation of 100 posterior points for Ag Mode 1.

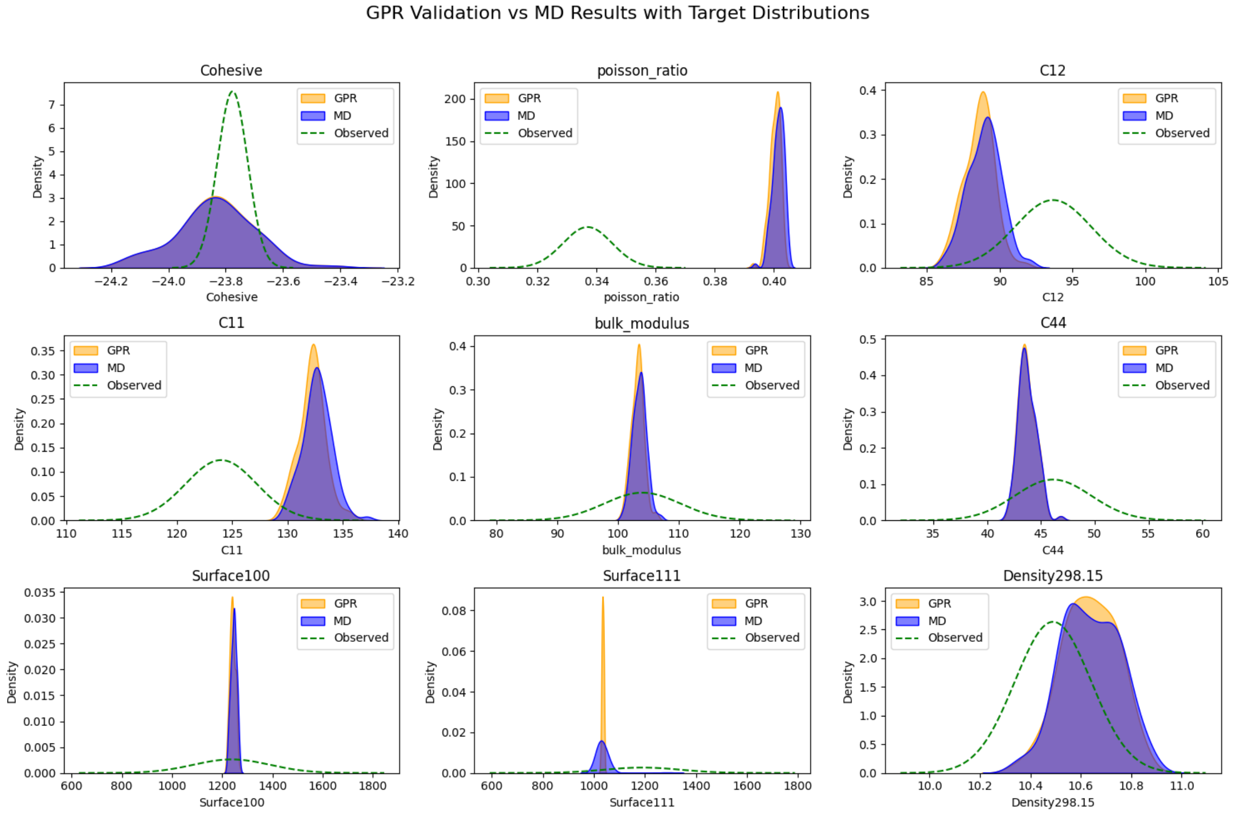

Figure S50. The comparison of GP predictions with the MD validation of 100 posterior points for Ag Mode 2.

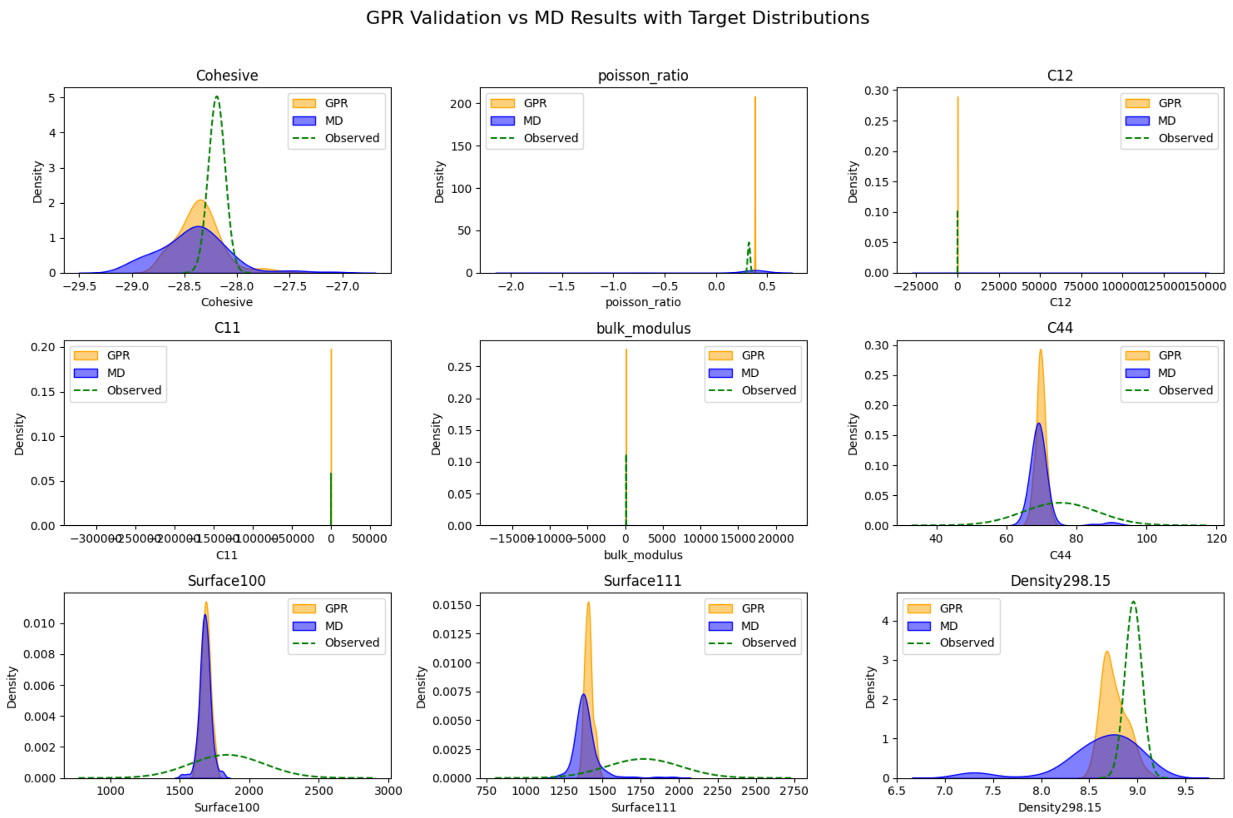

Figure S51. The comparison of GP predictions with the MD validation of 100 posterior points for Cu Mode 1.

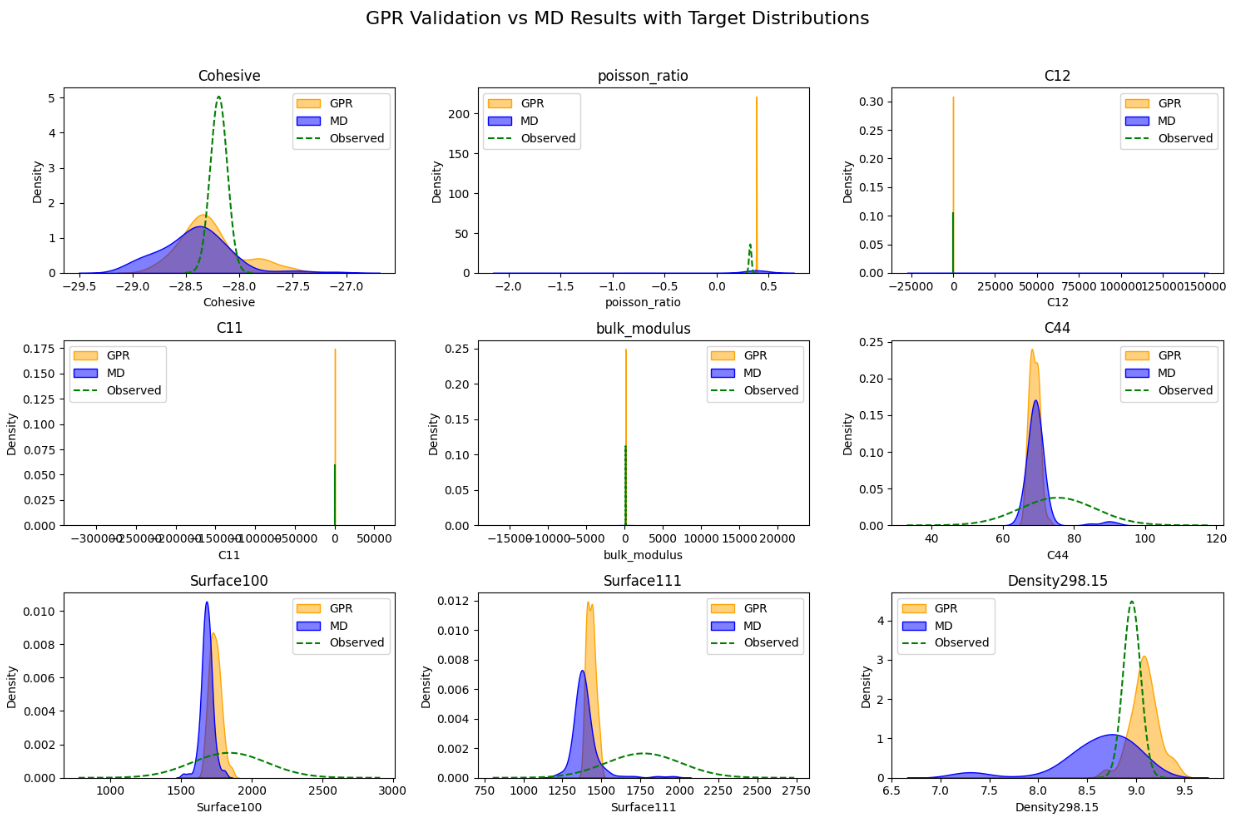

Figure S52. The comparison of GP predictions with the MD validation of 100 posterior points for Cu Mode 2.

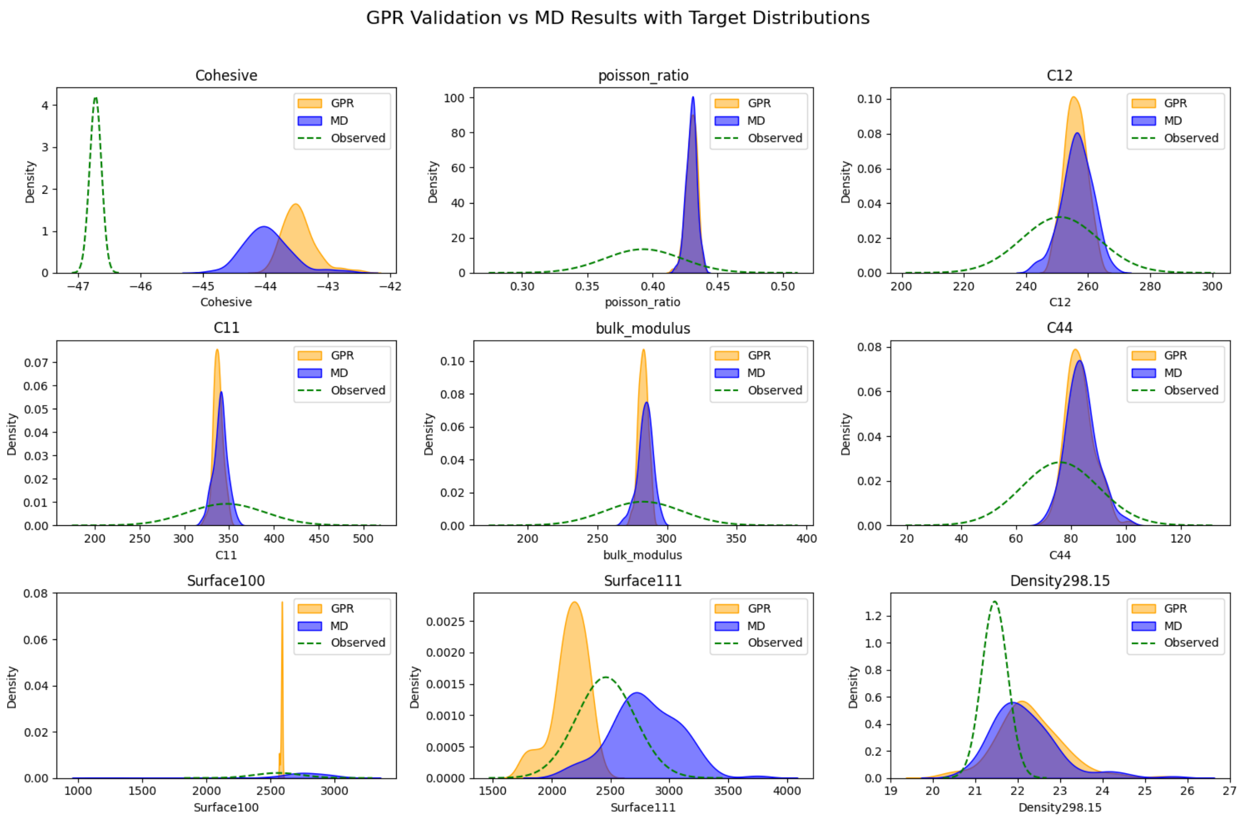

Figure S53. The comparison of GP predictions with the MD validation of 100 posterior points for Pt Mode 1.

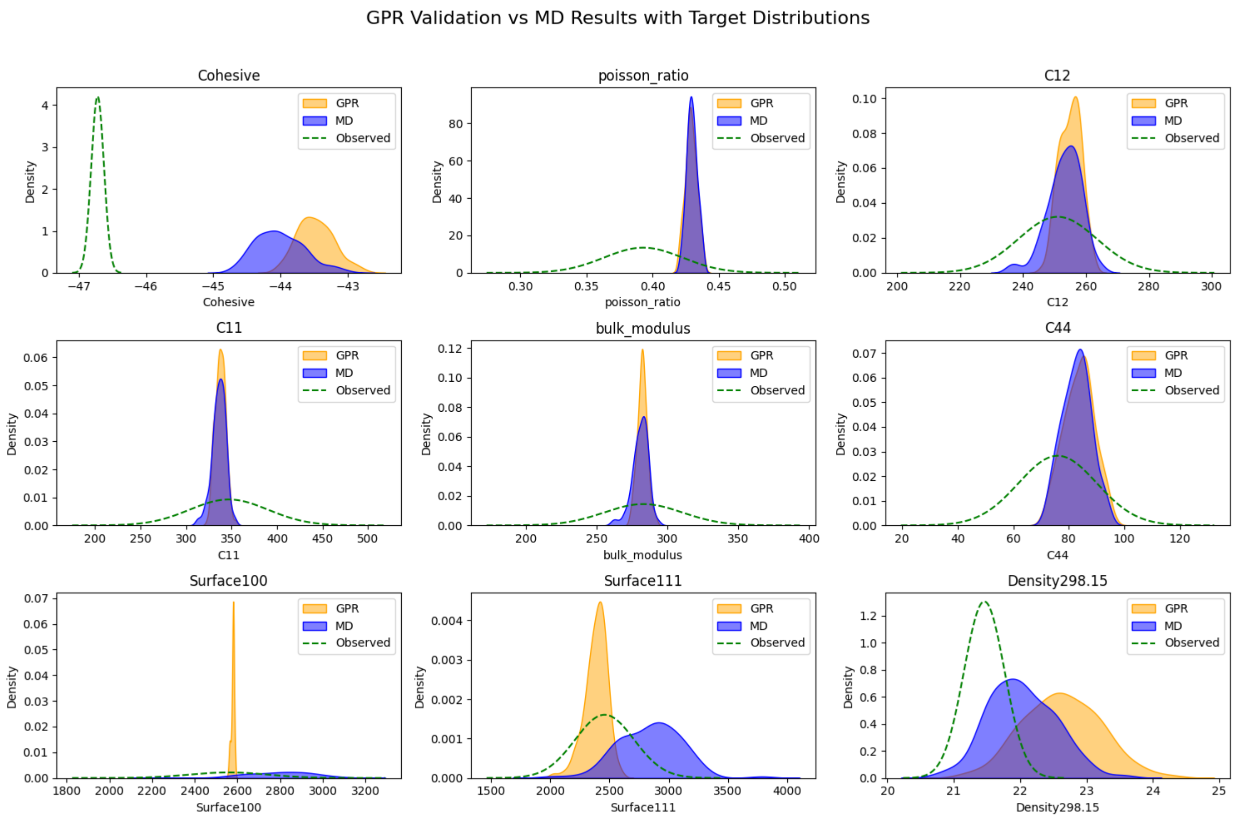

Figure S54. The comparison of GP predictions with the MD validation of 100 posterior points for Pt Mode 2.

Section S6. Comparison of MD simulations with experiments:

In order to showcase the proficiency of CG modeling, we performed CG MD simulations with a timestep of 1fs, 5fs, 10fs, and 20fs on 100 randomly selected GC EAM parameter sets obtained from posterior distribution post BUQ analysis.

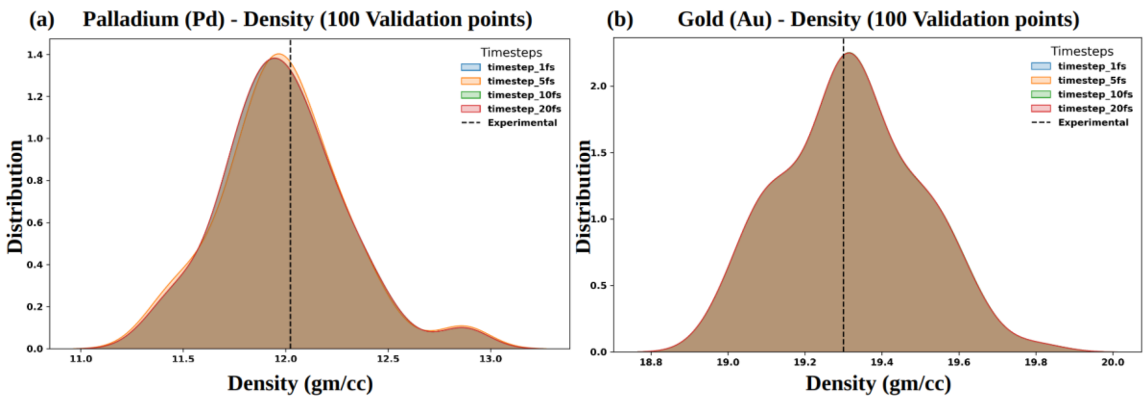

Figure S55. The comparison of the effect of increasing the time step from 1fs to 5fs, 10fs, and 20fs on density calculation from MD simulations. The experimental values are used as a reference. The distribution of density values of MD simulations of 100 randomly sampled parameters. The dotted line represents the experimental target value for **(a)** Pd (Palladium) and **(b)** Au (gold).

We calculated the density of the final 0.1 ns of the total 1 ns. And then we plot the distribution of absolute error deviation of density calculated at 1fs, 5 fs, 10 fs, and 20 fs time step deviating from the experimental values (12.023 gm/cc for Pd and 19.3 gm/cc for Au) in **Figure S55 (a)** (for Pd) and **Figure S55 (b)** (for Au). For Pd, the density values showed only minimal changes as we increased the timestep from 1fs to 5 fs and till 10 fs. However, we observed a right shift as exhibited by the red graph in **Figure S55 (a)** for 20fs time step simulations indicating an increase in the absolute errors and with an increase in the timestep the density reduces. On the other hand we also observed that increase in timestep for MD simulations hardly altered the density values for gold. This was evident by perfect overlay of the distributions for all 4 timesteps in **Figure S55 (b)**. These distributions exhibit the proficiency of CG simulations in accelerating time- and length scales in MD simulations. Using these CG force fields, micro- to milli-second time scales on a few hundreds of nanometers system sizes can be studied with minimal computational resources within reasonable real-time.

References:

(1) Daw, M. S.; Baskes, M. I. Embedded-Atom Method: Derivation and Application to Impurities, Surfaces, and Other Defects in Metals. *Phys. Rev. B Condens. Matter* **1984**, *29* (12), 6443–6453.

(2) Johnson, R. A. Alloy Models with the Embedded-Atom Method. *Phys. Rev. B Condens. Matter* **1989**, *39* (17), 12554–12559.

(3) Shan, B.; Wang, L.; Yang, S.; Hyun, J.; Kapur, N.; Zhao, Y.; Nicholas, J. B.; Cho, K. First-Principles-Based Embedded Atom Method for PdAu Nanoparticles. *Phys. Rev. B Condens. Matter* **2009**, *80* (3), 035404.

(4) Thompson, A. P.; Aktulga, H. M.; Berger, R.; Bolintineanu, D. S.; Brown, W. M.; Crozier, P. S.; in ’t Veld, P. J.; Kohlmeyer, A.; Moore, S. G.; Nguyen, T. D.; Shan, R.; Stevens, M. J.; Tranchida, J.; Trott, C.; Plimpton, S. J. LAMMPS - a Flexible Simulation Tool for Particle-Based Materials Modeling at the Atomic, Meso, and Continuum Scales. *Comput. Phys. Commun.* **2022**, *271*, 108171.

(5) Sheng, H. W.; Kramer, M. J.; Cadien, A.; Fujita, T.; Chen, M. W. Highly Optimized Embedded-Atom-Method Potentials for Fourteen Fcc Metals. *Phys. Rev. B Condens. Matter Mater. Phys.* **2011**, *83* (13). https://doi.org/10.1103/physrevb.83.134118.

(6) Rayne, J. A. Elastic Constants of Palladium from 4.2-300°K. *Phys. Rev.* **1960**, *118* (6),

1545–1549.
(7) Simmons, G.; Wang, H. *Single Crystal Elastic Constants and Calculated Aggregate Properties*, 2nd ed.; MIT Press: London, England, 1971.
(8) Heinz, H.; Vaia, R. A.; Farmer, B. L.; Naik, R. R. Accurate Simulation of Surfaces and Interfaces of Face-Centered Cubic Metals Using 12−6 and 9−6 Lennard-Jones Potentials. *J. Phys. Chem. C Nanomater. Interfaces* **2008**, *112* (44), 17281–17290.
(9) Tyson, W. R.; Miller, W. A. Surface Free Energies of Solid Metals: Estimation from Liquid Surface Tension Measurements. *Surf. Sci.* **1977**, *62* (1), 267–276.
(10) *Technical data for the element Palladium in the Periodic Table*. https://periodictable.com/Elements/046/data.html (accessed 2025-07-16).
(11) Kittel C. *Introduction to Solid State Physics*; Wiley, 1959.
(12) *Gold, Au*. https://www.matweb.com/search/datasheet.aspx?bassnum=AMEAu00&ckck=1 (accessed 2025-07-16).
(13) *Gold - Properties and Applications of Gold*. AZoM. https://www.azom.com/properties.aspx?ArticleID=598 (accessed 2025-07-16).
(14) Brandes, E. A.; Brook, G. B.; Paufler, P. Smithells Metals Reference Book. Butterworth-Heinemann Ltd., Oxford, 1992. 1746 Seiten, Preis 150 £, ISBN 0-7506-1020-4. *Cryst. Res. Technol.* **1993**, *28* (4), 530–530.
(15) Yoneda, A.; Fukui, H.; Gomi, H.; Kamada, S.; Xie, L.; Hirao, N.; Uchiyama, H.; Tsutsui, S.; Baron, A. Q. R. Single Crystal Elasticity of Gold up to ~20 GPa: Bulk Modulus Anomaly and Implication for a Primary Pressure Scale. *Jpn. J. Appl. Phys.* **2017**, *56* (9), 095801.
(16) Brown, T. L.; Eugene LeMay, H.; Bursten, B. E. *Chemistry: The Central Science*; Prentice Hall, 2012.
(17) Cutnell, J. D.; Johnson, K. W. *Physics*; John Wiley & Sons, 2009.
(18) Pearson, W. B.; Vineyard, G. H. *A Handbook of Lattice Spacings and Structures of Metals and Alloys*. *Phys. Today* **1958**, *11* (9), 36–36.
(19) Simmons, G.; Wang, H. *Single Crystal Elastic Constants and Calculated Aggregate Properties: A Handbook*; MIT Press: Cambridge, MA, 1971.
(20) Lide, D. R. *CRC Handbook of Chemistry and Physics, 84th Edition*, 84th ed.; CRC Press: Boca Raton, FL, 2003.
(21) *Silver density*. https://kg-m3.com/material/silver (accessed 2025-07-16).
(22) Kambe, K. Cohesive Energy of Noble Metals. *Phys. Rev*. **1955**, *99* (2), 419–422.
(23) Overton, W. C.; Gaffney, J. Temperature Variation of the Elastic Constants of Cubic Elements. I. Copper. *Phys. Rev*. **1955**, *98* (4), 969–977.
(24) Chen, F. *Understanding the Mass Density of Copper: kg/m^3 Explained*. BOYI. https://www.boyiprototyping.com/materials-guide/density-of-copper/ (accessed 2025-06-25).
(25) Smithells, C. J. *Metals Reference Book*; Smithells, C. J., Ed.; Butterworth-Heinemann: Woburn, MA, 2013.
(26) Kirke Rose, T. *The Precious Metals: Comprising Gold, Silver and Platinum (classic Reprint) the Precious Metals: Comprising Gold, Silver and Platinum (classic Reprint)*; Forgotten Books: London, England, 2022.
(27) Adams, J. B.; Foiles, S. M.; Wolfer, W. G. Self-Diffusion and Impurity Diffusion of Fcc Metals Using the Five-Frequency Model and the Embedded Atom Method. *J. Mater. Res.* **1989**, *4* (1), 102–112.
(28) Foiles, S. M.; Baskes, M. I.; Daw, M. S. Embedded-Atom-Method Functions for the Fcc

Metals Cu, Ag, Au, Ni, Pd, Pt, and Their Alloys. *Phys. Rev. B Condens. Matter* **1986**, *33* (12), 7983–7991.
(29) Foiles, S. M.; Baskes, M. I.; Daw, M. S. Erratum: Embedded-Atom-Method Functions for the Fcc Metals Cu, Ag, Au, Ni, Pd, Pt, and Their Alloys. *Phys. Rev. B Condens. Matter* **1988**, *37* (17), 10378–10378.
(30) Hale, L. M.; Wong, B. M.; Zimmerman, J. A.; Zhou, X. W. Atomistic Potentials for Palladium–silver Hydrides. *Modell. Simul. Mater. Sci. Eng.* **2013**, *21* (4), 045005.
(31) Zhou, X. W.; Zimmerman, J. A.; Wong, B. M.; Hoyt, J. J. An Embedded-Atom Method Interatomic Potential for Pd–H Alloys. *J. Mater. Res.* **2008**, *23* (3), 704–718.
(32) Zhou, X. W.; Wadley, H. N. G.; Johnson, R. A.; Larson, D. J.; Tabat, N.; Cerezo, A.; Petford-Long, A. K.; Smith, G. D. W.; Clifton, P. H.; Martens, R. L.; Kelly, T. F. Atomic Scale Structure of Sputtered Metal Multilayers. *Acta Mater.* **2001**, *49* (19), 4005–4015.
(33) Zhou, X. W.; Johnson, R. A.; Wadley, H. N. G. Misfit-Energy-Increasing Dislocations in Vapor-Deposited CoFe/NiFe Multilayers. *Phys. Rev. B Condens. Matter* **2004**, *69* (14), 144113.
(34) Lee, B.-J.; Shim, J.-H.; Baskes, M. I. Semiempirical Atomic Potentials for the Fcc Metals Cu, Ag, Au, Ni, Pd, Pt, Al, and Pb Based on First and Second Nearest-Neighbor Modified Embedded Atom Method. *Phys. Rev. B Condens. Matter* **2003**, *68* (14), 144112.
(35) Jacobsen, K. W.; Stoltze, P.; Nørskov, J. K. A Semi-Empirical Effective Medium Theory for Metals and Alloys. *Surf. Sci.* **1996**, *366* (2), 394–402.
(36) Sose, A. T.; Gustke, T.; Wang, F.; Anand, G.; Pasupuleti, S.; Savara, A.; Deshmukh, S. A. Evaluation of Sampling Algorithms Used for Bayesian Uncertainty Quantification of Molecular Dynamics Force Fields. *J. Chem. Theory Comput.* **2024**, *20* (13), 5732–5742.
(37) Ackland, G. J.; Tichy, G.; Vitek, V.; Finnis, M. W. Simple N-Body Potentials for the Noble Metals and Nickel. *Philos. Mag. A* **1987**, *56* (6), 735–756.
(38) Gola, A.; Pastewka, L. Embedded Atom Method Potential for Studying Mechanical Properties of Binary Cu–Au Alloys. *Modell. Simul. Mater. Sci. Eng.* **2018**, *26* (5), 055006.
(39) Grochola, G.; Russo, S. P.; Snook, I. K. On Fitting a Gold Embedded Atom Method Potential Using the Force Matching Method. *J. Chem. Phys.* **2005**, *123* (20), 204719.
(40) Olsson, P. A. T. Transverse Resonant Properties of Strained Gold Nanowires. *J. Appl. Phys.* **2010**, *108* (3), 034318.
(41) Purja Pun, G. *EAM Potential (LAMMPS Cubic Hermite Tabulation) for Au Developed by Pun (2017) v000*; OpenKIM, 2018. https://doi.org/10.25950/B433CD2B.
(42) Zhakhovskii, V. V.; Inogamov, N. A.; Petrov, Y. V.; Ashitkov, S. I.; Nishihara, K. Molecular Dynamics Simulation of Femtosecond Ablation and Spallation with Different Interatomic Potentials. *Appl. Surf. Sci.* **2009**, *255* (24), 9592–9596.
(43) Pan, Z.; Borovikov, V.; Mendelev, M. I.; Sansoz, F. Development of a Semi-Empirical Potential for Simulation of Ni Solute Segregation into Grain Boundaries in Ag. *Modell. Simul. Mater. Sci. Eng.* **2018**, *26* (7), 075004.
(44) Williams, P. L.; Mishin, Y.; Hamilton, J. C. An Embedded-Atom Potential for the Cu–Ag System. *Modell. Simul. Mater. Sci. Eng.* **2006**, *14* (5), 817.
(45) Wu, H. H.; Trinkle, D. R. Cu/Ag EAM Potential Optimized for Heteroepitaxial Diffusion from Ab Initio Data. *Comput. Mater. Sci.* **2009**, *47* (2), 577–583.
(46) Alvi, S. M. A. A.; Faiyad, A.; Munshi, M. A. M.; Motalab, M.; Islam, M. M.; Saha, S. Cyclic and Tensile Deformations of Gold–Silver Core Shell Systems Using Newly Parameterized MEAM Potential. *Mech. Mater.* **2022**, *169*, 104304.

(47) Ackland, G.; Tichy, G.; Vitek, V.; Finnis, M. *Finnis-Sinclair Potential (LAMMPS Cubic Hermite Tabulation) for Cu Developed by Ackland et Al. (1987) v005*; OpenKIM, 2018. https://doi.org/10.25950/7FE84E8C.
(48) Foiles, S.; Adams, J.; Wolfer, W. *EAM Potential (LAMMPS Cubic Hermite Tabulation) for Cu (Universal6) Developed by Adams, Foiles, and Wolfer (1989) v000*; OpenKIM, 2018. https://doi.org/10.25950/59E45524.
(49) Foiles, S.; Baskes, M.; Daw, M. *EAM Potential (LAMMPS Cubic Hermite Tabulation) for Cu (Universal3) Developed by Foiles, Baskes, and Daw (1986) v004*; OpenKIM, 2018. https://doi.org/10.25950/9AD4E749.
(50) Foiles, S. *EAM Potential (LAMMPS Cubic Hermite Tabulation) for Cu Developed by Foiles (1985) for NiCu Alloys v000*; OpenKIM, 2018. https://doi.org/10.25950/C559B4C8.
(51) Mendelev, M. I.; King, A. H. The Interactions of Self-Interstitials with Twin Boundaries. *Philos. Mag. (Abingdon)* **2013**, *93* (10-12), 1268–1278.
(52) Mendelev, M. I.; Kramer, M. J.; Becker, C. A.; Asta, M. Analysis of Semi-Empirical Interatomic Potentials Appropriate for Simulation of Crystalline and Liquid Al and Cu. *Philos. Mag. (Abingdon)* **2008**, *88* (12), 1723–1750.
(53) Mishin, Y.; Mehl, M. J.; Papaconstantopoulos, D. A.; Voter, A. F.; Kress, J. D. Structural Stability and Lattice Defects in copper:Ab Initio, Tight-Binding, and Embedded-Atom Calculations. *Phys. Rev. B Condens. Matter* **2001**, *63* (22). https://doi.org/10.1103/physrevb.63.224106.
(54) Elliott, R.; Johnson, R. *EAM Potential (analytical Nearest-Neighbor) for Cu Developed by Johnson (1988) v003*; OpenKIM, 2022. https://doi.org/10.25950/26B6F589.
(55) Zuo, Y.; Chen, C.; Li, X.; Deng, Z.; Chen, Y.; Behler, J.; Csányi, G.; Shapeev, A. V.; Thompson, A. P.; Wood, M. A.; Ong, S. P. Performance and Cost Assessment of Machine Learning Interatomic Potentials. *J. Phys. Chem. A* **2020**, *124* (4), 731–745.
(56) Asadi, E.; Asle Zaeem, M.; Nouranian, S.; Baskes, M. I. Two-Phase Solid–liquid Coexistence of Ni, Cu, and Al by Molecular Dynamics Simulations Using the Modified Embedded-Atom Method. *Acta Mater.* **2015**, *86*, 169–181.
(57) O'Brien, C. J.; Barr, C. M.; Price, P. M.; Hattar, K.; Foiles, S. M. Grain Boundary Phase Transformations in PtAu and Relevance to Thermal Stabilization of Bulk Nanocrystalline Metals. *J. Mater. Sci.* **2018**, *53* (4), 2911–2927.
(58) Neal, R. M. Slice Sampling. *Ann. Stat.* **2003**, *31* (3), 705–767.
(59) Goodman, J.; Weare, J. Ensemble Samplers with Affine Invariance. *Communications in Applied Mathematics and Computational Science* **2010**, *5* (1), 65–80.
(60) Foreman-Mackey, D.; Hogg, D. W.; Lang, D.; Goodman, J. Emcee: The MCMC Hammer. *arXiv [astro-ph.IM]*, 2012. http://arxiv.org/abs/1202.3665.
(61) Karamanis, M.; Beutler, F. Ensemble Slice Sampling: Parallel, Black-Box and Gradient-Free Inference for Correlated & Multimodal Distributions. *arXiv [stat.ML]*, 2020. http://arxiv.org/abs/2002.06212.
